# From knowledge production to knowledge translation: Waterpipe tobacco control research in the Eastern Mediterranean Region

**DOI:** 10.18332/tpc/175953

**Published:** 2024-01-19

**Authors:** Dina Farran, Ramzi G. Salloum, Fadi El Jardali, Ruba Abla, Niveen Abu Rmeileh, Nihaya Al Sheyab, Sameera Awaw-da, Ali Chalak, Mohammed Jawad, Yousef Khader, Aya Mostafa, Rima Nakkash

**Affiliations:** 1 Department of Psychosis Studies, Institute of Psychiatry, Psychology and Neuroscience, King’s College London, London, UK; 2 Department of Health Outcomes & Biomedical Informatics, University of Florida College of Medicine, Gainesville, Florida, USA; 3 Health Management and Policy Department, Faculty of Health Sciences, American University of Beirut, Lebanon; 4 Department of Health Promotion and Community Health, Faculty of Health Sciences, American University of Beirut, Lebanon; 5 Institute of Community and Public Health, Birzeit University, Birzeit, Palestine; 6 Faculty of Applied Medical Sciences, Jordan University of Science and Technology, Irbid, Jordan; 7 Department of Agriculture, Faculty of Agricultural and Food Sciences, American University of Beirut, Beirut, Lebanon; 8 Public Health Policy Evaluation Unit, Imperial College London School of Public Health, London, UK; 9 Department of Community Medicine, Public Health and Family Medicine, Faculty of Medicine, Jordan University of Science and Technology, Irbid, Jordan; 10 Department of Community, Environmental and Occupational Medicine, Faculty of Medicine, Ain Shams University, Cairo, Egypt; 11 Global and Community Health Department, George Mason University, Virginia, USA; 12 Economics Department, Faculty of Business and Economics, Birzeit University, Birzeit, Palestine

**Keywords:** knowledge translation, tobacco control, health policy, public health, waterpipe

## Abstract

Waterpipe tobacco smoking (WTS) rates in the Eastern Mediterranean Region (EMR) are the highest worldwide, particularly among young people. Although fiscal policies to curb tobacco use have been recommended by the World Health Organization (WHO) Framework Convention on Tobacco Control (FCTC), implementation has been suboptimal. The Eastern Mediterranean Consortium on the Economics of Waterpipe Tobacco Smoking (ECON-WTS) was formed in response to this need to produce knowledge on the economics of WTS in the EMR and apply a comprehensive Knowledge translation (KT) framework. The KT framework comprised priority setting, evidence synthesis, knowledge translation, and knowledge uptake. In this article, we discuss the approaches followed in applying the KT framework to WTS control, providing examples and noting challenges and lessons learned where possible.

## INTRODUCTION

Waterpipe tobacco smoking (WTS) rates in the Eastern Mediterranean Region (EMR) are the highest worldwide, particularly among young people^1^. In August 2019, household surveys were conducted with participants aged >18 years in Lebanon (n=1680), Jordan (n=1925), and Palestine (n=1679). Results showed that the prevalence of waterpipe smoking among males and females, respectively, was 32.7% and 46.2% in Lebanon, 13.4% and 7.8% in Jordan, and 18.0% and 7.9% in Palestine. Additionally, waterpipe smokers were more likely to be young adults across the three countries (p<0.001) and they were more likely to be male in Jordan and Palestine, and more likely to be female in Lebanon^1^. In Egypt, the latest STEPwise survey of non-communicable disease risk factors reported that waterpipe tobacco smoking prevalence in individuals aged 15–69 years was 8.7% in males and 0.1% in females^2^.

Waterpipe tobacco smoking has been rapidly increasing in the region over the past couple of decades, particularly among young people^3^ and those high prevalence rates are very alarming^1^. The EMR has a long history of political and ethnic conflicts, in addition to current weak public health systems^4^, which have weakened tobacco control efforts and contributed to the persistence of fragile tobacco regulations across most countries in the region^5^.

Fiscal policies to curb tobacco use have been recommended by the World Health Organization (WHO) Framework Convention on Tobacco Control (FCTC)^6^, yet their implementation has been suboptimal^7^. Thus, there is a critical need to support governments to develop effective fiscal policies to curb WTS^8^. Additionally, there are significant gaps in knowledge related to tobacco control research. Despite the rise in waterpipe smoking prevalence, research on effective interventions to address this trend has been limited^9^.

The use of evidence in health policymaking has been gaining a lot of recognition as it can reinforce and support health systems, improve population health, quicken progress on attaining the Sustainable Development Goals (SDGs), and improve population health^10-14^. In 2011, the WHO Eastern Mediterranean Regional Office (WHO EMRO) highlighted, the need for the development and implementation of research for health as a vital tool for health development and informing health policy^15^. Knowledge translation (KT) is defined as a dynamic and iterative method that includes the synthesis, dissemination, exchange and application of knowledge to improve the health of the population, deliver more effective health services, and reinforce the healthcare system^16^. It is characterized by the systematic and transparent access to, and use of, evidence as an input into the policymaking process^16,17^.

Experts from Lebanon, Jordan, Palestine and Egypt formed The Eastern Mediterranean Consortium on the economics of waterpipe tobacco smoking (ECON-WTS), in response to the need to address the knowledge of policy gaps in tobacco control in the region. Its objective is to produce knowledge on the economics of WTS in the EMR and apply a comprehensive KT framework. Collaborations involving professionals with knowledge, skills and expertise from multiple disciplines are key to producing high-quality evidence and policy recommendations^18^. Our Consortium consisted of researchers with experience in tobacco control and expertise in health economics, public health, epidemiology, health policy, biostatistics, health promotion, and knowledge synthesis, from Jordan, Palestine, Lebanon, and Egypt. Our KT framework comprised the domains of priority setting, evidence synthesis, knowledge translation, and knowledge uptake. The framework is impact-oriented, meaning that KT should be driven by the desired end results. KT is viewed as a continuum that commences from setting the research agenda to implementation into policy and evaluation, passing through knowledge production and translation. Stakeholder engagement and capacity building are seen as critical components of successful evidence-informed policymaking^19^.

## COMMENTARY

We discuss the approaches we followed in applying the KT framework to WTS control providing examples and noting challenges and lessons learned, where possible. Table 1 shows a summary of the components/elements that contributed to the success or failure of applying the KT framework approach to waterpipe tobacco smoking, on which we expand further below.

**Table 1:** Components/elements that contributed to the success or failure in applying the KT framework approach for waterpipe tobacco smoking

*Components/elements of success*	*Components/elements of failure*
Engaging leading academic institutions in the Consortium	Insufficient number and little collaboration of researchers willing to conduct tobacco control research
Collaborating with multidisciplinary and multi-institutional teams	Time allocated to researchers in developing KT tools not accounted as part of their academic workload
Engaging tobacco control experts	Paucity in waterpipe tobacco smoking research skill training
Access to databases, published articles, and systematic reviews	Limited research on waterpipe tobacco taxation nationally and regionally
Aligning knowledge production with policy priorities	Weak and fragmented governance and rule of law in some contexts
Participating in workshops and conferences to engage policymakers and stakeholders	Policymakers minimally engaged because of their misconception that increasing taxes would decrease government revenues
Engaging policymakers in the research process	Tobacco control not being a priority
Sharing research findings with policymakers	Policymaking culture giving little importance to evidence
Dissemination of knowledge into short, understandable and accessible products	Interference of the tobacco industry
Training and coaching for team members	Lack of financial resources to conduct KT

### Priority setting and evidence synthesis

Engaging leading academic institutions in the Consortium provided the infrastructure required for knowledge production as it ensured: 1) engagement of tobacco control experts with knowledge of the economic and cultural contexts; and 2) access to databases, published articles, and systematic reviews. Producing research that appeals to political agendas, while ensuring the integration of economic and cultural contexts, is crucial for evidence uptake^20^.

Implementing evidence-informed tobacco control policies remains the most sustainable and effective intervention to reduce tobacco use and its health burden^21^. The Consortium worked to align knowledge production with policy priorities. As an initial step, a review of the local and regional literature was conducted to identify documented policy priorities. Second, researchers assessed policymakers’ priorities, needs for evidence, and KT product preferences. The assessment served to identify policymakers willing to support evidence-informed policies, the nature of the political and economic challenges, and the extent of tobacco industry interference.

Sharing knowledge with other research groups working on tobacco facilitated priority setting and evidence synthesis. For example, the Jordanian team joined a group working on a tobacco project (The United Against Tobacco and COVID-Jordan) funded by the United States Centers for Disease Control and Prevention (CDC), in collaboration with the Global Health Development |Eastern Mediterranean Public Health Network (GHD|EMPHNET) and Vital Strategies, and in partnership with the Jordanian Ministry of Health (MOH), World Health Organization (WHO)-Jordan Country Office, and Greater Amman Municipality (GAM)^22^. The collaboration led to the introduction of the team members to others in the field of tobacco control research. Building networks was possible through participation in conferences, and workshops, as well as collaborations with Non-Governmental Organizations (NGOs) involved in tobacco control. Engaging multidisciplinary and multi-institutional teams to develop KT products led to successful dissemination of results. In Egypt, the team worked with NGOs, researchers, policy advocates, health professionals, WHO experts in tobacco economics and tobacco control experts in WHO EMRO, to review the validity and feasibility of the elements proposed for restructuring waterpipe tobacco taxation and to facilitate priority-setting and evidence synthesis. These diverse insights obtained during the development of the policy brief helped in capturing the best available evidence in appealing key messages to engage policymakers more effectively. Also, the Egyptian team arranged for stakeholders’ participation in a dedicated session, by a WHO tobacco economics expert, about best practices in tobacco taxation, discussing the differentials of waterpipe tobacco taxation in countries of the EMR. In addition, joining various research groups working on tobacco locally, regionally, and internationally offered an opportunity to build multi-disciplinary collaborations and engage people with different professional skills, knowledge, and social networks. Additionally, members of the research team from Lebanon were invited by the WHO and the Tobacconomics research group at the Institute for Health Research and Policy (IHRP), University of Illinois at Chicago (UIC), to participate in a workshop on ‘Regional Training and Capacity Building of Academia in Tobacco Economics and Research’. They were then invited by UIC to write a landscape report and policy brief on the economics of tobacco in Lebanon and present the findings at the UIC Think Tank Partners Meeting in Bali, Indonesia. The insights from this report were used to recommend directions for tobacco economics research for IHRP’s next funding cycle.

Very little research on waterpipe tobacco taxation nationally and regionally existed at the time the Consortium was founded. This was in turn overcome by leveraging global and international partners with similar expertise drawn from evidence on cigarette taxation. The lessons learned from this component is that such collaborations support priority setting and evidence synthesis.

### Knowledge translation

Dissemination and translation of knowledge into short, understandable and accessible products are also key for the development of evidence-informed tobacco control policies^21^. The KT products include policy briefs, briefing notes, rapid responses, media bites, and evidence summaries^23^.

The Consortium delivered workshops and provided coaching for team members on developing KT products, focusing mostly on the policy brief, as identified in the priority-setting stage. Country teams were also trained on methods for engaging citizens in health policy^24^ via the development of citizen consultation briefs that packaged the policy elements using innovative data visualization tools. The team in Egypt disseminated a policy brief contextualizing evidence and focusing on three main elements where the waterpipe tobacco tax structure could be improved to maximize public and economic benefits. This approach was key in positioning the brief as satisfying the policymakers’ need for increased tax revenues to support the government’s limited income and for meeting the global and local public health requirements under the Sustainable Development Goals and Egypt’s Vision 2030. The team in Lebanon disseminated a policy brief that simulated revenues from tobacco sin taxes, during the period when the government was developing the 2023 national budget in 2022 (The contribution can be found in the Supplementary file Document 1). The timing of the brief was important to inform economic and fiscal policy especially since the government is looking for ways to generate revenues (Supplementary file Documents 2, 3 and 4, for Jordan, Palestine and Egypt policy briefs). The team in Palestine launched a tobacco control campaign in partnership with Global Health Development and under the auspices of the Palestinian Ministry of Health. The campaign disseminated messages to reach smokers, which were broadcast on radio, television, and social media platforms. The campaign included a set of events, activities, and challenges via social media platforms, in addition to the production of radio spots, posters, awareness videos, fact sheets and social service announcements.

Among the challenges faced were the insufficient number of researchers across various sectors willing to partner to conduct tobacco control research, the limited capacity of researchers to conduct knowledge translation given that they are trained primarily in scientific writing, which entails different skill sets, and the different perspectives of researchers with different theoretical backgrounds. Moreover, time allocated to researchers in developing KT tools is not accounted for as part of their academic workload. There is a dire need to provide capacity building for researchers across different domains, especially since KT is a vital strategy in ensuring that their research findings have an impact.

### Knowledge uptake

Policymakers and stakeholder engagement in research is crucial as they possess hands on experience in the context in which the findings may be used^18^. This adds valuable input to the research process and could shape the aims, objectives and design of the studies^17^. Community partnerships also help in the production of relevant evidence which results in the dissemination of materials beyond academia, enhances the usability of the findings, and reduces the time lag between knowledge production and uptake^25^.

To establish dialogue and engage policymakers and stakeholders, the Consortium participated in workshops and conferences (EMPHNET 7th regional conference, 26th EMPHNET Webinar)^22^ attended by stakeholders. Research findings were shared with policymakers via social media, webinars, and personal communication emails. Involving decision-makers and stakeholders in research provided them with a better understanding of the findings, promoted the sense of co-ownership of results and eventually made them think about its potential use in practice. This engagement also created a trusting relationship which underlined the legitimacy of the findings. For example, the Egyptian team partnered with Egypt’s Observatory for Tobacco Control (an NGO funded by the WHO) to engage a wide range of stakeholders involved in tobacco control research, activities, and policy-making in discussing the policy brief and advocating for restructuring waterpipe tobacco tax system in Egypt during a policy dialogue. As a result, the Egyptian team was able to work with different NGOs, such as the Cairo Association against Smoking, Tuberculosis and Chest Diseases Egypt (CASTLE), Committee on the Right to Health, Non-communicable Diseases Alliance, Health Masr, Tobacco-Free Life, and consulted with tobacco economic experts from the WHO, members of political parties in contact with legislators from the House of Representatives and the Senate (Egyptian Social Democratic Party), healthcare providers (Nursing Syndicate, Health Insurance Authority, consultant physicians, university hospitals), research bodies (Egyptian Smoking Prevention Research Institute), Ministries and Government Agencies (Health and Population, Social Solidarity (Addiction Treatment and Abuse Fund), Industry and Trade, Finance, Tax Authority), and the Presidential Advisor of the Public Health and Prevention Affairs. This multisectoral input from a wide array of local and international tobacco control stakeholders strengthened the KT uptake and led to successful outputs of the policy dialogue and a clear action plan involving relevant stakeholders in short-, intermediate-, and long-term actions to achieve the recommended policy change.

However, the Consortium also faced challenges in engaging policymakers in the EMR and getting their buy-in on tobacco taxation. These challenges included tobacco control not being a priority, weak and fragmented governance, and the rule of law in some contexts, little collaboration among tobacco control researchers, lack of research skills, reluctance to conduct research, and inadequate access to the most recent research findings. Overall, policymakers were minimally engaged, preferred decisions with immediate impact, and had the misconception

that increasing taxes would decrease government revenues (a claim not built on evidence and propagated by the tobacco industry). In addition, interference from tobacco companies, a weak rule of law, and a policymaking culture that gives little importance to evidence, stifled the process^26-28^.

It is important to persuade policymakers of the importance of informing policy with evidence. Involving policymakers in priority setting has been shown to increase the acceptability of evidence and foster its utilization in the decision-making process^24^.

**Figure 1:**
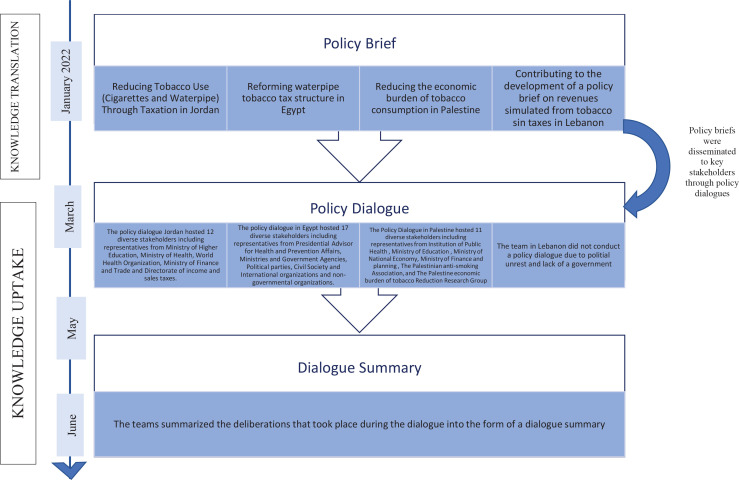
Summary of knowledge translation and knowledge uptake activities conducted by the Consortium

Figure 1 shows the activities conducted by the consortium for knowledge translation and knowledge uptake.

## CONCLUSION

This article summarizes our experiences in applying a knowledge translation framework to the generation of evidence around the economics of waterpipe tobacco smoking. Engaging multidisciplinary and multi-institutional teams with strong leadership skills, expertise, and readiness to share knowledge was a strength that promoted knowledge production, synthesis, translation and uptake. However, the limited investment of EMR governments in tobacco control and the lack of technical and financial resources to conduct KT, contributed to setbacks across all stages of the framework. To promote better uptake of tobacco control research evidence, it is important to align knowledge production with policy priorities, engage decision-makers in priority setting, develop researchers’ KT skills, improve communication of research findings, and strengthen interaction with decision-makers. More effort should be made to contextualize KT strategies and to evaluate their impact, particularly in settings where policymaking is not always evidence-based.

## Data Availability

Data sharing is not applicable to this article as no new data were created.
