# Supplementary material for: From knowledge production to knowledge translation: Waterpipe tobacco control research in the Eastern Mediterranean Region
Source: Tob Prev Cessat. 2024 Jan 19;10:10.18332/tpc/175953. doi: 10.18332/tpc/175953 (PMC10797642; doi:10.18332/tpc/175953)
Supplement: Supplementary file 1 [file TPC-10-03-s1.pdf]

Document 1

Cigarette and waterpipe tobacco taxes in Lebanon: preliminary results of a simulation model

Aim

To identify the impacts of specific excise duty changes on government revenue received from cigarettes and waterpipe tobacco in Lebanon

Methods

We developed a simulation model using Lebanon-specific tobacco market shares and their prices, consumption levels, and price elasticities of demand using routinely available and nationally representative surveys. We modelled $0.05 incremental changes in the specific tax and calculated the resulting impact on total tax revenues (taxes from import duty, excise duty, and VAT) and the number of cigarettes/waterpipes for two scenarios. Scenario 1 applied specific taxes only to imported cigarettes, and Scenario 2 applied specific taxes to all cigarettes. Both scenarios applied specific taxes to all waterpipe tobacco products.

Results

Under current tax structures ($0.17 specific tax for imported cigarettes and $0.03 specific tax for waterpipe tobacco), $1.2bn is raised in government revenue annually. The graph below shows the benefits on government revenue and public health benefits on the quantity of tobacco consumed, by increasing the specific tax. More government revenue can be realised by implementing a specific tax on all cigarettes (Scenario 2) instead of only imported cigarettes (Scenario 1), and the public health benefits on cigarette use are substantial.

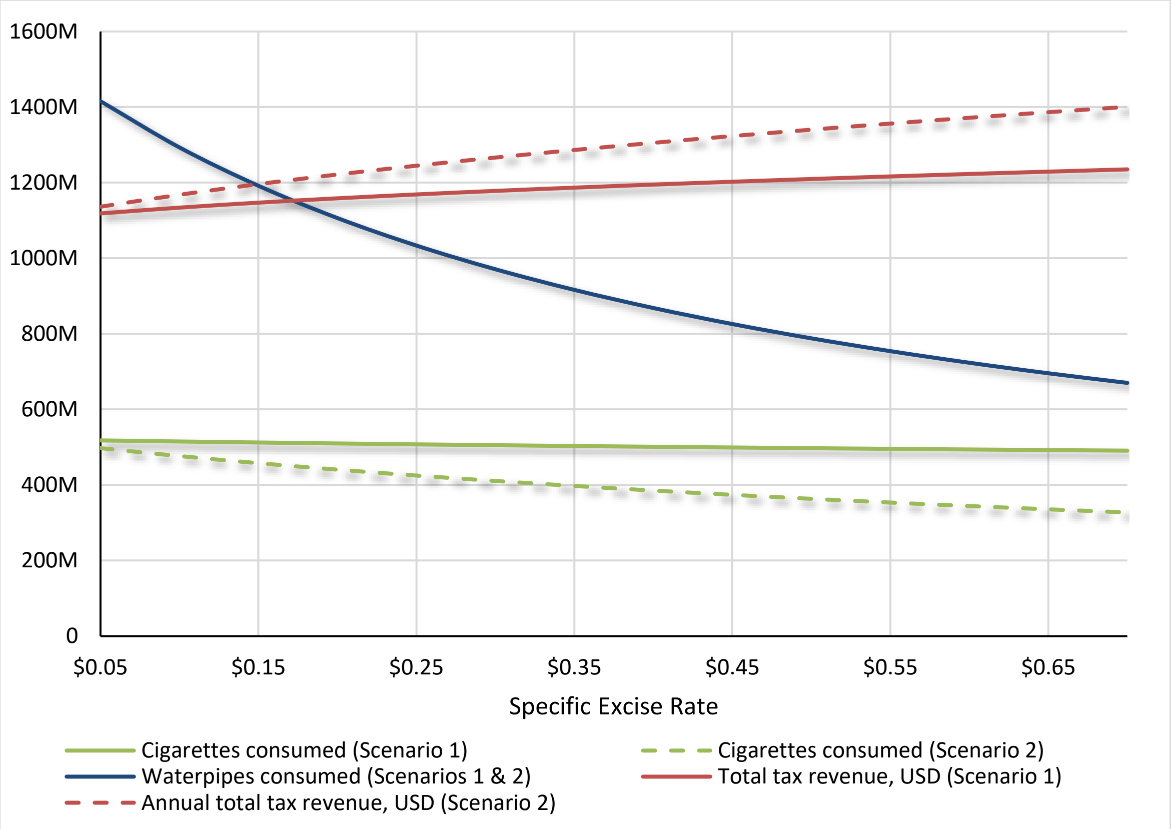

Conclusion

Increasing the specific excise tax on cigarettes and waterpipe tobacco is an effective way to increase government revenue in Lebanon. We recommend that any increase in specific tax is

applied equally to cigarettes and waterpipe tobacco in order to minimise substitution between products, and that specific taxes are extended to domestically produced cigarettes.

FREQUENTLY ASKED QUESTIONS

What data were used to inform the model?

- Tax structure: World Health Organization report on the global tobacco epidemic (2021)[1]
- Cost, insurance, and freight price: UN Comtrade
- Tobacco market shares, consumer demand, tobacco prices, and tobacco price elasticities: Research conducted by AUB[1]

Which tax rates and tax bases were used to inform the model?

Tax	Tax rate: Cigarettes	Tax rate: Waterpipe	Tax base
Import duty	6.2%	35.5%	CIF price
Specific excise duty	$0.17	$0.03	Per cigarette or 20g of waterpipe
Ad valorem excise duty	126%	157%	CIF price and import duty
Value added tax	8.4%	8.6%	Retail price

What were the model assumptions?

Since we did not have accurate or complete tobacco sales and taxation data for all cigarette and waterpipe tobacco products in Lebanon, we made the following assumptions that were informed by research conducted by AUB[2]:

- The cigarette market is divided into three shares: imported cigarettes, premium domestic cigarettes, and discount domestic cigarettes.
- The waterpipe market is divided into four shares: discount café, premium café, discount home, and premium home use.
- The entire waterpipe market is imported while 16.0% of the cigarette market is imported.
- Waterpipe consumption is 2-3x more likely to reduce ("more elastic") than cigarettes for any given specific tax.
- No change in illicit tobacco use following a tax increase.
- That the entire cost of the tax, and no more, was passed onto the consumer (i.e. no industry under- or over-shifting)
- That consumers either cut down or quit smoking their tobacco product in response to taxation and do not switch to other products (e.g. e-cigarettes, smokeless tobacco, cigar, pipe).

How does the model work?

The model is based on the widely-used TETSim model to calculate the effects of a tax change on the consumer demand and revenue raised of each market share. What is presented in Figure 1 and Table 1 are the market-weighted averages across all tobacco products.

What are the limitations of the model?

[1] https://www.who.int/publications/i/item/9789240032095
[2] See https://pubmed.ncbi.nlm.nih.gov/35246419/ and https://pubmed.ncbi.nlm.nih.gov/34193608/

The model does not make projections into the future – it simply reports an annual snapshot should the specific tax scenarios be implemented today. It is also unable to provide complete certainty on the behaviour of the tobacco industry and the smoking behaviour of consumers in response to much larger tax increase. However, the model has been tested for accuracy under changing assumptions and performed very well in producing consistent results.

TABLES

Table 1. The impact of various specific tax scenarios on the number of annual cigarettes and waterpipes consumed in Lebanon and on annual government revenue from import duty, excise duty, and value added tax

	Scenario 1: Specific tax applied to imported cigarettes and waterpipe only			Scenario 2: Specific tax applied to imported and domestic cigarettes and waterpipe		
Specific tax	Cigarettes consumed	Waterpipes consumed	Annual total tax revenue, USD	Cigarettes consumed	Waterpipes consumed	Annual total tax revenue, USD
$0.05	518,011,924	1,415,590,125	1,118,628,024	497,592,098	1,415,590,125	1,136,398,903
$0.10	515,172,409	1,293,272,857	1,134,010,292	476,645,432	1,293,272,857	1,168,055,978
$0.15	512,499,511	1,191,781,867	1,147,139,199	457,779,250	1,191,781,867	1,196,197,462
$0.20	509,979,290	1,106,370,341	1,158,633,780	440,670,383	1,106,370,341	1,221,626,240
$0.25	507,599,304	1,033,587,760	1,168,913,299	425,062,508	1,033,587,760	1,244,908,574
$0.30	505,348,411	970,874,997	1,178,266,542	410,749,258	970,874,997	1,266,452,515
$0.35	503,216,607	916,300,109	1,186,895,374	397,562,286	916,300,109	1,286,557,956
$0.40	501,194,886	868,381,470	1,194,942,779	385,362,648	868,381,470	1,305,449,378
$0.45	499,275,116	825,966,664	1,202,511,254	374,034,466	825,966,664	1,323,297,734
$0.50	497,449,940	788,147,879	1,209,675,115	363,480,194	788,147,879	1,340,235,365
$0.55	495,712,684	754,201,701	1,216,488,836	353,617,021	754,201,701	1,356,366,341

$0.60	494,057,281	723,545,585	1,222,992,792	344,374,106	723,545,585	1,371,773,753
$0.65	492,478,202	695,705,900	1,229,217,262	335,690,424	695,705,900	1,386,524,914
$0.70	490,970,402	670,294,156	1,235,185,243	327,513,073	670,294,156	1,400,675,138

Document 2

Policy Brief:
Enforcing Waterpipe Tobacco Tax Increase in Jordan to Improve Public Health Outcomes

Authors:

Professor Nihaya A. Al-sheyab

Dean of the Faculty of Applied Medical Sciences at Jordan University of Science and Technology

Email: nasheyab@just.edu.jo

Professor Mohammad S. Alyahya

Faculty Medicine at Jordan University of Science and Technology

Email: msalyahya@just.edu.jo

Professor Yousef S. Khader

Faculty Medicine at Jordan University of Science and Technology

Email: yskhader@just.edu.jo

Key messages

What is the Problem?

About two thirds of adult males in Jordan smoke tobacco (Drope J, et al., 2018; World Health Organization, 2019), and this rate is considered the highest in the Eastern Mediterranean Region

and the second highest worldwide. The most recent study showed that the crude prevalence rates in Jordan for current cigarette smoking, waterpipe smoking, and dual current smoking were 32.0% (29.9% to 34.1%), 11.0% (9.6% to 12.4%) and 2.8% (2.1% to 3.6%), respectively (Nakkash, et al., 2022). Apart from the high rates of cigarette smoking, Jordan has been experiencing significant increase in waterpipe smoking (Jawad, Lee, et al., 2016), especially among females (Jawad, Abdulrahim, & Daouk, 2016) and youth (Jawad & Roderick, 2017; Ministry of Health, 2014a).

What do we know about two elements of an approach to addressing the problem?

Element 1> ***Increase tobacco taxation and strictly enforce it throughout the country.***

- Rising the price of cigarettes through increased taxes has also been successful in reducing smoking among youth, young adults, and persons of low socioeconomic status.
- Younger people seem to be more price sensitive as younger smokers tend to earn lower salaries and are less reliant on tobacco, both of which would appear to make them more price responsive.
- About 40% tax-induced cigarette price rise would decrease smoking prevalence from 21% in 2004 to 15.2% in 2025 with huge improvements in cumulative life years and quality adjusted life years over two decades.
- A 10% rise in taxation on waterpipe tobacco smoking would decrease the demand on waterpipe tobacco smoking by 14.5%.

- Both media campaign and tax increase of $1 per a cigarette pack will lessen overall smoking prevalence as well as associated mortality.
- The total price elasticity of cigarette and waterpipe demand in Jordan was estimated to be moderately elastic (-0.6).
- The cross-price elasticity between cigarettes and waterpipe tobacco is near zero, suggesting that the two products are not considered to be close substitutes by consumers and therefore tax policies are expected to be effective in Jordan.
- Lower availability of less expensive smuggled cigarettes is projected to inspire some smokers to quit and may rise tax revenues from those who do not quit smoking.

Element 2> *Implement smoking cessation interventions in order to support clients who will stop smoking after tax rise.*

- Once people who smoke can no longer afford cigarettes or waterpipe smoking, they have an option to quit, thus need professional smoking cessation counselling.
- For a treatment cohort of 527,118 on Nicotine Replacement Therapy, Jordanian male smokers who intended to quit, 103,970 life years are expected to be gained using the varenicline regimen, while 64,030 life years are expected to be gained using the Nicotine replacement therapy (NRT) regimen as compared to the control group of life years.
- The cost per life year gained is estimated at $1696 USD for varenicline and $1890 USD for NRT.
- NRT, brief advice, and behavioral counseling can be effective in helping smoking cessation in low- and middle-income countries.
- Higher elasticity is likely to be obtained by introducing/increasing specific excise taxes and concerted efforts to implement non-price (tax) related WHO FCTC measures, such as cessation programs, bans on advertisement, and improving public awareness.

Implementation considerations

Several implementation considerations are required at multiple levels (citizen, organization, and systems) to effectively develop and enforce a policy to increase tax on cigarette and waterpipe

smoking products in Jordan. Barriers to implementing such policy can be overcome at several levels.

Executive Summary

Smoking is the leading cause of preventable death as well as a main risk factor for cancer, cardiovascular and pulmonary diseases. In Jordan, the tobacco epidemic is on constant increase and defined as a public health emergency (Al-Tammemi, 2021). About two thirds of adult males in Jordan smoke tobacco (Drope J, et al., 2018; World Health Organization, 2019), and this rate

is considered the highest in the Eastern Mediterranean Region and the second highest worldwide. Also, about half of Jordanian youth aged 13-15 years old reported tobacco smoking at least once (Ministry of Health, 2014a), which is again the highest in the Eastern Mediterranean region among youth (Arrazola et al., 2017). Second-hand smokers are also exposed to tobacco smoking with 68% of adults (Ministry of Health, 2007) and 62% of youth are considered second hand smokers (Ministry of Health, 2014a). The most recent study showed that the crude prevalence rates in Jordan for current cigarette smoking, waterpipe smoking, and dual current smoking were 32.0% (29.9% to 34.1%), 11.0% (9.6% to 12.4%) and 2.8% (2.1% to 3.6%), respectively (Nakkash, et al., 2022).

The most common types of tobacco products in Jordan are cigarettes and waterpipe (Jawad, Lee, & Millett, 2016; Ministry of Health, 2007). Apart from the high rates of cigarette smoking, Jordan has been experiencing significant increase in waterpipe smoking (Jawad, Lee, et al., 2016), especially among females (Jawad, Abdulrahim, & Daouk, 2016) and youth (Jawad & Roderick, 2017; Ministry of Health, 2014a), as it is more culturally acceptable than smoking cigarettes (Jawad & Roderick, 2017). This is alarming given the fact that a 45 minute waterpipe session (Waziry et al., 2017) is almost equal to smoking an average of 60-200 cigarettes (Ali & Jawad, 2017). Different factors have contributed to the rise in waterpipe smoking in Jordan as well as the whole region, including the introduction of flavoured tobacco, the perception of reduced harm and addiction as compared to cigarette smoking, and inadequate policies and regulations addressing waterpipe smoking (Akl, et al., 2015).

Among established tobacco smokers, cessation leads to a substantial reduction in risk, with the largest reductions among individuals who stop smoking before age 40 years (National Center for Chronic Disease Prevention and Health Promotion, 2014). In 2004, Jordan ratified and signed the Framework Convention on Tobacco Control of the World Health Organization (United Nations Treaty Collection, 2019). Following this approval, Jordan developed the first Tobacco Control Law as a part of Public Health Law 47 (Ministry of Health, 2008), which was endorsed in 2008 with additional amendments in 2017. Article 5.3 of the WHO FCTC requests establishment of formal mechanisms to protect against the negative influence of tobacco industry on tobacco control policies (World Health Organization, 2003).

Although Jordan was among the first countries in the WHO Eastern Mediterranean Region to ratify the WHO FCTC (United Nations Treaty Collection, 2019), there has been constant interference from the tobacco industry and resistance from several stakeholders, including the hospitality sector, against the implementation of tobacco control laws (World Health Organization, 2013). Moreover, tobacco companies aim to improve their image with the media, public, and the government, but at the same time delaying and discouraging effective tobacco control policies and interventions (World Health Organization, 2013). One law that was developed but yet enforced in Jordan is higher taxes on tobacco products, however, waterpipe tobacco products are still not taxed in Jordan. Although tobacco tax raise is an effective way to increase revenue and lower smoking prevalence, there are many ways they can be undervalued by tobacco industry and people who smoke tobacco. These smokers are likely to react to higher tobacco taxes muting the impact of raises. The following are some of smokers' reactions to tax raise:

- Converting into cheaper and less taxed brands
- Using more price-reducing promotions (coupons and special offers) offered by tobacco companies.
- Purchasing larger amounts of tobacco for multipack discounts
- Buying black-market cigarettes *(Goldman, 2016)*.

Two elements were proposed in this policy brief based on local, national, and international evidence (both single studies and systematic reviews) in order to decrease tobacco smoking (cigarettes and waterpipe) in Jordan. The first element is increasing tobacco taxation and strictly enforce it throughout the country. The second element is implementing smoking cessation interventions in order to support clients who will stop smoking after tax rise.

Several implementation considerations are required at multiple levels (citizen, organization, and systems) to effectively develop and enforce a policy to increase tax on cigarette and waterpipe smoking products in Jordan. Barriers to implementing such policy can be overcome at several levels.

Evidence has shown that increasing tobacco taxation can improve health outcomes. Increasing the retail price of tobacco as a result of tax rise can decrease the demand for tobacco. In low-and middle-income countries, a 10% increase in tobacco prices will approximately lead to about 4%-8% decrease in smoking. This reduction will have positive health effects for both active and passive smokers and decrease morbidity and mortality from tobacco-related diseases. Using panel data sourced from the World Health Organization (WHO) and the World Bank databases on 24 African countries for the period 2010 to 2016, a percentage increase in tobacco price will reduce the smoking prevalence by between 0.11 to 0.14%, whereas a percentage increase in tobacco tax will reduce smoking prevalence by between 0.25 to 0.36% (Immurana, Boachie, & Iddrisu, 2021).

In order to achieve the ultimate purpose of tobacco tax increase, which is reducing tobacco smoking and at the same time increasing the government's revenue, the following strategies need to be employed (World Bank Group, 2019):

- Excise rates for cigarettes, waterpipe tobacco, and other tobacco products should be unified for all kinds of cigarettes and waterpipe content (ma'assel) and annually increased by at least 20% to ensure tobacco affordability reduction and thus reducing prevalence in line with FCTC provisions (World Bank Group,
-

  2019). Minimizing the price gaps between various tobacco products and brands is needed to limit the ability of smokers to substitute between products.
- Tobacco use surveillance and monitoring should be further strengthened in Jordan, including a regular collection and public presentation of information on sales of cigarettes and other tobacco products, their prices as well as other economic indicators to support more specific estimates of the outcomes of ongoing and future tobacco control actions (World Bank Group, 2019).
- Effective policies to counteract tobacco smuggling and other kinds of illicit tobacco sales should be implemented in line with the provisions of the FCTC Protocol to Eliminate Illicit Trade in Tobacco Products, which is recommended to be ratified (World Bank Group, 2019).

Full Policy Brief:

Enforcing Waterpipe Tobacco Tax Increase in Jordan can Improve Public Health Outcomes

The Problem

Jordan has one of the highest rates of tobacco smoking in the region and globally. According to Jordan Population and Family Health Survey in 2019, about 50% of men and 12% of women are daily smokers (Jordan Population and Family Health Survey, 2019). The most recent national study showed that the crude prevalence rates of current cigarette smoking, waterpipe smoking, and dual current smoking were 32.0% (29.9% to 34.1%), 11.0% (9.6% to 12.4%) and 2.8% (2.1% to 3.6%), respectively (Nakkash, et al., 2022), and the age-standardised prevalence rates of current cigarette and waterpipe smoking in Jordan were 25.5% and 12.0% respectively (Nakkash, et al., 2022). The prevalence rates of waterpipe smoking among men and women in Jordan were 13.4% and 7.8%, respectively (Nakkash et al., 2022). Waterpipe tobacco smoking has spread rapidly in Jordan over the past three decades, especially among young people (Hamadeh RR et al., 2020). These high rates should be taken into considerations to fortify existing policies for tobacco control.

Size of the Problem

Approximately 80% of the world's one billion smokers reside in low- and middle-income countries (LMICs) (Jha &Peto, 2014). It is expected that, if this trend remains, 70% of the estimated 10 million smoking-related deaths will occur in LMICs by the year 2030 (Jha &Peto, 2014). Globally, tobacco smoking results in 500,000 premature deaths every year (Fishman, et al., 2005). The majority of people start to smoke tobacco before the age of 21 years old, hence

efforts and policies should focus on targeting preventing or reducing youth smoking (Fishman, et al., 2005).

Tobacco smoking leads to premature death of about 6 million people globally and 96,000 in the UK alone annually (Action on Smoking and Health, 2014; WHO, 2013). According to 2015

GBD study (Reitsma, Fullman, Salama, Abajobir, et al., 2017), 11.55% of global deaths (6·4 million [95% UI 5·7–7·0 million]) were attributable to smoking worldwide. Smoking was ranked among the five leading risk factors by DALYs in 109 countries and territories in 2015, rising from 88 geographies in 1990 (Reitsma, Fullman, Salama, Abajobir, et al., 2017).

A national recent survey reported that 8 out of 10 Jordanian men were regular smokers consuming about 23 cigarettes daily (Safi & Al-tahat, 2020). Another study showed that the prevalence of past-30-day waterpipe smoking among university students was 67.7% (Salloum, et al., 2019). Alarmingly, the World Population Review ranked Jordan sixth globally with an overall smoking prevalence of 40.4% (10.7% among women and 70.2% among men) (World Population Review, 2021). A recent national study of the association of smoking with direct medical expenditure of chronic diseases (Alefan, et al., 2019) reported that the annual total direct medical expenditures were 1,054,681 JD for non-smokers, 466,292 JD for former smokers and 374224 JD for smokers. The same study found that the median total annual direct medical expenditures per patient were 845 JD for smokers, 911 JD for former smokers and 714 JD for non-smokers. Former smokers had the highest total annual expenditure with 2467 JD per patient (Alefan, et al., 2019). Smokers and former smokers were associated with both the highest inpatient-related and outpatient-related services expenditures (568 JD and 480 JD, respectively) and the highest inpatient expenditures (214 JD and 191 JD, respectively).

Nonetheless, Jordan had low scores in implementing the MPOWER measures and ranked the 13th in the EMR (Heydari et al.,2017). Consequently, the absence of effective and enforced tobacco control measures can increase the number and intensity of tobacco smoking consequences. Different factors have contributed to the rise in waterpipe smoking in Jordan as well as the whole region, including the introduction of flavoured tobacco, the perception of reduced harm and addiction as compared to cigarette smoking, and inadequate policies and regulations addressing waterpipe smoking (Akl, et al., 2015). Hence, high quality measures and interventions are needed especially those related to waterpipe smoking reduction and cessation (Jawad, et al., 2016). In the meantime, fiscal policies on cigarette smoking should be also consider waterpipe smoking (Jawad, et al., 2016) as waterpipe tobacco products are still not

taxed in Jordan. Table 1 shows the tobacco expenditure and consumption in Jordan elicited from a household survey data (World Bank, 2019**).**

Underlying Factors

The issue of tobacco smoking in Jordan is complicated by several local underpinning factors, which make efforts of anti-tobacco mission more challenging.

Governance

One reason of the issue of tobacco smoking in Jordan could be the high influence level and interloping from tobacco companies in tobacco policymaking (Global Tobacco Index, 2020). Other reasons include the lack of transparency in disclosure of interactions between tobacco industry and the Jordanian government and the absence of regulations that stop government officials and relatives from holding positions in tobacco industry (Global Tobacco Index, 2020). Moreover, the Jordanian government has not been taking the issue of waterpipe smoking as serious as cigarette smoking. Despite the alarmingly high popularity of waterpipe smoking in Jordan among all population segments as well as the accumulated evidence of negative effects of waterpipe tobacco on health (Jawad, et al., 2016), health warnings were issued for cigarette packs only (World, Bank, 2019). Several complementary policies will strengthen anti-waterpipe smoking in the country, such as further restriction on smoking in public places, such as restaurants and cafes.

Financing

Cigarette prices and associated taxes in Jordan was compared to that in other neighboring

countries. The net-of-tax part of the price in Jordan was very low as compared to some neighboring countries such as Lebanon and Saudi Arabia (WHO, 2017a). Current waterpipe tobacco taxation is 21% of the retail price in Jordan compared to 42% in Palestine. Regardless, the most popular cigarette brands were the cheapest in Jordan (WHO, 2017b) and the fake brands cigarettes are spreading in Jordan (WHO, 2013) on top of high affordability (World bank, 2019). In 2017 and 2018, tobacco price increased significantly in Jordan, and tobacco affordability reduction was high enough, both of which could have led to a decrease in tobacco consumption

in the country (World Bank, 2019). The total price elasticity of cigarette demand in Jordan was moderately elastic and estimated to be -0.6 (Sweis, et al., 2014). Also, the cigarette excise taxes in Jordan is considered one of the main revenues in the government. Nonetheless, the price elasticity estimates suggest that significant increases in tobacco taxes are likely to be effective in reducing tobacco smoking in Jordan (World Bank, 2019).

Annex 2 demonstrates tobacco affordability in Jordan. To meet 75% targets, Jordan would need to increase its specific tax from $0.18 to $7.03 per 20g of waterpipe tobacco, resulting in an average price increase from $2.05 to $11.19 and nearly 16 million fewer waterpipe sessions (70% reduction) while increasing tax revenue by $43 million (455% increase) annually.

Although most data are available for cigarette smoking, Shepard et al. (2017) suggested that the supplemental sales tax applies to all tobacco products including waterpipe smoking. As a sales tax, it can be applied to the full retail price, including waterpipe smoking in cafes (Shepard, et al., 2017). Several complementary policies will strengthen these gains. Similar to many products which their maximum prices are regulated in the country, tobacco smoking needs to be included in these regulations as well. If this applies to tobacco products, the government could promise to consider requests for increasing the pre-tax price of cigarettes to allow tobacco companies to maintain their profits despite lower sales. All of these policies support Jordan's Tobacco Control Alliance and its participation in the WHO Framework Convention for Tobacco Control (Shepard et al., 2017). Similarly, taxing the entire spectrum of tobacco products can reduce their use, prevent switching to less expensive products, and create substantial tax revenues (WHO, 2010).

Shepard et al. (2017) revenue projections were based on legal cigarette sales. The rate of the proposed sales tax is sufficiently modest that it should not aggravate the amount of illegal tobacco use. Additionally, if some of the increased revenues were channeled into more strong enforcement against smuggling, the action could foster a good circle. Lower availability of less expensive smuggled cigarettes is projected to inspire some smokers to quit and can rise tax revenues from those who do not quit smoking (Shepard, et al., 2017). Indeed, higher elasticity is likely to be obtained by introducing/increasing specific excise taxes and concerted efforts to

implement non-price (tax) related WHO FCTC measures, such as cessation programs, bans on advertisement, and improving public awareness (Ho, et al., 2018).

Jordan has increased cigarette taxes over the years, including early in 2017, but has not substantially raised taxes on other forms of tobacco use
These findings serve as a strong evidence base for developing and implementing fiscal policies for tobacco control in the Eastern Mediterranean region that address cigarettes and waterpipe tobacco products (Chalak et al., 2021). Using panel data sourced from the World Health

Organization (WHO) and the World Bank databases on 24 African countries for the period 2010 to 2016, a percentage increase in tobacco price will reduce the smoking prevalence by between 0.11 to 0.14%, whereas a percentage increase in tobacco tax will reduce smoking prevalence by between 0.25 to 0.36% (Immurana, Boachie, & Iddrisu, 2021). Table 3 illustrates the tax burden for cigarettes in Jordan for a pack of 20 cigarettes, in fils.

Delivery
Several laws address tobacco control legislation in Jordan, such as the Public Health Law 47/2008, which addresses ban on tobacco advertising and promotion, smoke-free places, and display of tobacco products (Ministry of Health, 2008). Other regulations deal with pictorial health warnings, waterpipe content, as well as packaging and labeling (Jordan Standards and Metrology Organization, 2000; Jordan Standards and Metrology Organization, 2012a; Jordan Standards and Metrology Organization, 2014a). The Juvenile Conduct Law (2006) prohibits selling tobacco to minors (Ministry of Interior, 2006, 2008). Despite all these laws, according to the Jordanian Ministry of Health (MOH), there are several barriers in implementing these laws in Jordan resulting in constant violations of the law. For example, a local report by the MOH claimed that smoking in public places, selling tobacco to minors (Ministry of Health, 2014a), and serving waterpipe in restaurants and cafes make these violations acceptable diminishing all laws.

Another barrier to enforcing laws is the tobacco industry in Jordan, which has been opposing tobacco control efforts and inhibiting the execution and implementation of related legislations (World Health Organization, 2015). Not only that, the tobacco industry has been constantly

making efforts through direct and indirect lobbying such as participation with Jordan Standards and Metrology Organization to influence ministers and parliamentarians to push for more tobacco friendly policies (Jordan Restaurant Association, 2017). The tobacco industry utilizes top stakeholders and policymakers to exert pressure at all levels of the government to prevent FCTC full implementation and enforcement. Consequently, an increase in tobacco use, especially waterpipe smoking, was reported recently due to the lobbying with the Jordan Restaurant Association (Ministry of Health, 2011a, 2011b, 2018). Similarly, waterpipe smoking is still provided inside cafes and restaurants impeding the implementation of the smoke-free law in public places as more than 700 places serving waterpipe are licensed in Jordan (Jordan Restaurant Association, 2017) with much higher number of places that do not have a license.

In 2013, the tobacco industry responded to tax raise on tobacco products by reducing the price of cigarettes by 25% (World Health Organization, 2015) claiming that lowering the price could reduce cigarette smuggling, but the truth was to keep cigarettes affordable (Ministry of Health; World Health Organization, 2015). The tobacco companies in Jordan have been making efforts to improve their image with the media, community, and government alike (World Health Organization, 2013). These efforts included donating funds for educational programs and community-based initiatives (World Health Organization, 2013). According to the Jordanian Department of Statistics, tobacco industry spent 273,000 JOD in sponsorship and donations in 2015 (Department of Statistics, 2016).

Ultimately, the tobacco industry has been succeeding in delaying the implementations of several tobacco laws in Jordan. For example, the tobacco companies in Jordan were granted six months before executing the new pictorial warnings on cigarette packs (Jordan Standards and Metrology Organization, 2014b). Another example is that the government permitted tobacco companies to launch designated smoking areas in 2010 as a short transition phase towards the full implementation of the smoke-free public places law (Prime Ministry of Jordan, 2017), however, this still ongoing after more than one decade and has created confusion and hence resistance of smoke-free implementation. All these unjustified delays in enforcing the laws by the government are partly due to conflict of interest as some of the house of representative members and

ministers have shares in tobacco companies or own restaurants that serve waterpipe (Maraq.com, 2014).

Elements to address the problem

Element 1: ***Increase tobacco taxation and strictly enforce it throughout the country.***

One study reported that the increase in tobacco price through taxation is considered vital for driving success in tobacco smoking reduction and/or prevention (Bader, et al., 2011). This study also indicated that rising the price of cigarettes through increased taxes has also been successful in adolescent and youth tobacco smoking and people with low socioeconomic status (Bader, et al., 2011). The World Bank, in a recent report, reported that increasing prices of cigarettes decreases affordability taking into consideration that the inflation rate is growing (World Bank, 2019). Similarly, a national study reported that the total price elasticity of cigarette demand in Jordan was estimated to be -0.6 (Sweis & Chaloupka, 2014). The price elasticity estimates, according to a recent World Bank report, suggest that significant increases in tobacco taxes are likely to be effective in reducing tobacco smoking in Jordan (World Bank, 2019).

According to the UNICEF national report in 2017, revenue projections were based on legal cigarette sales (Shepard et al., 2017). The rate of the proposed sales tax is sufficiently modest that it should not aggravate the amount of illegal tobacco use. Additionally, this report suggests that if some of the increased revenues were channeled into more strong enforcement against smuggling, the action could foster a good circle. Lower availability of less expensive smuggled cigarettes is projected to inspire some smokers to quit and can rise tax revenues from those who do not quit smoking (Shepard, et al., 2017).

Yet, a study conducted in Lebanon reported that a 10% rise in taxation on waterpipe tobacco smoking would decrease the demand on waterpipe tobacco smoking by 14.5% (Jawad, et al.,

2016). A systematic review found that both media campaign and tax increase of $1 per a cigarette pack will lessen overall smoking prevalence as well as associated mortality (Fishman, et al., 2005). Another systematic review found that 40% tax-induced cigarette price rise would decrease smoking prevalence from 21% in 2004 to 15.2% in 2025 with huge improvements in cumulative life years and quality adjusted life years over two decades (Ahmad & Franz, 2008).

Larger tax rise can maximize these benefits and decrease smoking prevalence (Ahmad and Franz, 2008; Higashi, et al., 2011). A recent volumetric choice experiment in three EMRs including Jordan found that the demand for premium waterpipe tobacco was highly elastic in Lebanon (−1.9), moderately elastic in Jordan (−0.6) and inelastic in Palestine (0.2) (Chalak, et al., 2021). This study also reported that the cross-price elasticity between waterpipe tobacco and cigarettes was near zero, suggesting that cigarettes and waterpipe tobacco are not considered to be close substitutes by smokers. Hence, governments must increase taxes on cigarettes and waterpipe tobacco separately, and that tax-increase policies will be effective due to the fact that tobacco smokers are less likely to substitute cigarettes with waterpipe tobacco (Chalak, et al., 2021). In 2017, the Prime Ministry in Jordan enforced a new law that preventing issuing new licenses to cafes and restaurants in an attempt to reduce waterpipe smoking (Prime Ministry of Jordan, 2017).

Although most data are available for cigarette smoking, Shepard, et al (2017) in the UNICEF report suggest that the supplemental sales tax applies to all tobacco products including waterpipe smoking. They explained that as a sales tax, it can be applied to the full retail price, including waterpipe smoking in cafes (Shepard, et al., 2017). Several complementary policies will strengthen these gains, such as further restriction on smoking in public places including restaurants. Also, in Jordan, the maximum prices of many products are regulated. If this applies to tobacco products, the government could promise to consider requests for increasing the pre-tax price of cigarettes to allow tobacco companies to maintain their profits despite lower sales. All of these policies, according to the UNICEF report, support Jordan’s Tobacco Control Alliance and its participation in the WHO Framework Convention for Tobacco Control (Shepard et al., 2017).

In particular, there are several benefits of increasing taxation of tobacco and enforcing it throughout the country. First, a single study found that increasing taxes on tobacco products could benefit youth the most, as the majority of people start to smoke tobacco before the age of 21 years old (Fishman, et al., 2005). Importantly, another study found that younger people seem to be more price sensitive as younger smokers tend to earn lower salaries and are less reliant on tobacco, both of which would appear to make them more price responsive (Sweis & Chaloupka, 2014). Nonetheless, a study found that price elasticity estimates show that significant increases in taxes that raise prices of cigarette can be effective in decreasing cigarette smoking and associated consequences (Sweis & Chaloupka, 2014).

Moreover, government expenditure on smoking-related diseases could decrease if the tax increase policy becomes successful. A systematic review found that smoking co-morbidities are responsible for 1.5 – 6.8 % of the national health system expenditures and 0.22-0.88% of GDP of a country (Rezaei et al., 2016). However, there might be some harms of implementing and enforcing tobacco increase taxes. ‘Hard-to-quit’ smokers and people with lower socio-economic status could still prioritize buying smoking packs over other necessary household necessities, which might impact the quality of life of family members. A single local study (Toukan, 2016) reported that the Average poorest male cigarette smokers with a monthly income of 100-250 JD spend 25 times more on cigarettes than on health, 10 times more on cigarettes than on education, 1.5 times more on cigarettes than on food, and 2.5 times more on cigarettes than on housing.

In regards to cost and cost effectiveness, a report about price elasticity estimates by the WHO suggest that increase in the price of tobacco products through tax increase can reduce smoking rates in Jordan (World Bank, 2019; WHO, 2019; Sweis & Chaloupka, 2014), thus, government expenditure on smoking co-morbidities will decrease. Another report by the WHO found that taxing the entire spectrum of tobacco products can reduce their use, prevent switching to less expensive products, and create substantial tax revenues (WHO, 2010).

In regards to uncertainty regarding benefits and potential harms, no robust local data about the benefits and/or potential harm. Available evidence are only from single studies and WHO reports, which suggest that increased cigarette taxes without a corresponding increase in taxes on

other tobacco products can encourage a substitution, which may result in lost revenue, possibly with no reduction in consumption (WHO, 2010). Additionally, Black market, illicit cigarette trade, fake cigarettes, E-cigarettes, or other types of cheap drugs could be a satisfactory, yet risky alternative. About 46% of all cigarettes sold in Jordan in 2012 are illicit smuggled cigarettes (El-El-Khushman, et al., 2008). Importantly, WHO reports found that people might switch to cheaper brands as it was documented that the most popular cigarette brands were the cheapest in Jordan (WHO, 2017; 2019).

Stakeholders also had views in this matter. Princess Dina Mered [president of the Union for international cancer control] said: "we need to impose more taxes on the price of cigarettes and protect our children from tobacco companies (Shafey, et al., 2005). Also, Dr. Abeer Mwaswas [Director of the department of MOH awareness and health communication] said: "lots of measures depend on other ministries such as trade, justice, or finance" (Tobacco control in Jordan, 2019). Importantly, Philip Morris International said: "we were forced to reduce the price of cigarettes in Jordan to address the serious problem of illicit smuggled cigarettes".

Element 1

**Category**	**Selected Option/Element:** ***increase tobacco taxation and strictly enforce it throughout the country.***
**Benefits**	• A systematic review found that government expenditure on smoking-related diseases could decrease if the tax increase policy becomes successful (Rezaei et al., 2016).<br>• A single study from 24 African countires found that a percentage increase in tobacco price will reduce the smoking prevalence by between 0.11 to 0.14%, whereas a percentage increase in tobacco tax will reduce smoking prevalence by between 0.25 to 0.36% (Immurana, Boachie, & Iddrisu, 2021)

Category	Selected Option/Element:<br>***increase tobacco taxation and strictly enforce it throughout the country.***
	• Increasing taxes on tobacco products could benefit youth the most, as the majority of people start to smoke tobacco before the age of 21 years old (Fishman, et al., 2005).<br>• A single study found that younger people seem to be more price sensitive as younger smokers tend to earn lower salaries and are less reliant on tobacco, both of which would appear to make them more price responsive (Sweis & Chaloupka, 2014). Nonetheless, this study found that price elasticity estimates show that significant increases in taxes that raise prices of cigarette can be effective in decreasing cigarette smoking and associated consequences (Sweis & Chaloupka, 2014).<br>• A systematic review concludes that government expenditure on smoking-related diseases could decrease if the tax increase policy becomes successful. This systematic review found that smoking co-morbidities are responsible for 1.5 – 6.8 % of the national health system expenditures and 0.22-0.88% of GDP of a country (Rezaei1, Akbari, Mohammad Arab, Reza Majdzadeh, Asghar Mohammad & Poorasl, 2016).
**Potential harms**	•
**Cost**<br>and/ or cost effectiveness in relation to the status quo	• Several single studies and WHO reports found that the price elasticity estimates suggest that increase in the price of tobacco products through tax increase can reduce smoking rates in Jordan (Nakkash, et al., 2022; WHB, 2019; WHO, 2019; Sweis & Chaloupka, 2014), thus, government expenditure on smoking co-morbidities will

**Category**	**Selected Option/Element:** ***increase tobacco taxation and strictly enforce it throughout the country.***
	decrease. Also, at the individual level, smokers who are expected to quit after the price increase can save money and utilize it in more important aspects.<br>• The WHO report showed that the net-of-tax part of the price in Jordan was very low as compared to some neighboring countries (WHO, 2017a).<br>• Current waterpipe tobacco taxation is 21% of the retail price in Jordan compared to 42% in Palestine.<br>• The WHO reports and World Bank indicators showed that the most popular cigarette brands were the cheapest in Jordan (WHO, 2017b) and the fake brands cigarettes are spreading in Jordan (WHO, 2013) on top of high affordability (World bank, 2019).<br>• The World Bank indicators In 2017 and 2018 demonstrated that tobacco price increased significantly in Jordan, and tobacco affordability reduction was high enough, both of which could have led to a decrease in tobacco consumption in the country (World Bank, 2019).
**Uncertainty** regarding benefits and potential harms	• Uncertainty regarding benefits and potential harms were not reported clearly by the evidence

Category	Selected Option/Element:<br>***increase tobacco taxation and strictly enforce it throughout the country.***
**Stakeholders' views**	• According to Tobacco Atlas, Princess Dina Mered [president of the Union for international cancer control] said: "we need to impose more taxes on the price of cigarettes and protect our children from tobacco companies (Shafey, et al., 2005).<br>• Philip Morris International said: "we were forced to reduce the price of cigarettes in Jordan to address the serious problem of illicit smuggled cigarettes".
**Equity considerations**	• Equity considerations was not reported by the evidence

Element 2: ***Implement smoking cessation interventions in order to support clients who will stop smoking after tax rise.***

This initiative is promising in increasing attempts to quit smoking, treatment use, and rates of successful quitting (Fiore, et al., 2008). This option is related to the first element of tax increase. Once people who smoke can no longer afford cigarettes or waterpipe smoking, they have an option to quit, thus need professional smoking cessation counselling along with adequate related resources such as medications- to make the process of quitting more successful and avoid relapse. Similarly, medical insurance coverages that reimburse tobacco cessation interventions may increase the possibility that physicians will intervene with smokers and provide comprehensive counseling (McAfee, et al., 2015). Similarly, the National Center for Chronic Disease Prevention and Health Promotion office reported that among established tobacco smokers, cessation leads to a substantial reduction in risk, with the largest reductions among individuals who stop smoking before age 40 years (National Center for Chronic Disease Prevention and Health Promotion, 2014).

Moreover, a local policy analysis showed that for a treatment cohort of 527,118 Jordanian male smokers who intended to quit, 103,970 life years were gained using the varenicline regimen, while 64,030 life years were gained using the NRT regimen as compared to the control group of life years. The cost per life year gained was $1696 USD for varenicline and $1890 USD for NRT (Mada'een et al., 2020).

Furthermore, NRT, brief advice, and behavioral counseling can be effective in helping smoking cessation in low- and middle-income countries (Akanbi, et al, 2019). Yet, there is still limited rigorous research on other tobacco smoking cessation interventions in these countries (Akanbi, et al, 2019). This is particularly vital in Jordan, where only 20% of smokers reported receiving medical advice to quit smoking, and approximately 63% had tried to quit but failed (Jaghbir, et al., 2014). In fact, nurses and physicians in Jordan do not usually recognize the addictive aspect of smoking and did not receive formal training on tobacco cessation counseling (Shishani, et al., 2008). Regulations should address waterpipe tobacco smoking in relation to all articles of the FCTC and should also be informed by best available research evidence in Jordan assessing interventions designed to decrease waterpipe smoking (Nakkash, et al., 2022).

Element 2

**Category**	**Selected Option/Element:** ***implement supportive measures to taxation in order to support clients who will stop smoking.***
**Benefits**	• A guideline update reported that increasing attempts to quit smoking, treatment use, and rates of successful quitting (Fiore, et al., 2008).<br>• A single study reported that medical insurance coverages that reimburse tobacco cessation interventions may increase the possibility that physicians will intervene with smokers and provide comprehensive counseling (McAfee, et al., 2015).<br>• The National Center for Chronic Disease Prevention and Health Promotion office reported that among established tobacco smokers, cessation leads to a substantial reduction

Category	Selected Option/Element: ***implement supportive measures to taxation in order to support clients who will stop smoking.***
	in risk, with the largest reductions among individuals who stop smoking before age 40 years (National Center for Chronic Disease Prevention and Health Promotion, 2014).<br>• A single study in Jordan reported that for a treatment cohort of 527,118 Jordanian male smokers who intended to quit, 103,970 life years were gained using the varenicline regimen, while 64,030 life years were gained using the NRT regimen as compared to the control group of life years. The cost per life year gained was $1696 USD for varenicline and $1890 USD for NRT (Mada'een et al., 2020).<br>• A meta-analysis showed that NRT, brief advice, and behavioral counseling can be effective in helping smoking cessation in low- and middle-income countries (Akanbi, et al, 2019).
**Potential harms**	• A single study found that about 46% of all cigarettes sold in Jordan in 2012 are illicit smuggled cigarettes (El- El-Khushman, et al., 2008).<br>• WHO reports found that people might switch to cheaper brands as it was documented that the most popular cigarette brands were the cheapest in Jordan (WHO, 2017; 2019).
**Cost**	Cost effectiveness was not reported by the evidence
**Uncertainty** regarding benefits and potential harms	• A systematic review and meta-analysis reported that there is still limited rigorous research on other tobacco smoking

Category	Selected Option/Element: ***implement supportive measures to taxation in order to support clients who will stop smoking.***
element were pursued)	cessation interventions in these countries (Akanbi, et al, 2019).<br>• A single regional study found that regulations should address waterpipe tobacco smoking in relation to all articles of the FCTC and should also be informed by best available research evidence in Jordan assessing interventions designed to decrease waterpipe smoking (Nakkash, et al., 2022).
**Stakeholders' views**	N/A
**Equity considerations**	• A meta analysis and systematic review demonstrated that NRT, brief advice, and behavioral counseling can be effective in helping smoking cessation in low- and middle-income countries (Akanbi, et al, 2019).

Implementation Considerations

Several implementation considerations are required at multiple levels (citizen, organization, and systems) to effectively develop and enforce a policy to increase tax on cigarette and waterpipe smoking products in Jordan. Barriers to implementing such policy can be overcome at several levels.

Below is the list of some of the most important implementation considerations:

- The WHO fact sheet in 2015 showed that the tobacco industry in Jordan has been opposing tobacco control efforts and inhibiting the execution and implementation of related legislations (World Health Organization, 2015) and making efforts through direct

and indirect lobbying such as participation with Jordan Standards and Metrology Organization to influence ministers and parliamentarians to push for more tobacco friendly policies (Jordan Restaurant Association, 2017).

- The tobacco industry utilizes top stakeholders and policymakers to exert pressure at all levels of the government to prevent FCTC full implementation and enforcement.
- The MOH letter in Jordan reported that an increase in tobacco use, especially waterpipe smoking, was reported recently due to the lobbying with the Jordan Restaurant Association (Ministry of Health, 2011a, 2011b, 2018).
- The Jordan Restaurant Association published a law including that waterpipe smoking is still provided inside cafes and restaurants impeding the implementation of the smoke-free law in public places as more than 700 places serving waterpipe are licensed in Jordan (Jordan Restaurant Association, 2017) with much higher number of places that do not have a license.
- According to the WHO fact sheet in 2013, the tobacco industry responded to tax raise on tobacco products by reducing the price of cigarettes by 25% (World Health Organization, 2015) claiming that lowering the price could reduce cigarette smuggling, but the truth was to keep cigarettes affordable (Ministry of Health; World Health Organization, 2015).
- The WHO report demonstrated that the tobacco companies in Jordan have been making efforts to improve their image with the media, community, and government alike (World Health Organization, 2013).
- A single study by Toukan (2016) reported that the Average poorest male cigarette smokers with a monthly income of 100-250 JD spend 25 times more on cigarettes than on health, 10 times more on cigarettes than on education, 1.5 times more on cigarettes than on food, and 2.5 times more on cigarettes than on housing.
- A single study found that Black market, illicit cigarette trade, fake cigarettes, E-cigarettes, or other types of cheap drugs could be a satisfactory, yet risky alternative. About 46% of all cigarettes sold in Jordan in 2012 are illicit smuggled cigarettes (El-Khashman, et al., 2008).
- The WHO reports reported that people might switch to cheaper brands as it was documented that the most popular cigarette brands were the cheapest in Jordan (WHO, 2017; 2019).

- A single study in Jordan reported that only 20% of smokers reported receiving medical advice to quit smoking, and approximately 63% had tried to quit but failed (Jaghbir, et al., 2014).
- A single study in Jordan reported that nurses and physicians in Jordan do not usually recognize the addictive aspect of smoking and did not receive formal training on tobacco cessation counseling (Shishani, et al., 2008).

Table 2 below demonstrates the potential barriers that could influence the successful implementation of these policies or programmatic options/elements as well as the counterstrategies to overcome these barriers.

Level	Barriers	Counterstrategies
**Individual/Family level**	1. Smokers' affordability to buy cigarettes after tax increase is affected by changes in household income (WHO FCTC, 2014).<br>2. Some behavioral changes that people who smoke could do as a result of higher tobacco taxes, hence muting the impact of raise:<br>➢ Converting into cheaper and less taxed brands<br>➢ Using more price-reducing promotions (coupons and special offers) offered by tobacco companies.<br>➢ Purchasing larger amounts of tobacco for multipack discounts<br>➢ Buying black-market cigarettes (Goldman, 2016).	1. When increasing taxes annually, there is a need to take into account the changes in household income to make sure affordability decreases and thus quit rates increase (WHO FCTC, 2014).<br><br>2. Tobacco cessation programs to decrease rates of smoking and motivate smokers to quit (National Center for Chronic Disease Prevention and Health Promotion (US) Office, 2014). Raising tobacco taxes can decrease tobacco smoking in the Eastern Mediterranean region (chalak, et al., 2021; Immurana, Boachie, & Iddrisu, 2021).<br><br>3. The subsequent increase in tobacco tax revenues would also be

	3. Basic family necessities and priorities (food, etc..) can be affected especially if smokers continue to buy high price tobacco products (Cancer Council NSW, 2016).	instrumental in covering expenditures related to such tobacco prevention and control programs (Ho, et al., 2018).<br><br>4. Strong enforcement against smuggling (Schröder D., Bóta G., & Sierra MM., 2021, West R., 2008).
**Organization (MOH, MOF, tax income, tobacco companies**	1. If price increase and hence affordability decrease, the government revenue of tobacco might be affected.<br><br>2. Increase in illicit tobacco trade as an alternative product for the taxed tobacco, which leaves the increase in taxes meaningless (WHO, 2013).<br>3. Since the ratification of the FCTC treaty in 2004 Jordan has shown evidence of some regulation of tobacco companies interactions, however, the index score shows that the level of industry interference is high (Al-Zawawi, 2019).<br>4. Tobacco companies might search for solutions to the customers such as a reduction in the original price of tobacco products in Jordan to compensate for lower purchase.<br>5. Other possible alternatives by the tobacco companies are to reduce size of cigarettes, or producing, low	1. Search for alternatives to compensate government loss in revenue from taxed tobacco products.<br>2. Banning sponsorship and social responsibilities activities of the tobacco industry (Al-Zawawi, 2019).<br>3. Provide transparency (Al-Zawawi, 2019).<br>4. Limit their interaction with governmental employees (Al-Zawawi, 2019).<br>5. Forces employees to disclose all meetings and necessary interactions with the industry (Al-Zawawi, 2019).<br>6. Prohibit all governmental representatives from accepting gifts/support from the industry (Al-Zawawi, 2019).<br>7. Prohibit tobacco industry from participation in policy level decisions (Al-Zawawi, 2019).<br>8. Prohibits government representatives from

	quality cigarettes both of which force the customer to buy even more cigarettes (National Center for Chronic Disease Prevention and Health Promotion (US) Office on Smoking and Health (2012).	endorsing/supporting tobacco industry initiatives (Al-Zawawi, 2019).
**System**	1. The main causes for the tobacco industry and their front groups influences are the lack of laws or regulations that limit such interferences (Al-Zawawi, 2019).<br>2. Stakeholders' Lobbyists of tobacco companies can exert pressure on the government to reduce tax after the increase to not enforce policies of tax increase on tobacco products (MOH &WHO, 2015; Jordan Standards and Metrology Organization, 2012b; Ministry of Health, 2014b; Global Tobacco Index, 2020).<br>3. Increase in illicit tobacco trade as an alternative product for the taxed tobacco, which leaves the increase in taxes meaningless (WHO, 2013).<br>4. Smuggling will increase.	1. Tobacco control policies need to be strictly enforced and monitored closely to ensure sustainability.<br>2. To reduce waterpipe smoking, no new licenses were issued to cafes and restaurants since 2017 in Jordan (Prime Ministry of Jordan, 2017).<br>3. Effective policies to counteract tobacco smuggling and other types of illicit tobacco should be enforced in parallel with tax increase on tobacco products in order to eliminate illicit trade in tobacco products (World Bank Group, 2019).<br>4. Excise rates for cigarettes, water-pipe tobacco, and other tobacco products should be annually increased by at least 20% to ensure tobacco affordability reduction (World Bank Group, 2019).<br>5. Tobacco use surveillance and monitoring should be further strengthened in Jordan, including a regular collection and public

		presentation of information on sales of cigarettes and other tobacco products, their prices as well as other economic indicators (World Bank Group, 2019).<br>6. Effective policies to counteract tobacco smuggling and other kinds of illicit tobacco sales should be implemented in line with the provisions of the FCTC Protocol to Eliminate Illicit Trade in Tobacco Products, which is recommended to be ratified by the country (World Bank Group, 2019).<br>7. Higher elasticity is likely to be obtained by introducing/increasing specific excise taxes and concerted efforts to implement non-price (tax) related WHO FCTC measures, such as cessation programs, bans on advertisement, and improving public awareness (Ho, et a., 2018).<br>8. Actions to prevent smuggling need to be developed.

Next Steps

This Policy Brief aims at nurture dialogue informed by the best available national, regional, and international evidence. The intention is not to advocate specific policy elements or close off discussion. Further actions will flow from the deliberations that the policy brief is intended to inform. These may include:

- Discussion and debate, if necessary, among stakeholders and policymakers regarding the policy elements described in this Policy Brief.

- Refining elements through integrating, revising or removing some components in this Policy Brief.
- Gaining political support is critical to initiate the required changes, which will necessitate presenting evidence to support implementation of Article 5.3 guidelines, advocacy to obtain grassroots support

References

- Action on Smoking and Health. (2014). What's in a cigarette? London: ASH. Retrieved from: http://www.ash.org.uk/files/documents/ASH_117.pdf
- Action on Smoking and Health. (2016). Smoking statistics: Illness and death. London: ASH. Retrieved from: http://www.ash.org.uk/files/documents/ASH_107.pdf
- Ahmad, S., & Franz, G. A. (2008). Raising taxes to reduce smoking prevalence in the US: a simulation of the anticipated health and economic impacts. *Public health*, 122(1), 3–10. https://doi.org/10.1016/j.puhe.2007.02.020
- Akanbi, M. O., Carroll, A. J., Achenbach, C., O'Dwyer, L. C., Jordan, N., Hitsman, B., ... & Murphy, R. (2019). The efficacy of smoking cessation interventions in low-and middle-income countries: A systematic review and meta-analysis. *Addiction*, 114(4), 620-635.
- Akl, E. A., Ward, K. D., Bteddini, D., Khaliel, R., Alexander, A. C., Lotfi, T., Alaouie, H., & Afifi, R. A. (2015). The allure of the waterpipe: a narrative review of factors affecting the epidemic rise in waterpipe smoking among young persons globally. *Tobacco control*, 24 Suppl 1(Suppl 1), i13–i21. https://doi.org/10.1136/tobaccocontrol-2014-051906
- Alefan, Q., Al-Issa, E. T., Alzoubi, K. H., & Hammouri, H. M. (2019). Association of smoking with direct medical expenditures of chronic diseases in north of Jordan: a retrospective cohort study. *BMJ open*, 9(10), e031143. https://doi.org/10.1136/bmjopen-2019-031143
- Ali, M., & Jawad, M. (2017). Health Effects of Waterpipe Tobacco Use: Getting the Public Health Message Just Right. *Tobacco use insights*, 10, 1179173X17696055. https://doi.org/10.1177/1179173X17696055
- Al-Tammemi, A. B. (2021). Tobacco epidemic in Jordan: the time to act is now. Global Health Promotion. Online First. https://doi.org/10.1177/17579759211026181

- Arrazola, R. A., Ahluwalia, I. B., Pun, E., Garcia de Quevedo, I., Babb, S., & Armour, B. S. (2017). Current Tobacco Smoking and Desire to Quit Smoking Among Students Aged 13-15 Years - Global Youth Tobacco Survey, 61 Countries, 2012-2015. MMWR Morb Mortal Wkly Rep, 66(20), 533-537. doi:10.15585/mmwr.mm6620a3

- Bader P, Boisclair D, Ferrence R. Effects of Tobacco Taxation and Pricing on Smoking Behavior in High Risk Populations: A Knowledge Synthesis. International Journal of Environmental Research and Public Health. 2011;8(11):4118.

- Belbeisi A, Al Nsour M, Batieha A, Brown DW, Walke HT. A surveillance summary of smoking and review of tobacco control in Jordan. *Global Health.* 2009; 5: 18.

- Burki, T. K. (2019). Tobacco control in Jordan. *The Lancet Respiratory Medicine, 7*(5), 386.

- Cancer Council NSW (2016). The financial impacts of smoking on families and children: information for client [press release]. Retrieved from: https://www.cancercouncil.com.au/wp-content/uploads/2016/04/CAN10472_TT_Factsheet_Finance_1.pdf

- Chalak A, Nakkash R, Abu-Rmeileh NME, et al. (2021) Own-price and cross-price elasticities of demand for cigarettes and waterpipe tobacco in three Eastern Mediterranean countries: a volumetric choice experiment. *Tobacco Control* Published Online First: 30 June 2021. doi: 10.1136/tobaccocontrol-2021-056616

- Department of Statistics (2016). Industry Survey 2015. Retrieved from: Amman, Jordan http://dosweb.dos.gov.jo/wp-content/uploads/2017/08/Industry2015.pdf

- Drope J, S. N., Cahn Z, Drope J, Hamill S, Islami F, Liber A, Nargis N, Stoklosa M. (2018). The Tobacco Atlas. Retrieved from Atlanta: https://files.tobaccoatlas.org/wp-content/uploads/2018/03/TobaccoAtlas_6thEdition_LoRes.pdf

- El-Khushman, H. M., Sharara, A. M., Al-Laham, Y. M., & Hijazi, M. A. (2008). Cigarette smoking among health care workers at King Hussein Medical Center. *Journal of Hospital Medicine: An Official Publication of the Society of Hospital Medicine,* 3(3), 281-284.

- Fiore, M. C., Jaén, C. R., Baker, T. B., Bailey, W. C., Benowitz, N. L., Curry, S. J., ... & Wewers, M. E. (2008). Treating tobacco use and dependence: 2008 update. Rockville, MD: US Department of Health and Human Services.

- Fishman, P. A., Ebel, B. E., Garrison, M. M., Christakis, D. A., Wiehe, S. E., & Rivara, F. P. (2005). Cigarette tax increase and media campaign: cost of reducing smoking-related deaths. *American journal of preventive medicine*, *29*(1), 19-26.

- Global Tobacco Index (2020). How deadly is tobacco industry influence in Jordan? [Internet]. Pathumthani, Thailand: Global Tobacco Index, 2020. Retrieved from: https://reports.globaltobacco index.org/

- Goldman T. (2016) Tobacco Taxes. Tobacco Taxes. To curb smoking and raise revenue, governments impose taxes on tobacco products. Health Affairs Health Policy Brief 2016

- Hamadeh, R. R., Lee, J., Abu-Rmeileh, N., Darawad, M., Mostafa, A., Kheirallah, K. A., Yusufali, A., Thomas, J., Salama, M., Nakkash, R., & Salloum, R. G. (2020). Gender differences in waterpipe tobacco smoking among university students in four Eastern Mediterranean countries. *Tobacco induced diseases*, 18, 100. https://doi.org/10.18332/tid/129266

- Heydari, G., EbnAhmady, A., Lando, H. A., Chamyani, F., Masjedi, M., Shadmehr, M. B., & Fadaizadeh, L. (2017). Third study on WHO MPOWER Tobacco control scores in Eastern Mediterranean Countries 2011-2015. *Eastern Mediterranean Health Journal*, *23*(9), 598-603.

- Higashi H, Truong KD, Barendregt JJ, et al. Cost Effectiveness of Tobacco Control Policies in Vietnam: The Case of Population-Level Interventions. *Applied health economics and health policy*. 2011;9(3):183-196.

- Ho, L. M., Schafferer, C., Lee, J. M., Yeh, C. Y., & Hsieh, C. J. (2018). Raising cigarette excise tax to reduce consumption in low-and middle-income countries of the Asia-Pacific region: a simulation of the anticipated health and taxation revenues impacts. *BMC public health*, 18(1), 1-9. https://www.dropbox.com/s/rhtibti0hktjppj/IMG-20181125-WA0021.jpg?dl=0

- Immurana, M., Boachie, M. K., & Iddrisu, A. A. (2021). The effects of tobacco taxation and pricing on the prevalence of smoking in Africa. *Global health research and policy*, 6(1), 14. https://doi.org/10.1186/s41256-021-00197-0

- Jaghbir, M., Shareif, S., & Ahram, M. (2014). Quitting smoking and utilization of smoking cessation services in Jordan: a population-based survey. *EMHJ-Eastern Mediterranean Health Journal*, 20(9), 538-546.

- Jawad, M., Abdulrahim, S., & Daouk, A. (2016). The Social Patterning of Tobacco Use Among Women in Jordan: The Protective Effect of Education on Cigarette Smoking and the Deleterious Effect of Wealth on Cigarette and Waterpipe Smoking. *Nicotine & Tobacco Research*, 18(4), 379-385. doi:10.1093/ntr/ntv111

- Jawad, M., Lee, J. T., & Millett, C. (2016). Waterpipe Tobacco Smoking Prevalence and Correlates in 25 Eastern Mediterranean and Eastern European Countries: Cross-Sectional Analysis of the Global Youth Tobacco Survey. *Nicotine & Tobacco Research*, 18(4), 395-402. doi:10.1093/ntr/ntv101

- Jawad, M., & Roderick, P. (2017). Integrating the impact of cigarette and waterpipe tobacco use among adolescents in the Eastern Mediterranean Region: a cross-sectional, population-level model of toxicant exposure. *Tobacco Control*, 26(3), 323-329. doi:10.1136/tobaccocontrol-2015-052777

- Jha, P., & Peto, R. (2014). Global effects of smoking, of quitting, and of taxing tobacco. *New England Journal of Medicine*, *370*(1), 60-68.

- Jordan Restaurant Association (2017). Touristic Restaurants and Public Health Law 47/2008. Retrieved from: https://www.dropbox.com/s/df62qp0tsk6y7mb/C_JRA%20work%20on%20tobacco%20lobbying%20%20English_Research%20%2B%20Toolkits%20%2B%20Guidelines%20%2B%20Technical%20Reports_2017_JRA_JRA.pptx?dl=0

- Jordan Standards and Metrology Organization (2000) Law 22/2000, 22/2000 C.F.R. (2000).

- Jordanian Standard and Metrology Organization (2012a). 466/2012 Technical Regulation 466/2012 C.F.R. Amman, Jordan (2012).

- Jordan Standards and Metrology Organization (2012b). Meeting Minutes of Cigarettes Technical Committee. Amman, Jordan. Retrieved from: https://www.dropbox.com/s/6o720gxypm1s3oe/%D8%A7%D9%84%D9%85%D9%88%D8%A7%D8%B5%D9%81%D8%A7%D8%AA%20%D9%88%D8%A7%D9%84%D9%85%D9%82%D8%A7%D9%8A%D9%8A%D8%B30001.pdf?dl=0

- Jordan Standard and Metrology Organization (2014a).787/2014 Technical Regulation 787/2014 C.F.R. 2014. Amman, Jordan

- Jordan Standards and Metrology Organization (2014b). Tobacco and tobacco products - Tobacco molasses JS 787:2014. In (Vol.787:2014): Jordan Standard and Metrology Organization. 2014. Amman, Jordan

- Madae'en, S., Obeidat, N., & Adeinat, M. (2020). Using cost-effectiveness analysis to support policy change: varenicline and nicotine replacement therapy for smoking cessation in Jordan. *Journal of pharmaceutical policy and practice*, 13(1), 1-8.

- Maraq.com. (2014). MPs demand to allow Argileh in cafes. Maqar News. Retrieved from: نواب يطالبون الإسراع بتشغيل مستشفى المفرق العسكري - صحيفة المقر(maqar.com)

- Mawya Al-Zawawi (2019). Framework Convention Alliance, Tobacco Industry Interference Index Jordan, Jordan.

- McAfee, T., Babb, S., McNabb, S., & Fiore, M. C. (2015). Helping smokers quit--opportunities created by the Affordable Care Act. *The New England journal of medicine*, 372(1), 5–7. https://doi.org/10.1056/NEJMp1411437

- Ministry of Interior (2006). Juvenile Conduct Law, (2006). Amman, Jordan

- Ministry of Health. (2007). STEPwise Approach to Chronic Disease Risk Factor Surveillance Survey 2007. Retrieved from: https://www.who.int/chp/steps/JordanSTEPS2007Report.pdf

- Ministry of Interior (2008) Traffic Law, (2008). Amman, Jordan.

- Ministry of Health (2008). Public Health Law 47/2008 and its Amendments, (2008).

- Ministry of Health (2011a). Meeting Minutes. Amman, Jordan Retrieved from: https://www.dropbox.com/s/r5b5irwaz2nux8u/P_Meeting%20Minutes%20of%20MOH%20MOT%20committee%20on%20touristic%20establishments.pdf?dl=0

- Ministry of Health. (2011b). Ministry of Health Letter Number TO/Tobacco/24 date 19/1/2011. Amman, Jordan Retrieved from: https://www.dropbox.com/s/3nuc0dkjodkrfs2/P_MOH%20letter%20to%20MOT%20on%20establishing%20a%20committee%20to%20review%20touristic%20establishments%20ban.pdf?dl=0

- Ministry of Health. (2014a). Global Youth Tobacco Survey Report 2014. Retrieved from: http://www.emro.who.int/images/stories/tfi/documents/GYTS_CR_JOR_2014.pdf

- Ministry of Health. (2014b). Report on Molasses Committee Amman, Jordan Retrieved from: https://www.dropbox.com/s/wt8u6cedcrtshiu/%D8%A7%D9%84%D9%85%D9%88%D8%A7%D8%B5%D9%81%D8%A7%D8%AA%20%D9%88%D8%A7%D9%84%D9%85%D9%82%D8%A7%D9%8A%D9%8A%D8%B310001.pdf?dl=0.

- Ministry of Health (2018). Ministry of Health Letter TO/Tobacco/672136/117. Amman, Jordan Retrieved from: https://www.dropbox.com/s/qqkp4dvjgdtrt16/IMG-20181125-WA0020.jpg?dl=0

- Nakkash R, Khader Y, Chalak A, et alPrevalence of cigarette and waterpipe tobacco smoking among adults in three Eastern Mediterranean countries: a cross-sectional household survey. *BMJ Open* 2022;12:e055201. doi: 10.1136/bmjopen-2021-055201

- National Center for Chronic Disease Prevention and Health Promotion (US) Office on Smoking and Health (2012). Preventing Tobacco Use Among Youth and Young Adults: A Report of the Surgeon General. Atlanta (GA): Centers for Disease Control and Prevention (US); 2012. Retrieved from: https://www.ncbi.nlm.nih.gov/books/NBK99237/

- National Center for Chronic Disease Prevention and Health Promotion (US) Office (2014). On Smoking and Health: The Health Consequences of Smoking—50 years of progress: a report of the surgeon general. US Centers for Disease Control and Prevention, Atlanta, GA2014. Retrieved from: https://aahb.org/Resources/Pictures/Meetings/2014-Charleston/PPT%20Presentations/Sunday%20Welcome/Abrams.AAHB.3.13.v1.o.pdf

- Prime Ministry of Jordan (2017). Prime Ministry letter number 64/12/1/28831 date 18/1/2017. Amman, Jordan Retrieved from: https://www.dropbox.com/s/szk8h2iixf2x1i1/P_PM%20letter%20to%20GAM%20and%20MOT%20approving%20their%20request%20on%2024-5-2017.pdf?dl=0

- Reitsma, M. B., Fullman, N., Ng, M., Salama, J. S., Abajobir, A., Abate, K. H., ... & Patton, G. C. (2017). Smoking prevalence and attributable disease burden in 195 countries and territories, 1990–2015: a systematic analysis from the Global Burden of Disease Study 2015. *The Lancet*, *389*(10082), 1885-1906.

- Rezaei, S., Sari, A. A., Arab, M., Majdzadeh, R., & Poorasl, A. M. (2016). Economic burden of smoking: a systematic review of direct and indirect costs. *Medical journal of the Islamic Republic of Iran*, 30, 397.

- Safi M, Al-tahat J. Jordan smoking rates highest in world amid claims of big tobacco interference. The Guardian 23 June 2020. Retrieved from: https:// www.theguardian.com/world/2020/jun/23/jordansmoking-rates-highest-in-world-amid-claims-of-bigtobacco-interference

- Salloum, R. G., Lee, J., Mostafa, A., Abu-Rmeileh, N. M., Hamadeh, R. R., Darawad, M. W., ... & Nakkash, R. (2019). Waterpipe tobacco smoking among university students in three Eastern Mediterranean countries: Patterns, place, and price. *Substance use & misuse*, *54*(14), 2275-2283.

- Schröder, D., Bóta, G., & Sierra, M. M. (2021). Combatting illicit tobacco trade: What is the role of EU law enforcement training? *Tobacco Prevention & Cessation*, 7.

- Shafey, O., S. Dolwick, and G. Guindon (2005). Tobacco control country profiles 2003. Atlanta (Georgia): American Cancer Society. 2005.

- Shepard DS, Halasa-Rappel YA, Al-Halaseh I, Fardous T, Jrasat M, Abu-Shaer M.(2017). Health Care Cost Study at Ministry of Health and the Cost and Financial Impact of Expanding the Civil Insurance Program to Vulnerable Jordanians and Syrian Refugees. UNICEF, Jordan. October 2017. Retrieved from: https://www.unicef.org/jordan/media/2671/file/Cost%20and%20financial%20impact%20Study.pdf

- Shishani, K., Nawafleh, H., & Froelicher, E. S. (2008). Jordanian nurses' and physicians' learning needs for promoting smoking cessation. *Progress in Cardiovascular Nursing*, 23(2), 79-83.

- Sweis, N. J., & Chaloupka, F. J. (2014). The economics of tobacco use in Jordan. *Nicotine & Tobacco Research*, 16(Suppl_1), S30-S36.

- Toukan, A. M. (2016). The economic impact of cigarette smoking on the poor in Jordan. *Value in health regional issues*, 10, 61-66.

- United Nations Treaty Collection. (2019). Framework Convention on Tobacco Control Retrieved from: https://treaties.un.org/pages/ViewDetails.aspx?src=TREATY&mtdsg_no=IX&chapter=9&clang=_en

- Waziry, R., Jawad, M., Ballout, R. A., Al Akel, M., & Akl, E. A. (2017). The effects of waterpipe tobacco smoking on health outcomes: an updated systematic review and meta-

analysis. *International journal of epidemiology*, 46(1), 32–43.
https://doi.org/10.1093/ije/dyw021

- West, R., Townsend, J., Joossens, L., Arnott, D., & Lewis, S. (2008). Why combating tobacco smuggling is a priority. *BMJ*, 337.

- World Health Organization (2003). WHO Framework Convention on Tobacco Control. Geneva: World Health Organization.

- World Health Organization (2010). WHO technical manual on tobacco tax administration. 2010: World Health Organization. Geneva. Retrieved from: https://apps.who.int/iris/handle/10665/44316.

- World Health Organization (2013). WHO report on the global tobacco epidemic, 2013: enforcing bans on tobacco advertising, promotion and sponsorship.

- World Health Organization, M.o.H. (2015). Jordan Tobacco Control Factsheet 2015. Retrieved from Amman, Jordan: https://www.dropbox.com/s/peklnawuok7qfy5/JOR_Factsheet_Tobacco%20Control%2C%202015_Eng%20copy.pdf?dl=0

- World Health Organization (2017a) WHO report on the global tobacco epidemic 2017. Monitoring tobacco use and prevention policies. 2017. Retrieved from: http://www.who.int/tobacco/global_report/en/.

- World Health Organization (2017b) WHO report on the global tobacco epidemic, 2017. Country profile: Jordan. 2017. Retrieved from: http://www.who.int/tobacco/surveillance/policy/country_profile/jor.pdf.

- World Health Organization (2019). Prevalence of Tobacco Smoking. Retrieved from: http://gamapserver.who.int/gho/interactive_charts/tobacco/use/tablet/atlas.html

- World Bank Group (2019). Global Tobacco Control program Brief. Jordan: Overview of Tobacco Use, Tobacco Control Legislation, and Taxation. Retrieved from: https://policycommons.net/artifacts/1284975/jordan/1881560/

- World Health Organization (2021). Jordan committed to implementing the WHO Framework Convention on Tobacco Control [Internet]. Geneva: World Health Organization, 2021. Retrieved from: http://www.emro.who.int/jor/jordan-news/implementing-fctc-in-jordan.html

- World Population Review (2021). Global country ranking by smoking rates [Internet]. World Population Review, 2021. Retrieved from: https://worldpopulationreview.com/country-rankings/ smoking-rates-by-country

Annex 1: Tobacco Expenditure and Consumption in Jordan: Household Surveys Data

	2002-2003	2006	2008	2010	2013
**Average annual household member expenditure on tobacco and cigarettes (in JD)**	34,8	47,1	60,3	78,9	94,8
**Average annual tobacco consumption per household member (in cigarette packs)**	52		55	62	66
**Average annual current income of household member (in JD)**	99,5	1083,7	1350,5	1660,2	1857,2
**No of household members**	5003251	5418932	5836892	6027943	6247808
**Total tobacco consumption (million cigarettes)**	5203		6421	7475	8247
**Total tobacco expenditure (in million JD)**	174		352	481	602
**Average calculated price of a 20-cigarettes pack**	0.7		1.1	1.3	1.4

Annex 2: Tobacco Affordability in Jordan

	2009	2010	2011	2012	2013	2014	2015	2016	2017	2018
**CPI$, tobacco and cigarettes**	100,7	112,1	102,9	101,4	90,4	115,0	103,5	103,2	107,9	114,7
**CPI, all items**	99,3	104,8	104,2	104,5	104,8	102,9	99,1	99,2	103,3	104,5
**GDP annual growth**	100,4	97,2	97,3	97,3	97,7	98,5	101,5	98.8	99,4	101,2
**Tobacco Affordability Index***	-1,0	-9.1	-1,5	0.2	13,3	-11,8	-2.8	-5,0	-4.8	-7,8

**% of GDP per capita required to purchase 2000 cigarette of the most sold brand*

$ Consumer Price Index

Table 1: The tax burden for cigarettes in Jordan for a pack of 20 cigarettes, in Fils (1JD=1000fils)

	**2013**	**2014**	**2018**
**Net-of tax price**	500	500	500
**Specific excise**	320	420	570
**Ad valorem excise (102%)**	510		
**Tiered specific excise**		653	809
**VAT, 16%**	133	172	221
**Total tax**	963	1245	1600
**Final retail price**	1463	1745	2100

Document 3

How to reduce the economic burden of tobacco in Palestine

Policy Brief

Prepared by

Sameera Awawda and Niveen Abu-Rmeileh

Institute of Community and Public Health (ICPH), Birzeit University

20/06/2022

Authors: Sameera Awawda and Niveen Abu-Rmeileh

Acknowledgment: Authors would like to acknowledge the work of Aisha Shalsh, Tamara Awad, Mariam Motan and Mais Aaraj who supported in implementing the search strategy and litmus tests with relevant stakeholders. Authors would also like to thank Dr. Rani abu Seir who kindly reviewed an earlier version of the policy brief.

Contents

Contents 41
Executive summary 42
Key messages 43
K2P policy brief: Full report 46
Problem Statement 46
Size of the problem 46
Underlying causes of the problem 48
Elements to address the problem 51
Element 1: Taxation 51
Element 2: Warning labels 55
Element 3: Prohibition of smoking in public places 57
Element 4: Increasing awareness among students 58
Implementation Considerations 60
Next steps 63
References 64

Executive summary

The current policy brief discusses the problem of the economic burden of tobacco smoking in Palestine. The policy brief provides empirical evidence on the size of the problem, underlying causes of the problem, elements to address the problem as well as barriers of implementation and counter strategies to overcome these barriers. There is a serious gap in the tobacco literature in Palestine in general. Moreover, there is no literature that attempted to estimate the economic burden associated with tobacco smoking in Palestine. The bulk of the empirical evidence used in this policy brief is based on other countries experiences in tobacco smoking and control policies used to reduce the prevalence of tobacco smoking. We argue that in order to reduce the overall economic burden of tobacco smoking, policymakers shall implement policies that seek to reduce the prevalence of tobacco smoking. Such tobacco control policies include tax increase; improving consumer education towards the harmful impact of smoking; the use of warning labels; restrictions on tobacco smoking in public spaces; bans on advertising, and promotion and smoking cessation programs. A cost-benefit analysis of the most feasible elements in the Palestinian context is provided.

Key messages

What is the problem?

The prevalence of tobacco smoking in Palestine has increased in the last decade by about 2%. Moreover, the prevalence of waterpipe smoking amongst women has more than doubled. Exposure to tobacco smoking is highly associated with high morbidities and high economic burden. Spending on tobacco in addition to the associated health costs may crowd out other expenditures on basic needs.

What do we know about the main elements of an approach to addressing the problem?

Element 1: Increasing price through taxation of tobacco (cigarettes and waterpipe) products.

- Increasing tobacco prices through taxation would reduce demand for tobacco products. Hence, income available for other goods and services or allocated to savings will be higher.
- A tax increase would have a long-terms health impact: prevent deaths and reduce associated healthcare costs.
- Effective taxation would generate additional revenues to the government. These revenues can be used to support other health programs such as UHC.
- For those who would continue on smoking at the same rate before the tax increase (because tobacco for them is a basic need), the economic burden of tobacco would further increase.
- Tax increase would reduce domestic sales of tobacco industry. Hence a decrease in the labor force working in the tobacco industry.
- Tax increase may further incentivize smuggling (illegal purchase of tobacco products).
- Enhance the substitution between tobacco products.
- The positive impact of taxation including income revenues at the household level and lives saved as well as the additional government revenues would exceed the negative impact including the reduction in the sales of local producers.
- Immediate taxation is better for individuals while other policies which start by intervention programs and ends with tax change are better for the government.
- Also, at least one study shows that in the presence of uncontrolled smuggling, tobacco tax policies might not be effective.

- Price/tax increases had the most positive equity impact (i.e., they yield a larger reduction in smoking prevalence and consumption among the lower SES groups). The poor households would benefit more than the richer from reducing tobacco consumption. Their welfare will be higher as they would have a higher disposable income. This is because cigarette consumption is concentrated amongst the poor who are more sensitive to price changes as compared to richer households.
- Increasing tobacco prices have a great effect on lowering the youth use of cigarettes.

Element 2: Using warning labels on cigarettes packs and waterpipe products.

- Using large graphical warnings is more effective in smoking cessation as compared to text warnings.
- Cigarette-stick warning which describe the economic cost of smoking is more effective in reducing the prevalence of smoking as compared to standard packaging warnings.
- At the individual level, using warning labels make people feel anxious, disgusted and avoid consuming some cigarettes packs with particular warning.
- Health warning on cigarettes packs have a long term impact in reducing the prevalence of tobacco smoking.
- The use of graphical warning on cigarettes packs is a low-cost way to reduce the prevalence of tobacco smoking.
- Using warnings on tobacco products might not be effective in reducing smoking prevalence when warnings do not comply with WHO FCTC regulations.

Element 3: Prohibition of smoking in public places.

- Complete smoking ban would significantly reduce the number of tobacco-attributable disease.
- Banning smoking in public spaces would improve the quality of life due to clean indoor air interventions.

- Minors’ exposure to second-hand smoking in public is very high. In the absence of effective laws and legislation, this may increase the prevalence of smoking among the youth.

Element 4: Increasing awareness among students.

- Public and mass-media campaigns about the health risks associated with tobacco smoking, especially among young people, would help reduce the prevalence of smoking or cessation attempts.
- Media campaigns shall use specific (graphical) messages that might be effective in increasing the incentives of quitting smoking but such campaigns might be expensive.

Implementation consideration

In order to effectively implement tobacco control policies that seek to reduce the economic burden of tobacco smoking via reducing smoking prevalence, a variety of barriers and counter strategies shall be highlighted.

K2P policy brief: Full report

Problem Statement

The prevalence of tobacco smoking in Palestine has increased in the last decade from 20.2% in 2011 to about 22% in 2019 (WHO, 2019). This rate is slightly higher than the global average where 20 of every 100 adults smoke tobacco. Moreover, the prevalence of tobacco smoking, particularly waterpipe smoking, amongst women has increased from 2.6% in 2011 (WHO, 2011) to 7% in 2019 (WHO, 2019). Indeed, tobacco smoking is shown to be one of the leading causes of morbidity (such as lung cancer, heart diseases, etc.) and mortality worldwide (West, 2017). In Palestine, lung cancer occupied the first rank of all cancer cases among men (17.3%) and the fourth among women (5.1%) (PCBS & MOH, 2012). Turning to the economic impact, with cigarette prices in Palestine being considered as one of the highest as compared to other countries in the region, spending on tobacco and the associated healthcare expenditures could crowd out other expenditures including basic needs. The persistent high prevalence and expenditure of tobacco smoking can be attributed to different factors including, the absence of effective tobacco control policies, low sensitivity to price changes, and smuggling.

Size of the problem

The prevalence of tobacco smoking ranges from about 4% to 24% in low-income countries and from about 3% to 45% in high-income countries (Nazir et al., 2019). In Palestine, a lower-middle income country, nearly 22 of every 100 adults currently smoke tobacco products. It is worth noting that, in Palestine, the prevalence of cigarette and waterpipe smoking is higher among men (Nakkash et al. 2022). Moreover, females' smokers are more likely to smoke waterpipe products as compared to cigarettes. Empirical evidence shows that the prevalence of cigarette and waterpipe smoking among men is 53.4% and 18.0% while the prevalence of cigarette and waterpipe smoking among women is 3.1% and 7.9% (Nakkash et al. 2022). According to age groups, the prevalence of smoking is relatively high among adolescents aged 10-19 years old in the West Bank (13.7%) (Veeranki et al., 2016). Moreover, the bulk of smokers (about 74%) usually started smoking at young age <18 years (Abu Seir at al. 2020).

Exposure to tobacco smoking is highly associated with high morbidities, high economic burden as well as high environmental burden. Tobacco smoking increases the risk of chronic

diseases such as lung cancer, heart diseases, chronic bronchitis and other diseases (Reddy 2021; Parascandola 2019; Gao et al., 2017; Doll, 2004). In addition, tobacco smoking leads to about 6 million premature deaths around the World (West, 2017). In Palestine, the expected lifetime of heavy smokers is about 5 years less than that of ex-smokers and about 7 years less than the life expectancy of nonsmokers (Brønnum-Hansen et al., 2018). Recent estimates show that lung cancer is the 2nd most commonly diagnosed type of cancer among men (6.6%) and the 10th among women (1.3%) in Palestine in 2020 (MoH, 2021). Moreover, lung cancer was the major cause of cancer-related deaths in the same year (18.8%).

Treatment of tobacco-related illnesses is very expensive, thus indirectly increases the economic burden of tobacco. The overall burden of chronic diseases including tobacco-related illnesses is increasing worldwide. This problem will be more prominent in the context where households would incur the bulk of health expenditures particularly in the absence of an effective health insurance program. Furthermore, illnesses related to tobacco smoking could have negative effects on labor productivity through either increasing unemployment, or decreasing the number of working days, or decreasing employees' performance (Hernández et al. 2021; Rizzo 2001). Empirical evidence shows that the average economic burden for lost productivity due to smoking is $4430 per year for smokers as compared to $2623 for nonsmokers (Bunn et al. 2006).

Moreover, tobacco expenditure is expected to have a remarkable impact on smokers' budget. In Palestine, available nationally representative data shows that the share of tobacco expenditure of households' total expenditure exceeds the share of expenditure on some necessary goods and services such as health expenditures (about 9% for tobacco expenditure vs. 4% for health expenditure)[3]. As a result, spending on tobacco may crowd out other expenditures on basic needs (e.g., John et al., 2012). Available global empirical evidence shows that tobacco products and food are substitutes. For example, a one percent decrease in tobacco prices will increase tobacco consumption and reduce food consumption by about 0.14-0.55 percent (e.g., John et al., 2012; Wang et al., 2006; Busch et al., 2004). Empirical evidence also shows that the crowding out effect is more prominent among low-income groups of households (John et al., 2012; Busch et al., 2004). Lastly, with the increasing prevalence rate among younger generation, which comprises the

[3] These are authors calculations based on the Palestinian Expenditure and Consumption (PECS) surveys.

bulk of the population, the future economic and health burden of tobacco smoking is expected to be very high.

Underlying causes of the problem

Governance arrangement

At the governance level, the Palestinian government is not committed yet to the WHO framework convention on tobacco control (FCTC). It is worth noting that the main purpose of the WHO FCTC is to provide protection against the harmful consequences of tobacco smoking and exposure from smoking (WHO FCTC article 3). Commitment to the WHO FCTC is supposed to reduce prevalence of tobacco smoking, hence the associated health, economics, social and environmental devastating consequences. For instance, empirical evidence that assesses tobacco control plans in the Eastern Mediterranean countries show that the implementation of tobacco control policies can indeed lead to significant reduction in smoking prevalence (Heydari et al., 2014; Usmanova and Mokdad, 2013).

Although Palestine is not committed to the WHO FCTC, there are some laws and regulations concerning tobacco control that have been implemented by the Palestinian government. However, these laws are very limited and even restricted to banning only cigarette smoking in public places and using simple health warnings on tobacco packages (WHO, 2015b, 2018). Further, in the public health law (no. 20, 2004), there was one general article (no. 44) regarding smoking which states that "*The ministry should take the suitable measures to limit the harm created by the spread of smoking.*" (Palestinian Legislative Council 2005). Empirical evidence shows that Palestine is performing very well in terms of smoke-free policies as compared to other countries in the region (Heydari et al. 2014; Usmanova and Mokdad 2013). Yet the application and control of these policies in public spaces is very limited.

Moreover, it is illegal to sell cigarettes to children under the age of 18, sell cigarettes individually, and advertising and promoting tobacco on television or in the media (Abu shomar wt al., 2014). Unfortunately, these laws are not enforced (Maraqa et al., 2020). In addition, warning on the dangers of tobacco are restricted to some sentences describing the harmful effects of tobacco on health without using any photos (WHO, 2018).

In terms of collaboration between stakeholders, there is poor collaboration of the different stakeholders (policymakers, academics, media and community) to discourage tobacco use through

different campaigns that are supported by the government, the media which guides the community perceptions toward the implementation of policy, corporate social responsibility, and health education. In addition, there is a very limited government budget to support tobacco control programs where government budget allocated to such programs has not exceed $40 000 (WHO, 2018).

Turning to the issue of illegal purchase of tobacco products, there is a poor control of smuggling which increases the consumption of tobacco products particularly among young people and low-income individuals. Also smuggling may lead local tobacco retailers to sell tobacco to young people less than 18 years of age (Joossens and Raw, 1998).

Financial arrangement

At the financial level, the prices of cigarettes are already high in Palestine (WHO, 2011). The cigarettes tax structure is a specific excise rate of 35.9%, Ad valorem excise rate of 33.8%, and a VAT rate of 13.8% (WHO, 2019). Higher taxation in Palestine did not result in a decrease in smoking prevalence during the last decade as shown in the outset. Moreover, high tobacco prices coupled with inelastic demand, particularly for waterpipe products[4] would increase the economic burden of tobacco smoking at the household level.

Regarding the health insurance system in Palestine, coverage of health care services and health care costs is still limited although the share of insured households is relatively high (Abu-Zaineh et al., 2020). According to the latest data available from the national health accounts, the share of out-of-pocket expenditure is about 40% (PCBS and MOH, 2020). This indicates that health insurance coverage is rather ineffective and that households will foot the bill of the tobacco-related disease burden.

Other factors

Of course, there are other causes contributing to the high prevalence of tobacco smoking and the associated economic burden. These include, *inter alia,* psychological factors as well as parental and cultural influence. Psychological factors have shown to increase the prevalence and consumption of tobacco smoking (Ünübol and Sayar 2019). These factors include depression, absence of self-confidence, experiencing traumatic life events, anxiety, and difficulty in expressing emotions (Ünübol and Sayar 2019; Habibi et al. 2018; Fu et al. 2007; Thorberg et al. 2006). In

[4] An estimation of elasticities of different tobacco products for both men and women is calculated by the authors based on a sample that has been prepared within the economics of waterpipe projects.

Palestine, there is a gap in the smoking related literature in general. Particularly, there are no studies that attempt to assess the possible association between psychological factors and smoking. It is though expected that, for instance, depression resulted from the general unstable political situation to be a main driver of not only of smoking but also of the high rates of cigarettes consumption in Palestine. Turning to the issue of cultural and parental influence, empirical evidence shows that the interaction with other smokers (friends and family members) is highly associated with smoking uptake particularly among the young segment of the population (Lochbuehler et al. 2016; Leonardi-Bee et al. 2011). This indicates that exposure to second-hand smoking is one factor that increases the prevalence of tobacco smoking. Accordingly, if more than one member in the households are smokers, then the economic burden would be high. Also of interest that culture has influence on the choice of tobacco product (cigarettes and waterpipe) especially among women. Empirical evidence shows that women tend to choose waterpipe over cigarettes as a more acceptable and less risky form of smoking (Salloum et al. 2017; Afifi et al. 2013). This explains the high prevalence of waterpipe smoking amongst Palestinian women as mentioned above.

Elements to address the problem

To reduce the overall economic burden of tobacco smoking, policymakers shall implement policies that seek to reduce the prevalence of tobacco smoking. Such tobacco control policies include tax increase; improving consumer education; the use of warning labels; restrictions on tobacco smoking in public spaces; bans on advertising and promotion and smoking cessation programs (Glasser and Robert, 2021; Bamir et al., 2020; Leao et al., 2018). Below is a detailed analysis of the costs and the benefits associated with the main control policies that deemed to be feasible in the context of Palestine. Table 1 summarized the main elements to address the problem.

**Table 1: Elements to address the problem of the economic burden associated with tobacco smoking**	
**Option/Element 1**	Increasing price through taxation of tobacco (cigarettes and waterpipe) products.
**Option/Element 2**	Using warning labels on cigarettes packs and waterpipe products.
**Option/Element 3**	Prohibition of smoking in public places.
**Option/Element 4**	Increasing awareness among students.

Element 1: Taxation

Two studies show that increasing tobacco prices through taxation is the most effective policy tool to reduce the prevalence of tobacco smoking (Salti et al., 2016; Chaloupka et al., 2011). Although increase in taxation may help reduce the prevalence of tobacco smoking and hence the associated economic burden, such a policy may be associated with potential harms and costs at both the micro- and macro-economic levels. The cost-benefit analysis of tobacco taxation is summarized in Table 2.

In terms of associated benefits, empirical evidence shows the existence of different benefits at the micro level (mainly health and economic benefits) and the macro level (at the government level). For instance, three systematic reviews and two single studies show that increasing tobacco prices through taxation would reduce demand for tobacco products. Hence, income available for other goods and services or allocated to savings will be higher (e.g., Leão et al., 2018; Prasetyoputra & Irianti, 2014; CDHS, 2012; Ciapponi, 2011; Hu and Mao, 2002). In terms of the health impact, two systematic reviews show that a tax increase would have a long-terms health

impact as it prevents deaths and reduces healthcare costs associated to tobacco-related diseases (e.g., Ciapponi, 2011; Hoffman and Tan, 2015). Moreover, one single study shows that effective taxation would generate additional revenues to the government (e.g., Chaloupka et al., 2012). These revenues can be used to support other health programs such as UHC.

In terms of the potential harms, similarly a tax increase may have adverse consequences at both the micro- and macro-levels. At the micro level, one single study shows that for those who would continue on smoking at the same rate before the tax increase (because tobacco for them is a basic need or tobacco consumption is inelastic), the economic burden of tobacco would further increase (Santoso & Erlando, 2020). At the macro level, one systematic review and one single study show that a tax increase would reduce domestic sales of tobacco industry, hence results in a decrease in the labor force working in the tobacco industry (e.g., Mckay et al., 2015; Delipalla 2009). Furthermore, one systematic review shows that a tax increase may further incentivize illegal purchase of tobacco products – smuggling (e.g., Brown et al., 2014). Also of interest that an increase in the taxation on a specific tobacco product would enhance the substitution between tobacco products where smokers tend to switch to cheaper tobacco products (Jawad et al., 2016). The substitution between tobacco products is a function of the own-price elasticity of each product as well as cross-price elasticity among products. One single study shows that the demand for both premium and discount cigarettes is elastic in Palestine where the own price elasticities were -1.04 and -1.21, respectively (Chalak et al. 2021). The study also shows that premium and discount tend to be complements in Palestine with cross-price elasticity of -0.64. A substitution effect was, however, found between some cigarettes and waterpipe products. For example, the cross-price elasticity between premium cigarettes and premium waterpipe was 0.29. These results together may imply that smokers would reduce their consumption of cigarettes following a tax increase (own-price elasticity >1) and switch to waterpipe products (cross-price elasticity >0). Accordingly, in order to reduce the prevalence of tobacco smoking and consumption expenditure, both cigarettes (local and imported) and waterpipe products shall be taxed.

In general, three single studies show that the positive impact of taxation including income revenues at the household level and lives saved as well as the additional government revenues would exceed the negative impact including the reduction in the sales of local producers (e.g., Baker et al., 2018; Arslanhan et al., 2011; Van Baal et al., 2007). Moreover, one single study shows that immediate taxation is better for individuals while other policies which start by intervention

programs and ends with tax change are better for the government (e.g., Arslanhan et al., 2011). Nonetheless, most of related systematic reviews and studies show that the impact of taxation on the economic burden of tobacco is a function of elasticity. The degree of sensitivity to price changes vary across tobacco products and socioeconomic groups. So the net budget impact might be positive, negative or neutral depending on the percent change in tobacco consumption. Also, one single study shows that in the presence of uncontrolled smuggling, tobacco tax policies might not be effective (Brown et al., 2014).

Two systematic reviews and two single studies show that the increase in the prices of tobacco products had the most positive equity impact as it yields a significant reduction in smoking prevalence and consumption among the lower SES groups (e.g., Brown et al, 2014; Bader et al., 2011; Amos et al., 2011; Fayter et al., 2008). The poor households would benefit more than the richer from reducing tobacco consumption. Their welfare will be higher as they would have a higher disposable income. This is because cigarette consumption is concentrated amongst the poor who are more sensitive to price changes as compared to richer households. Moreover, one single study shows that increasing tobacco prices have a great effect on lowering the youth use of cigarettes (Kong et al., 2019).

A tax increase might not be supported by all relevant stakeholders including smokers, nonsmokers and policymakers. Three single studies show that smokers would not support a tax increase of cigarettes as prices are already high (e.g., Foley & Balázs, 2010; Arslanhan et al., 2011; Vardavas et al., 2012). In addition, domestic tobacco producers would not support a tax increase as one single study shows that taxation on tobacco would reduce their sales and profits (WHO, 2015c; Warner, 2000). Given its effectiveness in reducing cigarettes consumption, increasing excise tax on all waterpipe products is crucial to reduce its consumption and economic burden.

Table 2: Key findings from systematic reviews and single studies on element 1

**Category**	**Element 1: Tax increase**
**Benefits**	● Increasing tobacco prices through taxation would reduce demand for tobacco products. Hence, income available for other goods and services or allocated to savings will be higher (e.g., Leão et al., 2018; Prasetyoputra & Irianti, 2014; CDHS, 2012; Ciapponi, 2011; Hu and Mao, 2002).

	● A tax increase would have a long-terms health impact: prevent deaths and reduce associated healthcare costs (e.g., Ciapponi, 2011; Hoffman and Tan, 2015).<br>● Effective taxation would generate additional revenues to the government (e.g., Chaloupka et al., 2012). These revenues can be used to support other health programs such as UHC.
**Potential harms**	● For those who would continue on smoking at the same rate before the tax increase (because tobacco for them is a basic need), the economic burden of tobacco would further increase (Santoso & Erlando, 2020).<br>● Tax increase would reduce domestic sales of tobacco industry. Hence a decrease in the labor force working in the tobacco industry (e.g., Mckay et al., 2015; Delipalla 2009).<br>● Tax increase may further incentivize smuggling (illegal purchase of tobacco products) (e.g., Brown et al., 2014).<br>● Enhance the substitution between tobacco products (Jawad et al., 2016).
**Cost**	● The positive impact of taxation including income revenues at the household level and lives saved as well as the additional government revenues would exceed the negative impact including the reduction in the sales of local producers (e.g., Baker et al., 2018; Arslanhan et al., 2011; Van Baal et al., 2007).<br>● Immediate taxation is better for individuals while other policies which start by intervention programs and ends with tax change are better for the government (e.g., Arslanhan et al., 2011).
**Uncertainty**	● Most of related systematic reviews show that the impact of taxation on the economic burden of tobacco is a function of elasticity. The degree of sensitivity to price changes vary across tobacco products and socioeconomic groups. So the net budget impact might be positive,

	negative or neutral depending on the percent change in tobacco consumption.<br>● Also, at least one study shows that in the presence of uncontrolled smuggling, tobacco tax policies might not be effective (Brown et al., 2014).
**Stakeholders' views**	● Smokers would not support a tax increase of cigarettes as prices are already high in the country (e.g., Foley & Balázs, 2010; Arslanhan et al., 2011; Vardavas et al., 2012).<br>● Domestic tobacco producers would not support a tax increase as this would reduce their sales and profits.
**Equity considerations**	● Price/tax increases had the most positive equity impact (i.e., they yield a larger reduction in smoking prevalence and consumption among the lower SES groups). The poor households would benefit more than the richer from reducing tobacco consumption. Their welfare will be higher as they would have a higher disposable income. This is because cigarette consumption is concentrated amongst the poor who are more sensitive to price changes as compared to richer households (e.g., Brown et al, 2014; Bader et al., 2011; Amos et al., 2011; Fayter et al., 2008).<br>● Increasing tobacco prices have a great effect on lowering the youth use of cigarettes (Kong et al., 2019).

Element 2: Warning labels

Waterpipe products shall carry warning labels comparable to those used for cigarettes products and shall comply with the WHO FCTC regulations. Table 3 summarizes the cost-benefit analysis of using warning labels on tobacco products. One systematic review shows that using large graphical warnings is more effective in smoking cessation as compared to text warnings (Hammond, 2011). One systematic review shows that using graphical warning on cigarettes packs is also an effective and a low-cost way to reduce the prevalence of tobacco smoking (Francis et

al., 2017). Two systematic reviews show that using warning labels make people feel anxious, disgusted and avoid consuming some cigarettes packs with particular warning (e.g., Drovandi et al., 2019; Francis et al., 2017). One single study shows that health warning on cigarettes packs have a long term impact in reducing the prevalence of tobacco smoking (White et al., 2019). Moreover, one single study shows that using cigarette-stick warning which describe the economic cost of smoking is more effective in reducing the prevalence of smoking as compared to standard packaging warnings (Drovandi et al., 2019). On the other hand, one systematic review shows that using warnings on tobacco products might not be effective in reducing smoking prevalence when warnings do not comply with WHO FCTC regulations (Tee et al., 2015). In conclusion, using health warning labels mainly large graphical labels on waterpipe and cigarettes products can be a cost-effective option to reduce the prevalence of tobacco smoking and the associated economic burden. This is because warning labels is a cheap strategy as compared to other strategies and is effective in enhancing smoking cessation as mentioned above.

Table 3: Key findings from systematic reviews and single studies on element 2

**Category**	**Element 2: Warning labels**
**Benefits**	● Using large graphical warnings is more effective in smoking cessation as compared to text warnings (e.g., Hammond, 2011).<br>● Cigarette-stick warning which describe the economic cost of smoking is more effective in reducing the prevalence of smoking as compared to standard packaging warnings (e.g., Drovandi et al., 2019).<br>● At the individual level, using warning labels make people feel anxious, disgusted and avoid consuming some cigarettes packs with particular warning (e.g., Drovandi et al., 2019; Francis et al., 2017).<br>● Health warning on cigarettes packs have a long term impact in reducing the prevalence of tobacco smoking (White et al., 2019).
**Cost**	● The use of graphical warning on cigarettes packs is a low-cost way to reduce the prevalence of tobacco smoking (Francis et al., 2017).

**Uncertainty**	- Using warnings on tobacco products might not be effective in reducing smoking prevalence when warnings do not comply with WHO FCTC regulations (Tee et al., 2015).

Element 3: Prohibition of smoking in public places

Table 4 discusses the cost-benefit analysis of prohibiting smoking in public spaces. One single study shows that prohibiting tobacco smoking in public spaces may gain a great deal of support compared to other tobacco policy interventions (Mamudu et al., 2020). One study and one systematic review show that complete smoking ban would significantly reduce the number of tobacco-attributable diseases (Donaldson et al., 2011; Meyers et al., 2009). Two single studies report an improvement in quality of life due to clean indoor air interventions (Hahn, 2010; Goodman et al., 2007). One single study shows that banning smoking in restaurant and bars have no negative economic impact (Scollo, 2003). One single study shows that West Bank and Gaza Strip have one of the highest scores in term of smoke-free policies as compared to other countries in the region (Heydari et al., 2014). However, many public spaces are not smokeless in practice. This requires effective implementation of banning policies in public places as well as regular control on how public spaces are committed to such policies. Moreover, the prohibition of selling tobacco products in public spaced to minors should be enhanced. One single study shows that minors' exposure to second-hand smoking in public is very high (Christophi et al., 2008). In the absence of effective laws and legislation, this may increase the prevalence of smoking among the youth. Thus, governments shall enforce laws that prohibit the sale of tobacco to minors as well as smoking in public spaces.

Table 4: Key findings from systematic reviews and single studies on element 4

**Category**	**Element 3: Prohibition of smoking in public places**
**Benefits**	- Complete smoking ban would significantly reduce the number of tobacco-attributable disease (e.g., Donaldson et al., 2011; Meyers et al., 2009).

	• Banning smoking in public spaces would improve the quality of life due to clean indoor air interventions (Hahn, 2010; Goodman et al., 2007).
**Potential harms**	• Banning smoking in restaurant and bars have no negative economic impact (Scollo, 2003).<br>• Minors' exposure to second-hand smoking in public is very high (Christophi et al., 2008).
**Stakeholders' views**	• Prohibiting tobacco smoking in public spaces may gain a great deal of support compared to other tobacco policy interventions (Mamudu et al., 2020).

Element 4: Increasing awareness among students

Another important policy intervention is about increasing awareness among students (Table 5). The government, through the ministry of education, shall initiate anti-smoking awareness programs that targets students and provide them with the information about the health and non-health harmful effects of smoking. Two systematic reviews show that public and mass-media campaigns about the health risks associated with tobacco smoking, especially among young people, would help reduce the prevalence of smoking or cessation attempts (Mckay et al. 2015; Wilson et al. 2012). Two single studies show that media campaigns shall use specific (graphical) messages that might be effective in increasing the incentives of quitting smoking but such campaigns might be expensive (National Cancer Institute 2008; Wakefield et al. 2003).

In Palestine, one single study, which was conducted in the West Bank among 10th grade female students, shows that about 93% of adolescents were aware of the harmful effects of cigarettes (Damiri et al. 2020). The study also shows that the prevalence of waterpipe smoking among this segment of population is high. This entails that young people tend to smoke even though they are well aware of the harmful consequences of smoking. Accordingly, for this policy intervention to be efficient in changing the smoking behavior of school children, it shall be accompanied with restrictions on and control over the sale of tobacco products to minors as well as implementing wide campaigns that targets not only school children but their parents.

Table 5: Key findings from systematic reviews and single studies on element 4	
**Category**	**Element 4: Increasing awareness among students**
**Benefits**	• Public and mass-media campaigns about the health risks associated with tobacco smoking would help reduce the prevalence of smoking or cessation attempts (e.g., Mckay et al. 2015; Wilson et al. 2012).
**Cost**	• Media campaigns shall use specific (graphical) messages that might be effective in increasing the incentives of quitting smoking but such campaigns might be expensive (National Cancer Institute 2008; Wakefield et al. 2003).

Implementation Considerations

The potential barriers that could influence the successful implementation of tobacco policies introduced above are summarized in Table 6.

Table 6: Implementation considerations and counter strategies		
**Level**	**Barriers**	**Counterstrategies**
citizens	● One of the barriers at the individual level is social desirability: Smokers would not support a tax increase of cigarettes as prices are already high in the country (Vardavas et al., 2012; Arslanhan et al., 2011). Also, consumers became more accustomed to higher prices (e.g., Wright et al., 2017).<br>● The youth are vulnerable to social and environmental influences that promote tobacco use such as tobacco retails and cafes (e.g., Nakkash and Khalil, 2010; Vansickel, et al., 2012).<br>● Waterpipe smoking among youth is considered as a social activity and a less risky alternative to cigarettes (e.g., Salloum et al., 2017).<br>● The emergence of new forms of nicotine and tobacco products such as e-cigarettes and heated tobacco products which are popular, and easily accessible (McNeill et al., 2018; McKelvey et al., 2018).	● Increasing their awareness about the harmful effects of tobacco (lower risk of tobacco-related illness) and income gains from reducing tobacco consumption (Nishio et al., 2018). This could be done through for example warning mobile messages, social media. These messages may include health warnings related to smoking in general and to both cigarettes and waterpipe products (e.g., Heydari et al., 2013; Islam et al., 2016; Salloum et al., 2016).<br>● Cessation programs to help smokers quit (Jawad et al., 2016; Brown et al., 2014).

Organization Institutions/ Hospitals/universities	• Difficult to reach students who are not in the school/universities as well as pregnant women when considering health education programs that target these groups (e.g., Oncken et al., 2010; Karekla et al., 2009).<br>• The absence of the implementation of policies/ regulations that prevent smoking inside restaurants, workplace or other places (e.g., Salloum et al., 2017; Salloum et al., 2016).	• Mobile messages including health warnings for parents or youth or pregnant. Empirical evidence shows that those receiving related mobile phone messages had a significantly higher likelihood of quitting smoking (e.g., Vodopivec-Jamsek et al., 2012).<br>• Enforcing the clean indoor air laws (e.g., Salloum et al., 2017).<br>• Training health care workers in smoking cessation counseling (Nichter et al., 2018; Carson et al., 2012).

System Laws/policies/regulations	● Lack of control over smuggling (Prasetyoputra & Irianti, 2014).<br>● Lack of government resources to allocate for tobacco control policies (e.g., Mohamed et al., 2018).<br>● The absence of policies/ regulations that prevent smoking inside restaurants, workplace or other places (WHO, 2015).<br>● Tobacco industry will try to interfere with the policy-making process in hopes that prices will not be raised.<br>● Tobacco industry will use strategies to reduce prices in order for the taxation to not affect its consumers.	● The government can deter, detect and punish smuggling (e.g., Guindon et al., 2004).<br>● Revenues generated from taxation can be used to enhance other tobacco control programs such as cessation and health promotion (Chaloupka et al., 2012).<br>● To ratify, sign, and commit to the implementation of the WHO FCTC framework.<br>● Policies to increase tobacco prices through mechanisms other than taxation (Golden et al., 2016).

Next steps

The goals of this policy brief are two-fold. First is to engage stakeholders including policymakers and researchers in discussing the best elements to address the problem of the economic burden of tobacco smoking in Palestine. This will be achieved through a deep discussion between the different stockholders through a policy dialogue. Second, the current policy brief shall provide an indicator on the tobacco literature gab in Palestine, particularly the gab regarding the analysis of the economic burden of tobacco smoking.

References

Abu Seir, R., Kharroubi, A., & Ghannam, I. (2020). Prevalence of tobacco use among young adults in Palestine.

Abu Shomar, R. T., Lubbad, I. K., El Ansari, W., Khatib, I., & Alharazin, H. J. (2014). Smoking, awareness of smoking-associated health risks, and knowledge of national tobacco legislation in Gaza, Palestine.

Abu-Zaineh, M., Awawda, S., & Ventelou, B. (2020). Who bears the burden of universal health coverage? An assessment of alternative financing policies using an overlapping-generations general equilibrium model. Health policy and planning, 35(7), 867-877.

Afifi, R., Khalil, J., Fouad, F., Hammal, F., Jarallah, Y., Farhat, H. A., ... & Nakkash, R. (2013). Social norms and attitudes linked to waterpipe use in the Eastern Mediterranean Region. Social Science & Medicine, 98, 125-134.

Amos, A., Bauld, L., Clifford, D., Fidler, J., Hill, S., et al., 2011. Tobacco Control,Inequalities in Health and Action at the Local Level in England. PHRC, London.

Apollonio, D. E., & Glantz, S. A. (2020). Tobacco industry promotions and pricing after tax increases: An analysis of internal industry documents. Nicotine and Tobacco Research, 22(6), 967-974.

Arslanhan, S., Caner, A., Helvacioglu, K., Saglam, I., & Teksoz, T. (2012). An economic analysis of tobacco elimination policies in Turkey. Health Policy, 106(2), 149-160.

Asma, S., Warren, W., Althomsons, S., Wisotzky, M., Woollery, T., & Henson, R. (2004). Addressing the chronic disease burden with tobacco control programs. Public Health Reports, 119(3), 253-262.

Bader, P., Boisclair, D., & Ferrence, R. (2011). Effects of tobacco taxation and pricing on smoking behavior in high risk populations: a knowledge synthesis. International journal of environmental research and public health, 8(11), 4118-4139.

Baker, C. L., Ding, Y., Ferrufino, C. P., Kowal, S., Tan, J., & Subedi, P. (2018). A cost–benefit analysis of smoking cessation prescription coverage from a US payer perspective. ClinicoEconomics and outcomes research: CEOR, 10, 359.

Bamir, M., Masoud, A., Dehnavieh, R., & Ebrahimi, F. (2020). Suggesting Approaches of Tobacco Control for Policymakers: A Serious Challenge of Iranian Future Health. Addiction and Health, 12(4), 294-295.

Brønnum-Hansen, H., Jonassen, M., Shaheen, A., Duraidi, M., Qalalwa, K., & Jeune, B. (2018). The impact of smoking on expected lifetime with and without chronic disease among Palestinian men in the West Bank. The European Journal of Public Health, 28(3), 538-541.

Brown, T., Platt, S., & Amos, A. (2014). Equity impact of population-level interventions and policies to reduce smoking in adults: a systematic review. Drug and alcohol dependence, 138, 7-16.

Bunn III, W. B., Stave, G. M., Downs, K. E., Alvir, J. M. J., & Dirani, R. (2006). Effect of smoking status on productivity loss. Journal of Occupational and Environmental Medicine, 48(10), 1099-1108.

Busch, S., Jofre-Bonet, M., Falba, T. A., & Sindelar, J. L. (2004). Tobacco spending and its crowd-out of other goods.

Carson, K. V., Verbiest, M. E., Crone, M. R., Brinn, M. P., Esterman, A. J., Assendelft, W. J., & Smith, B. J. (2012). Training health professionals in smoking cessation. Cochrane database of systematic reviews, (5).

CDHS, California Department of Health Services. (2012). Economic and Health Effects of a State Cigarette Excise Tax Increase in California. eScholarship, University of California.

Ciapponi, A. (2011). Systematic review of the link between tobacco and poverty.

Chalak, A., Nakkash, R., Abu-Rmeileh, N. M., Khader, Y. S., Jawad, M., Mostafa, A., ... & Salloum, R. G. (2021). Own-price and cross-price elasticities of demand for cigarettes and waterpipe tobacco in three Eastern Mediterranean countries: A volumetric choice experiment. Tobacco Control.

Chaloupka, F. J., Straif, K., & Leon, M. E. (2011). Effectiveness of tax and price policies in tobacco control. Tobacco control, 20(3), 235-238.

Chaloupka, F. J., Yurekli, A., & Fong, G. T. (2012). Tobacco taxes as a tobacco control strategy. Tobacco control, 21(2), 172-180.

Christophi, C. A., Kolokotroni, O., Alpert, H. R., Warren, C. W., Jones, N. R., Demokritou, P., & Connolly, G. N. (2008). Prevalence and social environment of cigarette smoking in Cyprus youth. BMC Public Health, 8(1), 1-9.

Delipalla, S. (2009). Tobacco tax structure and smuggling. FinanzArchiv/Public Finance Analysis, 93-104.

Damiri, B. R., Isra'A, S., & Aghbar, M. H. (2018). Pattern of substance use among schoolchildren in Palestine: a cross-sectional study. Egyptian Journal of Forensic Sciences, 8(1), 1-10.

Doll, R., Peto, R., Boreham, J., & Sutherland, I. (2004). Mortality in relation to smoking: 50 years' observations on male British doctors. Bmj, 328(7455), 1519.

Donaldson, E. A., Waters, H. R., Arora, M., Varghese, B., Dave, P., & Modi, B. (2011). A cost-effectiveness analysis of India's 2008 prohibition of smoking in public places in Gujarat. International journal of environmental research and public health, 8(5), 1271-1286.

Drovandi, A., Teague, P. A., Glass, B., & Malau-Aduli, B. (2019). A systematic review of the perceptions of adolescents on graphic health warnings and plain packaging of cigarettes. Systematic reviews, 8(1), 1-15.

Fayter, D., Main, C., Misso, K., Ogilvie, D., Petticrew, M., Sowden, A., et al., 2008. Population Tobacco Control Interventions and Their Effects on Social Inequalities in Smoking. Centre for Reviews and Dissemination, York.

Foley, K. L., & Balázs, P. (2010). Social will for tobacco control among the Hungarian public health workforce. Central European journal of public health, 18(1), 25.

Francis, D. B., Hall, M. G., Noar, S. M., Ribisl, K. M., & Brewer, N. T. (2017). Systematic review of measures used in pictorial cigarette pack warning experiments. Nicotine & Tobacco Research, 19(10), 1127-1137.

Fu, S. S., McFall, M., Saxon, A. J., Beckham, J. C., Carmody, T. P., Baker, D. G., & Joseph, A. M. (2007). Post-traumatic stress disorder and smoking: a systematic review. Nicotine & tobacco research, 9(11), 1071-1084.

Gao, K., Shi, X., & Wang, W. (2017). The life-course impact of smoking on hypertension, myocardial infarction and respiratory diseases. Scientific reports, 7(1), 1-7.

Glasser, A. M., & Roberts, M. E. (2021). Retailer density reduction approaches to tobacco control: a review. Health & place, 67, 102342.

Golden, S. D., Smith, M. H., Feighery, E. C., Roeseler, A., Rogers, T., & Ribisl, K. M. (2016). Beyond excise taxes: a systematic review of literature on non-tax policy approaches to raising tobacco product prices. Tobacco Control, 25(4), 377-385.

Goodman, P., Agnew, M., McCaffrey, M., Paul, G., & Clancy, L. (2007). Effects of the Irish smoking ban on respiratory health of bar workers and air quality in Dublin pubs. American journal of respiratory and critical care medicine, 175(8), 840-845.

Guindon, G. E., Perucic, A. M., & Boisclair, D. (2004). Higher Tobacco Prices and Taxes in South East Asia: An Effective Tool to Reduce Tobacco Use, Save Lives and Generate Revenue.

Habibi, M., Hosseini, F., Darharaj, M., Moghadamzadeh, A., Radfar, F., & Ghaffari, Y. (2018). Attachment style, perceived loneliness, and psychological well-being in smoking and non-smoking university students. The Journal of psychology, 152(4), 226-236.

Hahn, E. J. (2010). Smokefree legislation: a review of health and economic outcomes research. American journal of preventive medicine, 39(6), S66-S76.

Hammond, D. (2011). Health warning messages on tobacco products: a review. Tobacco control, 20(5), 327-337.

Hernández, F. F., Leal, L. F., González, E. S., & Pérez, B. Z. (2021). The Touchable Smoking Social Costs. Journal of Clinical and Laboratory Research, 2(3).

Heydari, G., Talischi, F., Algouhmani, H., Lando, H. A., & Ebn Ahmady, A. (2013). WHO MPOWER tobacco control scores in the Eastern Mediterranean countries based on the 2011 report. EMHJ-Eastern Mediterranean Health Journal, 19 (4), 314-319, 2013.

Heydari, G., Ahmady, A. E., Lando, H. A., Shadmehr, M. B., & Fadaizadeh, L. (2014). The second study on WHO MPOWER tobacco control scores in Eastern Mediterranean Countries based on the 2013 report: improvements over two years. Archives of Iranian medicine, 17(9), 0-0.
Heydari, G., Ahmady, A. E., Lando, H. A., Shadmehr, M. B., & Fadaizadeh, L. (2014). The second study on WHO MPOWER tobacco control scores in Eastern Mediterranean Countries based on the 2013 report: improvements over two years. Archives of Iranian medicine, 17(9), 0-0.
Hoffman, S. J., & Tan, C. (2015). Overview of systematic reviews on the health-related effects of government tobacco control policies. BMC public health, 15(1), 1-11.

Hu, T. W., & Mao, Z. (2002). Economic analysis of tobacco and options for tobacco control: China case study.

Islam, F., Salloum, R. G., Nakkash, R., Maziak, W., & Thrasher, J. F. (2016). Effectiveness of health warnings for waterpipe tobacco smoking among college students. International journal of public health, 61(6), 709-715.

Jawad, M., Al-Houqani, M., Ali, R., El Sayed, Y., ElShahawy, O., Weitzman, M., & Sherman, S. E. (2019). Prevalence, attitudes, behaviours and policy evaluation of midwakh smoking among young people in the United Arab Emirates: Cross-sectional analysis of the Global Youth Tobacco Survey. PloS one, 14(4), e0215899.

Jawad, M., Jawad, S., Waziry, R. K., Ballout, R. A., & Akl, E. A. (2016). Interventions for waterpipe tobacco smoking prevention and cessation: a systematic review. Scientific reports, 6(1), 1-8.

John, R. M., Ross, H., & Blecher, E. (2012). Tobacco expenditure and its implications for household resource allocation in Cambodia. Tobacco control, 21(3), 341-346.

Joossens, L., & Raw, M. (1998). Cigarette smuggling in Europe: who really benefits?. Tobacco control, 7(1), 66-71.

Kabir, Z., Arora, M., & Alpert, H. R. (2011). Have public smoking bans resulted in an improvement in quality of life?. Expert review of pharmacoeconomics & outcomes research, 11(4), 375-377.

Kong, G., Creamer, M. R., Simon, P., Cavallo, D. A., Ross, J. C., Hinds, J. T., ... & Gutierrez, K. (2019). Systematic review of cigars, cigarillos, and little cigars among adolescents: Setting research agenda to inform tobacco control policy. Addictive behaviors, 96, 192-197.

Karekla, M., Symeou, A., Tsangari, H., Kapsou, M., & Constantinou, M. (2009). Smoking prevalence and tobacco exposure among adolescents in Cyprus. The European Journal of Public Health, 19(6), 655-661.

Leão, T., Kunst, A. E., & Perelman, J. (2018). Cost-effectiveness of tobacco control policies and programmes targeting adolescents: a systematic review. The European Journal of Public Health, 28(1), 39-43.

Leonardi-Bee, J., Jere, M. L., & Britton, J. (2011). Exposure to parental and sibling smoking and the risk of smoking uptake in childhood and adolescence: a systematic review and meta-analysis. Thorax, 66(10), 847-855.

Lochbuehler, K., Schuck, K., Otten, R., Ringlever, L., & Hiemstra, M. (2016). Parental smoking and smoking cognitions among youth: a systematic review of the literature. European addiction research, 22(4), 215-232.

Mamudu, H. M., Owusu, D., Asare, B., Williams, F., Asare, M., Oke, A., ... & Ouma, O. A. (2020). Support for smoke-free public places among adults in four countries in Sub-Saharan Africa. Nicotine and Tobacco Research, 22(12), 2141-2148.

Maraqa, B., Nazzal, Z., & Jabareen, J. (2020). Smoking Cessation Counseling: Attitude in the Background of Poor Practice Compliance among Palestinian Primary Health Care Physicians: A cross sectional study.

McKay, A. J., Patel, R. K., & Majeed, A. (2015). Strategies for tobacco control in India: a systematic review. PLoS One, 10(4), e0122610.

McKelvey, K., Popova, L., Kim, M., Chaffee, B. W., Vijayaraghavan, M., Ling, P., & Halpern-Felsher, B. (2018). Heated tobacco products likely appeal to adolescents and young adults. Tobacco Control, 27(Suppl 1), s41-s47.

McNeill, A., Brose, L. S., Calder, R., Bauld, L., & Robson, D. (2018). Evidence review of e-cigarettes and heated tobacco products 2018. A report commissioned by public health England. London: Public Health England, 6.

Meyers, D. G., Neuberger, J. S., & He, J. (2009). Cardiovascular effect of bans on smoking in public places: a systematic review and meta-analysis. Journal of the American College of Cardiology, 54(14), 1249-1255.

MoH, Ministry of Health. (2021). Annual health reports 2020. Palestinian health information center.

Mohamed, S. F., Juma, P., Asiki, G., & Kyobutungi, C. (2018). Facilitators and barriers in the formulation and implementation of tobacco control policies in Kenya: a qualitative study. BMC Public Health, 18(1), 1-14.

Nakkash, R., & Khalil, J. (2010). Health warning labelling practices on narghile (shisha, hookah) waterpipe tobacco products and related accessories. Tobacco control, 19(3), 235-239.

Nakkash, R., Khader, Y., Chalak, A., Abla, R., Abu-Rmeileh, A., Mostafa, A., … & Salloum, R. (2022). Prevalence of Cigarette and Waterpipe Tobacco Smoking among Adults in Three Eastern Mediterranean Countries.

National Cancer Institute. (2008) The Role of the Media in Promoting and Reducing Tobacco Use, Tobacco Control Monograph No. 19,U.S. Department of Health and Human Services, National Institutes of Health, National Cancer Institute, Bethesda, Md, USA.

Nazir, M. A., Al-Ansari, A., Abbasi, N., & Almas, K. (2019). Global Prevalence of Tobacco Use in Adolescents and Its Adverse Oral Health Consequences. Open access Macedonian journal of medical sciences, 7(21), 3659.

Nichter, M., Çarkoğlu, A., Nichter, M., Özcan, Ş., & Uysal, M. A. (2018). Engaging nurses in smoking cessation: Challenges and opportunities in Turkey. Health Policy, 122(2), 192-197.

Nishio, A., Saito, J., Tomokawa, S., Kobayashi, J., Makino, Y., Akiyama, T., ... & Yamamoto, M. (2018). Systematic review of school tobacco prevention programs in African countries from 2000 to 2016. PloS one, 13(2), e0192489.

Oncken, C. A., Dietz, P. M., Tong, V. T., Belizán, J. M., Tolosa, J. E., Berghella, V., ... & Bloch, M. H. (2010). Prenatal tobacco prevention and cessation interventions for women in low- and middle-income countries. Acta obstetricia et gynecologica Scandinavica, 89(4), 442-453.

PCBS, Palestinian Central Bureau of Statistics, and MOH, Ministry of Health, 2020. Palestinian Health Accounts 2000- 2018: Ramallah - Palestine.

PCBS, Palestinian Central Bureau of Statistics, and MOH, Ministry of Health, 2012. A Press Release on the occasion of International Day of Giving up Smoking (Word No Tobacco Day).

Palestinian Legislative Council. (2005). Public Health Law. (Trans. by WHO office for West Bank and Gaza Strip).

Parascandola, M., & Xiao, L. (2019). Tobacco and the lung cancer epidemic in China. Translational lung cancer research, 8(Suppl 1), S21.

Prasetyoputra, P., & Irianti, S. (2014). Health and economic implications of smoking in Indonesia: A review of the literature. Indonesian Journal of Health Ecology, 13(4), 340-353.

Reddy, Y. S. R. K. (2021). Effectiveness Of Structured Teaching Programme On Knowledge Regarding Adverse Effects Of Tobacco Usage Among B. Sc Nursing Students Of Sree Balaji College Of Nursing, Chrompet–Chennai. European Journal of Molecular & Clinical Medicine, 7(7), 6075-6079.

Rizzo, J. A. (2001). The labor productivity costs of smoking in the United States. Economic Analysis of Substance Use and Abuse: The Experience of Developed Countries and Lessons for Developing Countries, 181-202.

Salloum, R. G., Abu-Rmeileh, N., Hamadeh, R., Thomas, J., Mostafa, A., Yusufali, A., ... & Nakkash, R. (2017). Policy-Relevant context of waterpipe tobacco smoking among university students in six countries across the Eastern Mediterranean Region: a qualitative study. Asian Pacific journal of cancer prevention: APJCP, 18(9), 2533.

Salloum, R. G., Asfar, T., & Maziak, W. (2016). Toward a regulatory framework for the waterpipe. American journal of public health, 106(10), 1773-1777.

Salti, N., Brouwer, E., & Verguet, S. (2016). The health, financial and distributional consequences of increases in the tobacco excise tax among smokers in Lebanon. Social Science & Medicine, 170, 161-169.

Santoso, D. B., & Erlando, A. (2020). Tobacco excise tax policy in Indonesia: who does reap the benefits?. Entrepreneurship and Sustainability Issues, 8(1), 1108.

Scollo, M., Lal, A., Hyland, A., & Glantz, S. (2003). Review of the quality of studies on the economic effects of smoke-free policies on the hospitality industry. Tobacco control, 12(1), 13-20.

Smith, K. E., Savell, E., & Gilmore, A. B. (2013). What is known about tobacco industry efforts to influence tobacco tax? A systematic review of empirical studies. Tobacco control, 22(2), e1-e1.

Tee, G. H., Hairi, N. N., Nordin, F., Choo, W. Y., Chan, Y. Y., Kaur, G., ... & Bulgiba, A. (2015). Systematic review on international practices in controlling waterpipe tobacco smoking. Asian Pacific Journal of Cancer Prevention, 16(9), 3659-3665.

Thorberg, F. A., & Lyvers, M. (2006). Negative mood regulation (NMR) expectancies, mood, and affect intensity among clients in substance disorder treatment facilities. Addictive behaviors, 31(5), 811-820.

Ünübol, H., & Sayar, G. H. (2019). Psychological factors associated with smoking and quitting: addiction map of Turkey study. Neuropsychiatric disease and treatment, 15, 1971.

Usmanova, G., & Mokdad, A. H. (2013). Results of the Global Youth Tobacco Survey and implementation of the WHO framework convention on tobacco control in the WHO Eastern Mediterranean Region (EMR) countries. Journal of epidemiology and global health, 3(4), 217-234.

van Baal, P. H., Brouwer, W. B., Hoogenveen, R. T., & Feenstra, T. L. (2007). Increasing tobacco taxes: a cheap tool to increase public health. Health Policy, 82(2), 142-152.

Vansickel, A. R., Shihadeh, A., & Eissenberg, T. (2012). Waterpipe tobacco products: nicotine labelling versus nicotine delivery. Tobacco control, 21(3), 377-379.

Vardavas, C. I., Filippidis, F. T., Agaku, I., Mytaras, V., Bertic, M., Connolly, G. N., ... & Behrakis, P. (2012). Tobacco taxation: the importance of earmarking the revenue to health care and tobacco control. Tobacco induced diseases, 10(1), 1-5.

Veeranki, S. P., Alzyoud, S., Dierking, L., Kheriallah, K., Mzayek, F., Pbert, L., & Ward, K. D. (2016). Associations of adolescents' cigarette, waterpipe, and dual tobacco use with parental tobacco use. Nicotine & Tobacco Research, 18(5), 879-884.

Vodopivec-Jamsek, V., de Jongh, T., Gurol-Urganci, I., Atun, R., & Car, J. (2012). Mobile phone messaging for preventive health care. Cochrane Database of Systematic Reviews, (12).

Wakefield, M., Flay, B., Nichter, M., & Giovino, G. (2003). Role of the media in influencing trajectories of youth smoking. Addiction, 98, 79-103.

Wang, H., Sindelar, J. L., & Busch, S. H. (2006). The impact of tobacco expenditure on household consumption patterns in rural China. Social science & medicine, 62(6), 1414-1426.

Warner, K. E. (2000). The economics of tobacco: myths and realities. Tobacco control, 9(1), 78-89.

West, R. (2017). Tobacco smoking: Health impact, prevalence, correlates and interventions. Psychology & health, 32(8), 1018-1036.

White, V. M., Guerin, N., Williams, T., & Wakefield, M. A. (2019). Long-term impact of plain packaging of cigarettes with larger graphic health warnings: findings from cross-sectional surveys of Australian adolescents between 2011 and 2017. Tobacco control, 28(e1), e77-e84.

WHO, World Health Organization. (2010). WHO technical manual on tobacco tax administration. World Health Organization.

WHO, World Health Organization. (2011). Palestine STEPS Survey 2010–2011.

WHO, World Health Organization. (2015a). WHO report on the global tobacco epidemic, 2015. Country profile: West Bank and Gaza Strip.

WHO, World Health Organization. (2015b). Effects of meeting MPOWER requirements on smoking rates and smoking-attributable deaths: Palestine.

WHO, World Health Organization. (2015c). WHO report on the global tobacco epidemic 2015: raising taxes on tobacco. World Health Organization.

WHO, World Health Organization. (2019). WHO report on the global tobacco epidemic, 2019: occupied Palestinian territory, including east Jerusalem.

Wilson, L. M., Avila Tang, E., Chander, G., Hutton, H. E., Odelola, O. A., Elf, J. L., ... & Apelberg, B. J. (2012). Impact of tobacco control interventions on smoking initiation, cessation, and prevalence: a systematic review. Journal of environmental and public health, 2012.

Wright, A., Smith, K. E., & Hellowell, M. (2017). Policy lessons from health taxes: a systematic review of empirical studies. BMC public health, 17(1), 1-14.

Document 4

Policy Brief

Reforming waterpipe tobacco tax structure in Egypt:
Why and what can be done?
Towards Egypt's Vision 2030-improving health and economy for Egyptians

Dr. Aya Mostafa Kamal Eldin, MD, PhD
Professor of Public Health & Epidemiology
Faculty of Medicine, Ain Shams University, Cairo, Egypt
E-mail: aya.kamaleldin@med.asu.edu.eg

Contents

**Key Messages**	**4**
**Policy Brief**	**7**
The Problem	7
Size of the problem	8
Underlying Factors	11
Elements of a policy approach to address the problem	18
Element 1	21
Element 2	22
Element 3	25
Implementation considerations and counterstrategies	28
Next Steps	38
**References**	**40**
**Glossary**	**48**

Funding. This policy brief is a product of the "Eastern Mediterranean Consortium on the Economics of Waterpipe Tobacco Smoking (ECON-WTS)" funded by IDRC and support was provided through the Knowledge to Policy (K2P) Center for the development of the policy brief.

Key messages

What is the problem?

Waterpipe tobacco smoking constitutes a major public health and economic burden on the Egyptian population. The practice is still growing though several interventions have been implemented to control it, therefore, improved taxation would be an alternative approach to its control. Despite the evidence on its growing burden, the existing waterpipe tobacco taxation policy has gaps and requires restructuring to reduce waterpipe tobacco use prevalence and increase government revenues, hence, meet both the United Nations Sustainable Development Goals and Egypt's vision 2030 Goals.

What do we know about three elements of an approach to addressing the problem?

Element 1 — Establish a public platform and written policies for providing national data on waterpipe tobacco economics in Egypt

- ➔ An effective tax policy administration relies primarily on collection of comprehensive and reliable data.
- ➔ For obtaining accurate information, a good information technology system is required for periodic tax declarations, accounting, inventory, and financial data.
- ➔ As the evidence has previously shown in the case of modelling the impact of cigarette fiscal policies and quantifying its health and economic benefits, similar data would also be important to enable producing similar evidence for waterpipe tobacco tax models. Needed national data include: waterpipe tobacco sales volume, tobacco companies market shares, importers, distributers, governmental revenues from waterpipe tobacco taxation. In addition, information on national expenditure on discount, middle, and premium waterpipe tobacco products, home and café expenditure, the variety of brands (domestic and imported), flavors, and different weights of waterpipe tobacco packs are needed. Also, own- and cross-price elasticity of waterpipe tobacco products and service categories are required.
- ➔ Adoption of a written policy for publicly sharing, using, and regular reporting of data on waterpipe tobacco economics is necessary to ensure sustained commitment and responsibility of the government for public information sharing and transparency and the wide use of the provided data for the public benefit.

Element 2 — Producing policy-guiding knowledge by modelling the impact of fiscal policies on waterpipe tobacco use prevalence and government revenues

- ➔ Economic modelling for tobacco taxation is recommended by utilizing data on national waterpipe tobacco economics that will be made available through the official public platforms to model the impact of fiscal policies based on country-specific information
- ➔ The economic model would follow previously published models in this field.
- ➔ The Eastern Mediterranean Consortium on the Economics of Waterpipe Tobacco Smoking produced preliminary results of an economic model for waterpipe tobacco tax scenarios for Egypt, Jordan, Lebanon, and Palestine that demonstrate the health and economic benefits of adding specific excise taxes to the current waterpipe tobacco structure

so that 75% of the total retail price constitute tax, as recommended by the World Health Organization (WHO).

- ➔ For Egypt updated national data are urgently needed to enable an accurate simulation.
- ➔ Calculation of the health and economic benefits of recommended fiscal policies via a waterpipe tobacco tax system reform will enable policymakers to select the most efficient scenario that will lead to the maximum health and economic benefits.

Element 3 — Improving current waterpipe tobacco taxation system through raising its level and reforming its structure, guided by the modeling in Element 2

- ➔ Using the available data in Egypt to-date, preliminary results of modeling suggest that raising the waterpipe tobacco tax level adherent to the WHO FCTC guidelines would reduce waterpipe tobacco smoking by 50%, increase governmental revenue three-fold, and substantially reduce the associated premature deaths relative to the base case scenario. These results are subject to further verification and sensitivity analysis.
- ➔ Conduct modifications to the waterpipe tobacco tax structure by including a specific excise component and a uniform one-tiered structure using the evidence generated from country-specific models and the recommended WHO FCTC Article 6 guidelines.
- ➔ A specific excise component leads to higher prices and a lower market share of cheap products than an Ad valorem excise tax. In an ad valorem system, a minimum specific tax should also be implemented to guarantee minimum price and revenue levels [44].
- ➔ The WHO recommends including a specific excise component within a simple and uniform tax structure as a superior approach to an ad valorem and tiered tax structure in reducing tobacco consumption. This approach may result in by 6-65% and leads to larger reductions in smoking as there is less opportunity to switch between different tiers and types of tobacco products.

Implementation considerations

To enhance the control of the waterpipe tobacco epidemic in Egypt through an improved waterpipe tobacco taxation policy, a variety of implementation considerations need to be kept in mind at the levels of consumers, professionals, organizations, and systems.
ef

Policy Brief
April 2022

Reforming waterpipe tobacco tax structure in Egypt: Why and what can be done?

I. The problem

Egypt is one of the largest waterpipe tobacco markets [1] and is one of only six countries worldwide where tobacco use is rising [2].More than a fifth of Egyptian high school [3] and a quarter of university students reported waterpipe tobacco smoking (WTS) [4]. The latest national surveys revealed that Egyptian adolescents reported higher WTS rates relative to adults [5,6]. Annually, more than 170,000 tobacco-attributable deaths occur and USD 616 million are spent in treating tobacco-related diseases in Egypt [7].

This health and economic burden is partly due to the WTS-associated diseases such as cancers; respiratory, and cardio-vascular diseases [8]. In addition, WTS causes tobacco/nicotine dependance [9] and exposes smokers and surrounding people to dangerous toxicants [10]. Moreover, WTS potentiates cigarette smoking and multiple tobacco product use [11].

Smoking cessation reduces the risk of premature death and can add as much as a decade to life expectancy [12]. Failing to reduce WTS through ensuring reduced affordability of waterpipe tobacco via restructuring waterpipe tobacco tax in Egypt may deter the achievement of the Sustainable Development Goals (SDGs), specifically SDG target 3.a by 2030 [13], and Egypt's vision 2030 [14].

Background to Policy Brief
A Policy Brief brings together global research evidence, local evidence and context-specific knowledge to inform deliberations about health policies and programs. It is prepared by synthesizing and contextualizing the best available evidence about the problem and viable solutions and options through the involvement of content experts, policymakers, and stakeholders.

The preparation of the Policy Brief involved the following steps:
1) Selecting a priority topic according to Knowledge to Policy Center criteria.
2) Selecting a working team who deliberates to develop an outline for the policy brief and oversee the litmus testing phase.
3) Developing and refining the outline, particularly the framing of the problem and the viable options
4) Litmus testing by conducting one-to-one interviews with up to 15 selected policymakers and stakeholders to frame the problem and make sure all aspects are addressed.
5) Identifying, appraising, and synthesizing relevant research evidence about the problem, options, and implementation considerations
6) Drafting the brief in such a way as to present concisely and in accessible language the global and local research evidence.
7) Undergoing merit review
8) Finalizing the Policy Brief based on the input of merit reviewers, translating into Arabic, validating translation, and disseminating through policy dialogues and other mechanisms.

- **Economic benefits derived from ear marked tobacco taxation are reinvested in health sector development through universal health coverage [16-18].**

- **Approximately 75% of governmental revenues are from taxes [15,49,50]; tobacco taxes constitute 9% of this amount [49-51].**

II. Size of the problem

Although cigarette smoking rates are declining in developed countries, WTS prevalence is increasing globally and in the Eastern Mediterranean Region, particularly among youth [19]. Frequent calls were made for improved and comprehensive WTS regulations to prevent and control its use [20]. Tobacco control represents a major challenge in Egypt with 22.8% of Egyptian adults currently using tobacco products [6]. Approximately, 50% of Egyptians are exposed to secondhand smoke in their homes [6]. Trends in uptake of tobacco among young Egyptian females are increasing, with an overall rise in WTS in Egypt [2,21,22]. Tobacco use is a driving force in the rising epidemic of chronic diseases in Egypt, such as lung disease, lung cancer, ischaemic heart disease and stroke [6]. The latest national estimates of prevalence of current tobacco use in Egypt including cigarettes, waterpipe tobacco, and other tobacco products is presented in **Table 1.**

Table 1. Prevalence of current tobacco use in Egypt including cigarettes, waterpipe tobacco, and other tobacco products [5,6]

	**Youth** [5]	**Adults** [6]
**Male**	18.1%	43.6%
**Female**	8.2%	0.5%
**Total**	13.6%	22.8%

II.1 Prevalence and socio-demographic profile of Egyptian waterpipe tobacco smokers

The overall prevalence of WTS has increased from 3.3% in 2009 [23] to 4.5% in 2017/2018 [6] and has at least tripled among men aged under 45 years [6,23]. The latest national prevalence of current WTS was higher among men (8.7%) than among women (0.1%) **(Figure 1)** [6]. Among men, WTS prevalence was higher in those above 45 years old than in younger age groups [6]. However, in females WTS prevalence was higher among the age group 15-24 years old [6]. In young Egyptian adults (17-25 years old), the overall prevalence of WTS was 12.2% (21.6% in males and 0.9% in females) [4].

Noticeably, current WTS among adolescent girls is several folds higher (4.1%) **(Figure 1)** [5] than their older counterparts (0.9% and 0.1%) [4,6]. Three-quarters of WTS households were located in rural areas [24]. WTS was five to ten times more prevalent among individuals of lower income levels [24], twice as higher in rural than in urban Egypt [23], and three-folds higher in waterpipe tobacco smokers with no formal education than those with university education [23].

Figure 1. Prevalence of waterpipe tobacco smoking in Egypt [5,6]

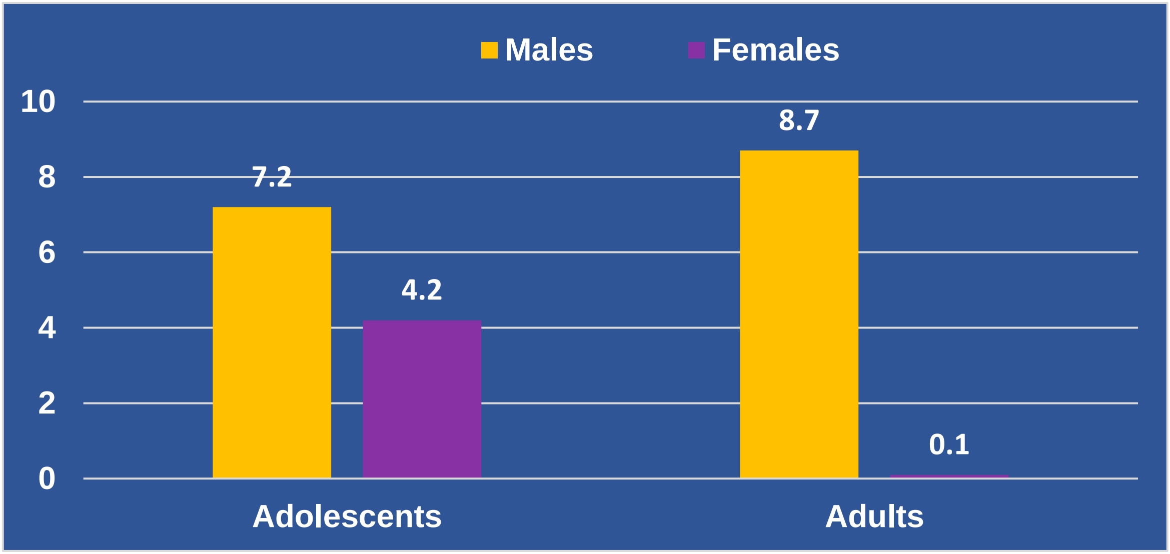

II.2 Patterns of WTS in Egypt

Frequency: More than two-thirds of waterpipe tobacco smokers smoked it daily [6]. Among daily waterpipe tobacco smokers, the average number of WTS sessions per day was 3.6 and the average number of tobacco portions "hagar/head/ras"* smoked per session was 2.8 [6], which translates to 10.1 hagar per day in most waterpipe tobacco smokers. ***Place:*** More than half of (51.6%) smoked waterpipe tobacco at homes, and 45.1% smoked it at cafes [6]. ***Price:*** The average monthly expenditure on WTS among university students was EGP 209 [4]; current and daily shisha smokers, spent from more than one-third (37.8%) to more than two-fifths (44.7%) of their monthly pocket money on WTS, respectively [4]. WTS individuals and households spent approximately 1200-1800 EGP annually on its use, representing 3.7% of their annual household income [9,24]. ***Type:*** Most of the waterpipe tobacco smoked was flavorless; 8.6% smoked only flavored waterpipe tobacco; and 2.4% smoked a mix of both flavored and flavorless waterpipe tobacco [6]. ***Harm perceptions:*** Only 16.4% of them believed that WTS contains more nicotine than cigarettes [9]. ***Dependance and multiple use:*** More than half of the WTS university students made at least one quit attempt to quit WTS during the past 12 months prior to the study [4]. Nearly half (47.9%) of the individuals who smoked waterpipe tobacco smoked multiple tobacco products [9], and 25.8% of the waterpipe tobacco smokers considered themselves addicted to WTS [9]. Multiple product use was more common in young adult female than in young adult males and older females [22]. ***Social acceptability:*** More than two-thirds of male versus less than a third of female university students perceived WTS as socially acceptable [4]. However, most students (90.6% and 94.8% of males and females, respectively) perceived WTS for females as socially unacceptable [4]. Seeking social allure and being fashionable was one of the elements that attributed to the rise of WTS among females in Egypt [26-28].

---

* The waterpipe tobacco portion used in a WTS session is called "head/hagar/ras" and one portion is equivalent to approximately 20g of waterpipe tobacco [25].

II.3 Health, economic, and environmental burden of WTS in Egypt

Tobacco use in Egypt contributes to a significant number of premature deaths, healthcare costs and disability [7]. Yet, the specific share of WTS in this burden is not clear.

II.3.1 Health burden: Most WTS smokers are unaware that WTS exposes them to dangerous toxicants (carbon monoxide, volatile aldehydes, polycyclic aromatic hydrocarbons, and nitrosamines), heavy metals (cadmium and lead), and the dependence-producing drug, nicotine like those in cigarettes [10]. A systematic review of WTS effects on health outcomes reported that WTS is associated with a number of diseases, such as respiratory diseases, oral cancer, lung cancer, low birthweight, metabolic syndrome, cardiovascular diseases and adverse mental health [8]. A study conducted among young adult smokers in Egypt, Jordan, Morocco, and Oman reported that relative to their non-smoking counterparts, habitual waterpipe tobacco smokers exhibited a significant burden of respiratory symptoms that was comparable to that observed with cigarette smokers [29]. Also, some studies reported the association of WTS with infectious diseases such as Tuberculosis [30] and COVID-19 [31-34], which is related to the social nature of smoking waterpipe tobacco in groups and sharing the device and its accessories; a practice that is not uncommon (30%) in Egypt [9]. Also, some reports documented that positive bacterial cultures were isolated from the waterpipe apparatus [35]. A study in 2010 estimated that a 70% increase in cigarette taxes would raise revenues and prevent more than 600,000 premature deaths [7]. A similar projection of the associated health benefits from raising waterpipe tobacco taxes is urgently needed.

Nevertheless, key findings from the 2020 Surgeon General's Report indicate that smoking cessation enhances quality of life, reduces the risk of premature death, and can add as much as a decade to life expectancy [12]. While these findings were reported mainly for cigarette smoking, they may also apply to WTS given the similarity in their toxicant constituents and the adverse health events associated with their use. For instance, , a waterpipe tobacco smoker smokes on average several hagar per session, and smokes 2–3 sessions per day [25]. This translates into an intake of nicotine equivalent to more than one pack of cigarettes per WTS session for most waterpipe tobacco smokers [25]. Also, deep inhalation of smoke during WTS affects the lung tissue alveoli more than in cigarette smoking [25]. WTS produces more smoke than cigarette smoking [25]; smoke exposure was estimated to be as much as 100-200 cigarettes per WTS session [25]. A meta-analysis of waterpipe and cigarette toxicant exposure reported that one WTS session consistently exposed users to larger smoke volumes compared with one cigarette [36]; one WTS session was associated with 74.1 liters of smoke inhalation while one cigarette was associated with 0.6 liters of smoke [36]. It also reported that one WTS session was associated with higher levels of tobacco toxicants compared with one cigarette [36]: one WTS session would roughly be equivalent to 25 cigarettes worth of tar, 11 cigarettes worth of carbon monoxide, and 2 cigarettes worth of nicotine [36]. Research from other countries in the region support this evidence. For instance, studies from Jordan [37] and Lebanon [38] reported that toxicant content (total particulate matter, carbon monoxide, nicotine, tar, and formaldehyde) per WTS session is at least equal, but for many toxicants several magnitudes of order higher, than that of a cigarette [37,38].

II.3.2 Economic burden: In 2010, it was reported that EGP 3.4 billion (USD 616 million) were spent annually to treat tobacco-related diseases [7]. In the cost-of-illness study done in 2014, the

annual direct cost of inpatients in three hospitals was above EGP53 million for the four smoking-related diseases inpatients (chronic obstructive pulmonary disease, ischaemic heart disease, cancers of the respiratory system, and cerebrovascular stroke) [39]. The indirect mortality cost as years lost for employed people for the four diseases due to smoking was More than EGP 727 million [39]. The study noted the unavailability of national morbidity data for chronic diseases as limitation for calculating the national direct cost of the tobacco-related diseases [39]. Total waterpipe tobacco consumption in Egypt was estimated at about 50 thousand tonnes in 2019 [40]. However, based on STEPS 2017/2018 data, the national consumption of waterpipe tobacco would be at least 80 thousand tonnes among daily smokers, who represent approximately two-thirds of current waterpipe tobacco smokers in Egypt [6]. Official estimates of national rates of total expenditure on WTS are lacking, but were estimated by an expert as EGP 3 billion per year [40]. Household expenditure on waterpipe tobacco represented 3.7% of the total annual household income in 2017/2018 [24].

II.3.2 Environmental burden: To date, published local studies on the environmental burden of WTS in Egypt are scarce. Particulate matter under 2.5 μm in diameter (PM2.5) is one of the markers of air quality. It arises from diverse sources, including tobacco smoke from cigarettes and waterpipes, and is recognized as a cause of acute and chronic morbidity and mortality. One study measured PM2.5 levels via an air sampling study in a sample of 96 indoor and outdoor venues during 2005-2006 in Cairo, Egypt. Compared to indoor venues where tobacco smoking was banned (PM2.5 levels 72-81 μg/m(3)), places offering waterpipes to patrons of cafes (478 μg/m(3)) and Ramadan tents (612 μg/m(3)) had much higher concentrations, as did venues such as public buildings with poor enforcement of smoking restrictions (range 171-704 μg/m(3)). Both the number of waterpipe smokers and the number of cigarette smokers observed at each venue contributed significantly to the overall burden of PM2.5 [71].

III. The underlying factors

WTS is further exacerbated by the misperception of WTS harm and nicotine content relative to cigarette smoking, increased social acceptability of WTS, relative affordability of waterpipe tobacco compared with cigarettes, the weak enforcement of smoke-free policies, and the existing gaps in waterpipe tobacco regulations, specifically the non-uniform tobacco taxation [9,12,18,41]. It is considered a social practice and a tool for low cost entertainment [1]. The underlying factors for raising and restructuring waterpipe tobacco tax structure in Egypt could be discussed within the governance, finance, and delivery levels.

III.1. Governance level

At the governance level, the Ministry of Finance is responsible for tobacco taxation and is collaborating in this regard continuously with the Ministry of Health [17,42]. More specifically, the Egyptian Tax Authority is the entity that regulates and monitors the enforcement of the tobacco taxation regulations [17].

Tobacco taxation is the most cost-effective policy measure among those recommended by the **World Health Organization Framework Convention on Tobacco Control** (WHO FCTC) in reducing tobacco use while providing a reliable source of government revenues [18,43]. Tobacco

taxation is regulated by the ***Tobacco Control Law*** and is regularly updated on a nearly annual basis through Presidential Decrees and Circulars [17, 42]. Legislations in this regard are issued by the Ministry of Finance as a member of the Higher Committee for Tobacco Control and are normative and regulatory [17,42]. Article 2 of Law 2007/147, an amendment to Law 1981/52 (adding Article 6 repeated.4) [42] ensures that the Government is committed to a tax and price policy to increase the tobacco unit price as an effective mean to decrease tobacco use [17,42].

Moreover, Egypt signed and ratified international conventions that require adopting tobacco taxation as a powerful tool to reduce its affordability, hence reduce tobacco consumption [43]. Egypt has been a ***Party to the WHO FCTC*** since 2005 [41]. Article 6 guidelines of the Convention recommend that tobacco product taxes be [43,44]:

a) *Domestic not customs* because customs taxes are eroded in trade agreements and thus do not provide long-term revenue;
b) *Excise not general* because excise tax raises the price of the taxed good relative to all other goods, which discourages its consumption;
c) *Specific not ad valorem* because specific tax leads to higher prices and a lower market share of cheap cigarettes; thus reducing tobacco-related health inequalities. In an ad valorem system, a minimum specific tax should also be implemented to guarantee minimum price and revenue levels;
d) *Uniform not differential* because uniform tax leads to larger reductions in smoking as there is less opportunity to switch between different tiers and types of tobacco products;
e) *Comparable across all tobacco products* because similar levels of taxation across products reduce tobacco consumption, rather than simply leading to shifts in consumption between different tobacco products;
f) *No duty-free allowances* because duty-free sales increase tobacco consumption due to lower prices of products, and reduce tax revenue;
g) *Regularly increased because r*egular tax increases in line with gross domestic product ensure that the affordability and consumption of tobacco products decrease, and
h) *With tobacco tax revenue earmarked:* Ideally, tobacco tax revenue should be earmarked for particular health spending purposes, for example on tobacco control strategies.

Furthermore, Egypt has recently signed the ***Protocol to Eliminate Illicit Trade in Tobacco Products*** in January 2021 (Presidential Decree No. 170/2020), a measure recommended by WHO FCTC Article 15 and complements Article 6 [43,45]. Curbing illicit trade enhances the effectiveness of tobacco tax and price policies in reducing tobacco use and in achieving the public health and revenue goals of tobacco taxation [43].

The Ministry of Finance requires that all tobacco products carry a tax stamp that is screened via a ***banderole system*** as a cost-effective way to monitor tobacco production and movements, reduce non-compliance by tobacco manufacturers and distributors, and counter the illicit production and trade of tobacco products [46,47]. Also, the system is linked to a real-time electronic monitoring system database for tax authorities to record production/importation of various brands and other products that originate from factory premises or point of entry; thus, helping in tax calculations, verifying market trends, and is useful in reviewing tax policies on tobacco products [47]. This tracking and tracing system on tobacco products is one of the key provisions of the Protocol to

Eliminate Illicit Trade in Tobacco Products [43]. However, due to the presence of more than 40 waterpipe tobacco factories that are spread over Egypt and the informal operation of other production plants, monitoring the compliance of these producers with the banderole system and tax reporting is not strong and comprehensive as that for cigarettes [47]. Worryingly, the waterpipe tobacco industry, including waterpipe cafes, operates in an almost completely unregulated market and employs deceptive marketing techniques to attract new users [48].

Nonetheless, waterpipe tobacco-specific policies are not currently developed and implemented in a comprehensive approach and lack multi-sectoral coordination for smoke-free policies, cessation services addressing perceived self-efficacy and addiction, tobacco packaging and labelling, and education on WTS dependency and harms [9,41].

III.2. Finance level

The rationale for regular tobacco tax increases originated from the need of the Egyptian government to expand the health insurance programme coverage [17,47]. But given fiscal uncertainties and slow growth in the global economy, the government had to enhance its revenue base [17,47]. The revenue of this increase was directed to support health care services by an agreement between the Ministers of Health and Finance. Every change in the tax policy, the Ministry of Finance consults with the Ministry of Health first [17,42,47].

Tobacco tax is recognized as an important financial mean to enable countries to implement the SDGs including target 3.a [49]. **Earmarking of tobacco taxes:** In Egypt, a specific amount from taxes on cigarettes is earmarked to fund health care. In 1992, a specific amount was levied per stick to fund health insurance of students [18]. In July 2018, a new health insurance contribution of EGP 0.75 per pack was implemented [18]. Currently, student health insurance and new health insurance contribution per pack of cigarettes was 5.0% of its retail price (an extra 0.1 EGP per pack is levied to fund the students' health insurance and an additional 0.75 EGP per pack is levied to fund the national health insurance [41]. A recent report stated that 75% of revenues for Egypt were from taxes (total revenues for 2020/2021 were EGP 1.108 trillion and total tax revenues were EGP 834 billion) [15,50-52]. In the fiscal year 2020/2021, tobacco tax revenues were EGP 75 billion (USD 4.6 billion) constituting nearly 9% of total governmental revenues with an increase of EGP 10 billion than the previous fiscal year [52].

In 2009, the Ministry of Finance requested the collaboration with the World Health Organization regarding an economic model for cigarette taxation [17,47]. The plan was to introduce an excise system which would replace the general sales tax and introduce a value added tax on cigarettes [17,47]. In July 2010, Egypt moved from a tobacco tax system with eight tiers of specific excise tax based on the retail price of cigarettes, to a mixed excise system introducing a uniform specific excise tax of LE 1.25 per pack and an ad valorem excise of 40% on retail prices [18]. In 2014, three tiers of specific excise tax based on the retail price of a pack were introduced [18]. Ad valorem tax remained at a uniform 50% of the market price of cigarettes [18]. The amount of excise on each tier and the price definition of each tier have been increased regularly [18]. These improvements resulted in a 151% increase in revenues from EGP 7 to EGP 17.6 billion between 2010 and 2012, a 14% decrease in tobacco sales within two years, and a 46% increase in the cigarette tax per pack for the most popular brand [17,47]. The latest World Health Organization

estimate in 2020 for the total taxes as a percentage of the retail price of a pack most sold brand of cigarettes was 78.5% [41].

The latest amendment of the Egyptian tobacco tax regulations was in February 2020 [53]. To date, the waterpipe tobacco tax structure in Egypt does not comprise specific excise tax [41,53]. The ad valorem excise tax is tiered for domestic (165%) and imported (200%) tobacco [53]. Also, waterpipe tobacco is still not taxed as highly as cigarettes [18,41,53]. The latest World Health Organization estimate in 2020 for the total taxes as a percentage of the retail price of 20g of the most sold brand of waterpipe tobacco was 70.9% [41]. The tax structure of cigarettes and waterpipe tobacco according to the World Health Organization estimates in 2020 are presented in **Table 2**.

Table 2. The tax structure of cigarettes and waterpipe tobacco according to the World Health Organization estimates in 2020 [41]

	**Cigarettes (pack of 20)**	**Waterpipe tobacco (20 grams)**
Price of most sold brand in EGP	17.00	7.50
Price of most sold brand in USD	1.07	0.47
**Taxes on the most sold brand (% of retail price)**		
Total taxes	78.53%	70.93%
Specific excise	23.53%	0.00%
Ad valorem excise	50.00%	68.53%
Value added tax or sales tax	0.00%	0.00%
Import duty	0.00%	2.40%
Other taxes (Student health insurance and new health insurance contribution)	5.00%	0.00%

Changes in total tax levels on cigarettes and waterpipe tobacco from 2014 to 2020 in Egypt are presented in **Figure 2**.

Figure 2. Total taxes on cigarettes and waterpipe tobacco as a percentage of retail price in Egypt, 2014-2020 [54-57]

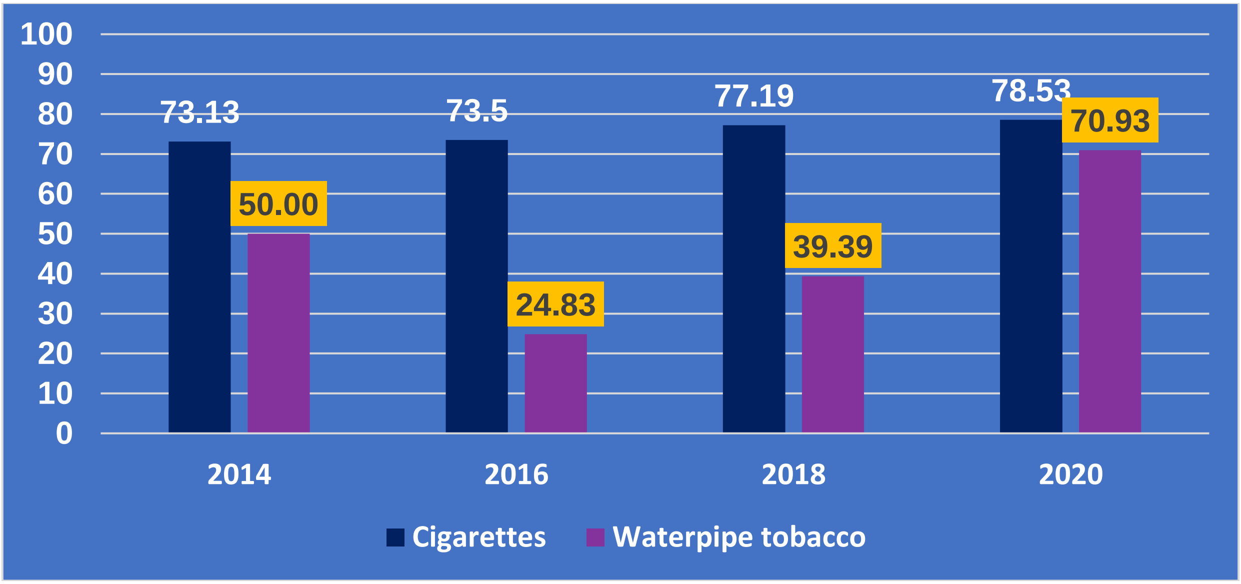

The changes in waterpipe tobacco tax level and structure since 2014 are presented in **Figure 3**.

Figure 3. Taxes on 20 gm of the most sold brand of waterpipe tobacco in Egypt, 2014-2020 [54-57]

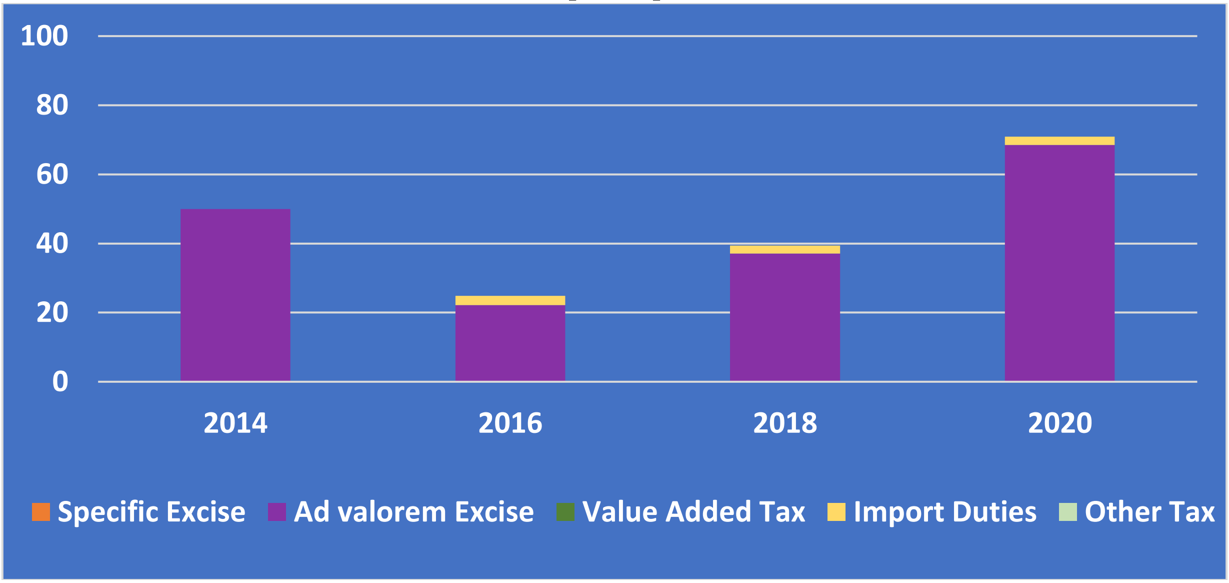

On the other hand, cigarette taxes have comprised a steady specific excise component over the past 7 years [18] **(Figure 4)**. Excise taxes are applied specifically to tobacco products and directly increase the retail price [58]. Higher prices dissuade people from considering or continuing a smoking habit [58].

Figure 4. Taxes on the most sold brand a pack of 20 cigarettes in Egypt, 2014-2020 [54-57]

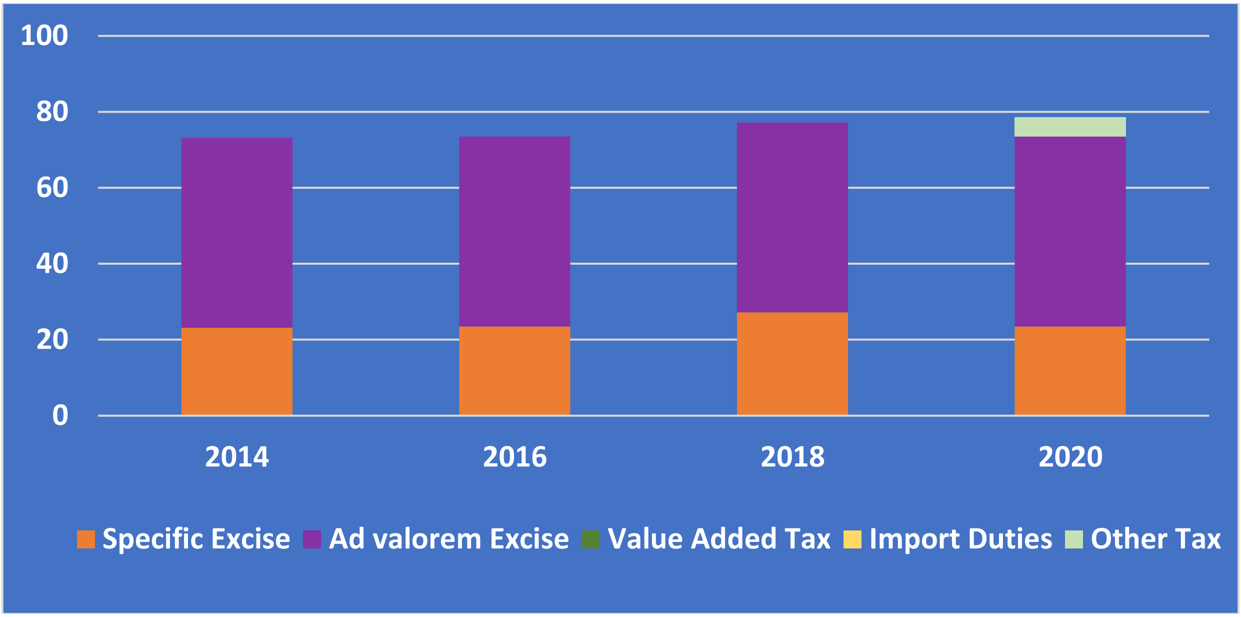

Table 3 describes how the price of the most sold brand of waterpipe tobacco is still more affordable (2.3 times) than cigarettes, which encourages product switching to the lower priced type of tobacco. Moreover, waterpipe tobacco is more affordable in Egypt than in other countries [18]. This is due to the differential tax structure that is not comparable across tobacco products.

Table 2. Price of the most sold brand of waterpipe tobacco and cigarettes in Egypt, 2014-2020 [54-57]

	2014	2016	2018	2020
**Price of 20 gm of the most sold brand of waterpipe tobacco**				
Price in USD	0.06	0.77	0.43	0.47
Price in EGP	0.4	6.80	7.70	7.50
**Price of a pack of 20 cigarettes of the most sold brand**				
Price in USD	0.57	1.13	0.9	1.07
Price in EGP	8.00	10.00	16.00	17.00

Although the 2017/2018 national STEPS survey reported the average amount spent on a pack of cigarettes among adults in Egypt (EGP 17.6) [6], national data on the average price of one waterpipe tobacco hagar and on the average daily spending on WTS were not collected from participants [6]. However, the Cairo and Menoufia survey in 2015-2017 reported that the average daily spending on WTS among 1490 current waterpipe tobacco smokers was EGP 10.8 and the average price of one waterpipe tobacco hagar was EGP 1.8 [unpublished data from reference 9]. In the latter survey, there was a gap in the reported prices and frequency of consumption between urban and rural areas. Prices were at least 5 times higher in urban than in rural areas mainly because urban smokers usually smoked waterpipe tobacco at a café, while rural smokers smoked mainly at home [unpublished data from reference 9]. This is even though rural smokers smoked larger amounts waterpipe tobacco daily (at least 2.5 times more than urban smokers) [unpublished data from reference 9].

Furthermore, the gap in prices between the two tobacco products in **Table 3** may be even wider if we consider the difference in the average amount of tobacco consumed in a WTS session as compared to cigarettes. More importantly, rural smokers are mostly of lower income levels [24] that are more vulnerable to catastrophic healthcare costs. The link between WTS and poverty is evident in its socio-demographic distribution in Egypt [23,24]. Therefore, restructuring waterpipe tax through adding a specific excise component to reduce the affordability of waterpipe tobacco may help in protecting these vulnerable populations.

III.3. Delivery level

Administrative requirements for the current waterpipe tobacco Ad valorem excise tax in Egypt requires a strong tax administration with technical capacity and manpower to conduct regular inspection and monitoring on production and distribution outlets [44,59]. Also, the Ad valorem excise tax may encourage consumer switching towards cheaper tobacco brands or products; thus, reducing the health benefit [44,59].

Incomplete data on market shares of tobacco companies, importers, distributers, especially via social media, in addition to the variety of brands (domestic and imported), flavors, and quantities of waterpipe tobacco packages makes enforcement and monitoring challenging for the administration [44,47,59]. The limited information in this regard is equally restrictive to researchers who need these data on a national basis for running policy-guiding predictive models. The Ministry of Finance has clear national data on cigarettes, but for waterpipe tobacco national data are not similarly up-to-date and published [47].

Choosing between specific and ad valorem taxes is still debatable in tax policy as both the tax level and tax structure of excises have different implications that can serve different interests and goals [44,59]. Accordingly, these two types of excise taxes may have different public health implications and may affect individual consumption via their impact on product quality, variety, and prices [44,58,59].

One factor is particularly important in delivery of tobacco tax, namely the ***tobacco-related health disparities***. Ad valorem excise tax is susceptible to undervaluation, and tends to lead to relatively lower prices [44,59]. This is particularly important as most smokers are of lower income categories and consume mostly low-priced brands [44,59]. While application of specific excise tax leads to relatively higher prices, particularly for low-priced cigarettes. Additionally, the specific excise tax will discourage consumption of tobacco products irrespective of the price band [44,59].

Moreover, the ***lack of supporting evidence and economic models*** that study which type of excise application will result in targeted public health benefits or higher governmental revenues adds to the challenge facing policy makers on which type to choose or a mix to levy and at what rate [44,59]. Also, there is no local evidence on own-price elasticity and cross-price elasticity of demand for waterpipe tobacco products.

Furthermore, there are ***particularities related to WTS*** that may make enforcement of waterpipe tobacco tax regulation challenging. WTS is used as a tool to socialize; one customer-product interface is the commercial waterpipe premises (waterpipe café) [1]. Users pay for both a product

(waterpipe tobacco) & the service (having it prepared by the staff) [1]. Current tobacco regulations focus more on cigarettes, overlooking the fact that businesses may dedicate their premises to the sale & onsite consumption of profitable non-cigarette tobacco products. Turnover from the sale of profitable waterpipe tobacco at cafes often outweighs the financial disincentive to adhere to tobacco control & other business laws. Also, current regulations come from multiple and different government departments, making the coordination of enforcement inefficient [17,47].

Elements of a policy approach to address the problem

The following three elements form a comprehensive approach for an improved waterpipe tobacco taxation policy.

Element 1 – Establishing a public platform and written policies for providing national data on waterpipe tobacco economics in Egypt

Element 2 – Producing policy-guiding knowledge by modelling the impact of fiscal policies on waterpipe tobacco use prevalence and government revenues

Element 3 – Improving current waterpipe tobacco taxation system through raising its level and reforming its structure , guided by modeling in Element

Policy elements to address the problem

Three suggested elements are proposed to address raising and reforming waterpipe tobacco tax structure in Egypt.

SUMMARY

Element 1

Establishing a public platform and written policies for providing national data on waterpipe tobacco economics in Egypt

Element 2

Producing policy-guiding knowledge by modelling the impact of fiscal policies on waterpipe tobacco use prevalence and government revenues

Element 3

Improving current waterpipe tobacco taxation system through raising its level and reforming its structure, guided by the modeling in Element 2

The methodology for formulation of elements consisted three phases of examining available reports, peer reviewed manuscripts, grey literature, policy briefs, expert views, and unpublished reports. The available evidence was carefully reviewed to produce this document. The databases used in this search included PubMed and Health System Evidence using the search terms: “waterpipe”; “tobacco”; “taxation”; “regulations”; “guidelines”; “burden”. Articles/documents published in English and Arabic were included with no restriction on the search period. Last search was done in September 2021. Evidence reviewed was segregated into themes. Themes included: data availability, improved taxation policy tools, knowledge production through modelling, and best practices in tobacco tax implementation. Through this methodology, a total of 42 articles were reviewed.

In the following phase, exploring the identified themes and mapping them with the problems and gaps identified through the exploration of the local context. As a result of this mapping process, three elements of a solution were formulated based on the relevant themes identified.

In the last phase, fine searches addressing the formulated elements and their components were conducted. Articles/documents published in English and Arabic were included with no restriction on the search period. Last search was done in March 2022. As a result of this fine search, 28 additional articles/documents were reviewed.

Through this triphasic methodology, a total of 70 articles/documents were reviewed, with the retrieval of 18 relevant articles used in the synthesis of elements. These articles/documents consisted of 6 systematic reviews and 12 individual studies/reports. Most of the retrieved studies were conducted on cigarettes. Results on benefits, harms, cost, cost-effectiveness, and uncertainty of interventions are presented below. The stakeholders interviewed during preparation of this policy brief commended the proposed elements.

IV.1 Element 1 – Establishing a public platform and written policies for providing national data on waterpipe tobacco economics in Egypt

1.1 Identify the national data on waterpipe tobacco economics to be publicly reported

The review of evidence in the WHO technical manual on tobacco tax policy and administration indicates that effective tax policy administration relies primarily on collection of comprehensive and reliable data [44]. A good information technology system is required for periodic tax declarations, accounting, inventory, and financial data is critical for obtaining accurate information [44].

As the evidence has previously shown in the case of modelling the impact of cigarette fiscal policies and quantifying the health and economic benefits [60,61], similar data would also be important to enable producing similar evidence for waterpipe tobacco tax models. Needed national data include: waterpipe tobacco sales volume, tobacco companies market shares, importers, distributers, governmental revenues from waterpipe tobacco taxation. In addition, information on national expenditure on discount, middle, and premium waterpipe tobacco products, home and café expenditure, the variety of brands (domestic and imported), flavors, and different weights of waterpipe tobacco packs are needed. Also, own-price elasticity data is absolutely essential for modeling the health and economic impacts of waterpipe tobacco taxation, while cross-price elasticity data is highly desirable [62].

1.2 Develop and implement written policies and mechanisms for using, sharing, and reporting the publicly provided data

To ensure sustained commitment from both the governmental side to provide regular and updated data and the public side to use the provided waterpipe tobacco economics data wisely, it would be necessary to develop a written policy that details the responsibilities of the Ministry of Finance for public information sharing and transparency [63].

Table 4. Key findings from systematic reviews and single studies

Category of finding	Element 1
Benefits	A systematic review revealed that effective tax policy administration relies primarily on collection of comprehensive and reliable data [44]. A good information technology system is required for periodic tax declarations, accounting, inventory, and financial data is critical for obtaining accurate information [44].
	Two studies demonstrated the use of publicly reported national data in the case of modelling the impact of cigarette fiscal policies and quantifying the health and economic benefits [60,61].
Harms	Literature review did not reveal any potential direct harms
Cost and/ or cost effectiveness in relation to the status quo	There were no studies available in the literature about the direct cost of the taxation intervention. However, there were studies that detailed the needs of the administration to apply such policy.<br><br>One study demonstrated that the needed national data for waterpipe economic modeling include: waterpipe tobacco sales volume, tobacco companies market shares, importers, distributers, governmental revenues from waterpipe tobacco taxation. In addition, information on national expenditure on discount, middle, and premium waterpipe tobacco products, home and café expenditure, the variety of brands (domestic and imported), flavors, and different weights of waterpipe tobacco packs are needed. Also, own- elasticity and cross-price elasticity of waterpipe tobacco products are required [62].
	One report demonstrated that to ensure sustained commitment from both the governmental side to provide regular and updated data and the public side to use the provided waterpipe tobacco economics data wisely, it would be necessary to develop a written policy that details the responsibilities of the Ministry of Finance for public information sharing and transparency [63].
Uncertainty regarding benefits and potential harms	Literature review did not reveal any uncertainty estimates regarding benefits and potential harms

IV.2 Element 2 – Producing policy-guiding knowledge by modelling the impact of fiscal policies on waterpipe tobacco use prevalence and government revenues

2.1 Utilize data on national waterpipe tobacco economics that will be made available through the official public platforms to model the impact of current fiscal policies based on country-specific information

The review of evidence in the WHO technical manual on tobacco tax policy and administration recommends economic modelling for tobacco taxation [44]. The model would follow previously published models in this field [60,61,64].

Currently, the Eastern Mediterranean Consortium on the Economics of Waterpipe Tobacco Smoking (ECON-WTS) is working on an economic model for waterpipe tobacco tax scenarios for Egypt, Jordan, Lebanon, and Palestine [63]. The simulation model used country- and market share-specific price data, consumption data, and price elasticities. These data were available from recent national surveys that were conducted in 2019/2020 for the three latter countries only. Egypt has national prevalence data from 2017/2018, but does not have any publicly available data on market shares, prices, and price elasticities for waterpipe tobacco on a national level. For each country, incremental changes to the tax structure that mimicked past policy behavior were modeled and specific excise taxes were increased so that 75% of the total retail price constituted tax. The unit of waterpipe tobacco proposed for applying these tax policy improvements in the simulation model is 20 g. Preliminary results of modeling suggest that in comparison to the current tax policy in each country, this increase in taxes simulates 75.8% fewer waterpipe tobacco sessions smoked, 413.6% increased governmental revenues, and 99,168 averted premature deaths in Jordan. In Lebanon, it simulates 97.9% fewer sessions, 37.5% increased revenue, and 345,757 averted deaths. In Palestine, it simulates 16.2% fewer sessions, 37.5% increased revenue, and 345,757 averted deaths [Manuscript under publication]. These results are subject to further verification and sensitivity analysis.

A similar model for Egypt that is based on national and recent data is urgently needed. The model inputs would consider the a) import prices; b) market shares stratified by location because prices vary substantially depending on whether waterpipe tobacco is smoked at home or in a café, c) the median price at last purchase to categorize consumption into two groups, discount and premium, and d) tax structure including costs, insurance, and freight /ex-factory price, import duty, specific tax, ad valorem tax, value added tax, and industry margin. For each market share, the daily consumption volume would be calculated by multiplying the median number of WTS sessions per day and the median number of hagar per session (assuming 20g per head). Then daily consumption volumes would be multiplied by the adult population and the prevalence of WTS to convert it into a national annual consumption value. Total annual tax revenue would be calculated by multiplying annual waterpipe tobacco consumption values by the total tax. Local elasticity estimates and changes to the underling tax rates would be included to calculate new tax revenues. Using the available data in Egypt to-date, the model simulates approximately 50% reduction in WTS, three-folds increase in governmental revenue, and a quarter fewer premature deaths relative to the base case scenario. However, sensitivity analysis is being currently run to refine elasticity estimates [Manuscript under preparation].

2.2 Calculate the health and economic benefits of recommended fiscal policies and waterpipe tobacco tax system reform

The review of evidence in the WHO technical manual on tobacco tax policy and administration recommends proposing different scenarios for taxation [44,65]. The ECON-WTS Consortium proposes two scenarios for applying projections of the economic benefits of reforming waterpipe tobacco tax systems: the first scenario models a status quo approach that uses the same tax change

as in previous years. The second scenario models a tax change that would meet the WHO recommendation to ensure taxes make up at least 75% of the retail price [44]. This increase would be built on the specific excise tax component as the WHO advise that this is simpler and more effective than increasing ad valorem excise [44].

Other scenarios could be developed based on the previous successful experience of Egypt during 2009-2010 in modelling cigarette tax by a close collaboration between the Ministry of Finance, Ministry of Health, and the WHO [17,47]. The evidence generated by the waterpipe tobacco tax models will be distributed to concerned officials to enable policymakers to select the most efficient scenario that will lead to the maximum health and economic benefits.

Table 5. Key findings from systematic reviews and single studies

**Category of finding**	**Element 2**
Benefits	One systematic review recommends economic modelling for tobacco taxation [44].<br><br>Three studies demonstrated the methodology that the model be conducted in the field of cigarette taxation [60,61,64].<br><br>One study revealed that Eastern Mediterranean Consortium on the Economics of Waterpipe Tobacco Smoking (ECON-WTS) is working on an economic model for waterpipe tobacco tax scenarios for Egypt, Jordan, Lebanon, and Palestine [63].
Harms	Literature review did not reveal any potential direct harms
Cost and/ or cost effectiveness in relation to the status quo	There were no studies available in the literature about the direct cost of an economic model formulation for a taxation intervention. However, there were studies that demonstrated what scenarios are required for the simulation.
	One Systematic review on tobacco taxation and one study on cigarette taxation recommend proposing different scenarios for taxation [44,65]. One scenario for applying projections of the economic benefits of reforming waterpipe tobacco tax systems: the first scenario models a status quo approach that uses the same tax change as in previous years. The second scenario models a tax change that would meet the WHO recommendation to ensure taxes make up at least 75% of the retail price [44]. This increase would be built on the specific excise tax component as the WHO advise that this is simpler and more effective than increasing ad valorem excise [44].
Uncertainty regarding benefits and potential harms	Literature review did not reveal any uncertainty estimates regarding benefits and potential harms

IV.3. Element 3 – Improving current waterpipe tobacco taxation system through raising its level and reforming its structure, guided by the modeling in Element 2

3.1 Raise the waterpipe tobacco tax level based on the evidence generated from country-specific models and adherent to the WHO FCTC Article 6 guidelines

Systematic reviews recommend improving taxation policies as a cost-effective tool to combat tobacco use and provide equity in health and economic benefits [66-70]. One systematic review reported that a 10% increase in waterpipe tobacco taxation in Lebanon would reduce waterpipe tobacco demand by 14.5% (price elasticity of demand -1.45) [66].

Local evidence from Egypt demonstrates that regular increases in tobacco taxes over years have led to increased governmental revenues [17,47]. In the fiscal year 2020/2021, tobacco taxes revenues were EGP 75 billion (USD 4.6 billion) constituting nearly 8% of total governmental revenues [15,50-52]. Previously, the 2010 improvements in cigarette taxes resulted in a 151% increase in revenues from EGP 7 to EGP 17.6 billion between 2010 and 2012, a 14% decrease in tobacco sales within two years, and a 46% increase in the cigarette tax per pack for the most popular brand [17,47].

3.2 Conduct modifications to the waterpipe tobacco tax structure by including a specific excise component and a uniform one-tiered structure using the evidence generated from country-specific models and the recommended WHO FCTC Article 6 guidelines

The review of evidence in the WHO technical manual on tobacco tax policy and administration recommends including a specific excise component within a simple tax structure [44]. Currently, evidence on the relationship between tax structure and WTS is unavailable. However, for cigarette consumption, recent evidence suggests that a specific and uniform tax structure is superior to an ad valorem and tiered tax structure in reducing cigarette consumption by 6-65% [60]. Similarly, a unform and specific tax structure for waterpipe tobacco is expected to be the most effective in reducing waterpipe tobacco consumption.

Article 6 of the WHO FCTC guidelines recommend that the tobacco tax structure includes a specific excise component rather than ad valorem because specific tax leads to higher prices and a lower market share of cheap products [43,44]. In an ad valorem system, a minimum specific tax should also be implemented to guarantee minimum price and revenue levels [44]. In addition, the tax structure needs to be uniform not differential because uniform tax leads to larger reductions in smoking as there is less opportunity to switch between different tiers and types of tobacco products.

Table 6. Key findings from systematic reviews and single studies

Category of finding	Element 3
Benefits	Five systematic reviews on cigarette taxation recommend improving taxation policies as a cost-effective tool to combat tobacco use and provide equity in health and economic benefits [66-70].<br><br>One systematic review recommends including a specific excise component within a simple tax structure [44].

	Currently, evidence on the relationship between tax structure and WTS is unavailable.<br>However, for cigarette consumption, one cross-country study suggests that a specific and uniform tax structure is superior to an ad valorem and tiered tax structure in reducing cigarette consumption by 6-65% [60].<br><br>One systematic review and one global legally binding article recommend that the tobacco tax structure includes a specific excise component rather than ad valorem because specific tax leads to higher prices and a lower market share of cheap products [43,44].<br><br>One systematic review demonstrates that in an ad valorem system, a minimum specific tax should also be implemented to guarantee minimum price and revenue levels [44]. In addition, the tax structure needs to be uniform not differential because uniform tax leads to larger reductions in smoking as there is less opportunity to switch between different tiers and types of tobacco products [44].
Harms	Literature review did not reveal any potential direct harms
Cost and/ or cost effectiveness in relation to the status quo	One systematic review on waterpipe tobacco interventions reported that a 10% increase in waterpipe tobacco taxation in Lebanon would reduce waterpipe tobacco demand by 14.5% (price elasticity of demand -1.45) [66].<br><br>One local report on cigarette taxation from Egypt demonstrates that regular increases in tobacco taxes over years have led to increased governmental revenues [17,47]. In the fiscal year 2020/2021, tobacco taxes revenues were EGP 75 billion (USD 4.6 billion) constituting nearly 8% of total governmental revenues [15,50-52]. Previously, the 2010 improvements in cigarette taxes resulted in a 151% increase in revenues from EGP 7 to EGP 17.6 billion between 2010 and 2012, a 14% decrease in tobacco sales within two years, and a 46% increase in the cigarette tax per pack for the most popular brand [17,47].
Uncertainty regarding benefits and potential harms	Literature review did not reveal any uncertainty estimates regarding benefits and potential harms

V. Implementation Considerations

Some considerations need to be addressed at different stakeholder levels to overcome potential barriers that may influence successful implementation of the recommended change in waterpipe tobacco taxation policies. Evidence-based counterstrategies to overcome these barriers are suggested.

The different levels addressed with these implementation considerations are the consumer (the smoker of waterpipe tobacco), the professionals (healthcare providers), the organization (national

and regional), and the system (government/Ministry of Finance). These implementation considerations may be summarized as follows:

Levels	Barriers	Counterstrategies
Consumer level:	The smokers' barriers could be unwillingness to pay extra taxes and lack of awareness of the public health and economic benefits [70].	Raising public awareness that tobacco taxes are re-directed in health investment [70].<br><br>Public support for increased taxation increases substantially when tax revenues are specifically directed to fund public health programs [70].<br><br>National and regional organizations that advocate for health could make parallel efforts to create public awareness for the positive impact of increasing taxes on health and economy, as well as be involved in smoking cessation awareness campaigns [44, 65-70]. The government and corporate social responsibility programs could fund and assist these organizations in this regard.
Professional level:	Health care provider barriers could be that they are not well trained on smoking cessation as more smokers are expected to ask for professional advice on how to quit WTS because of price increases in waterpipe tobacco products [66].	Implement a national support program, including training of health care providers on smoking cessation and behavioral counseling, especially in primary health care [66].
Organization level:	Inefficient and ineffective administration monitoring of tobacco tax systems hinders tax compliance and collection of tax revenue, increasing the risk of tax evasion and illicit trade [44].	Institutional arrangements:<br>Clearly defined roles of competent authority, designed to prevent overlaps and voids [44].<br>-Effective coordination among relevant bodies within a country and across jurisdictions are essential to optimize tax collection and enforcement of tax policy (national, bilateral across borders, regional, and international coordination) [44].<br>-Evaluation of performance and accountability. Key strategic indicators are useful for assessing the performance of a competent authority, including the cost of collection ratio,

		tax gap analysis and tax revenue targets [44].<br><br>Tax compliance cycle:<br>For any tax, there are associated compliance, control, and enforcement processes. The compliance cycle usually includes registration and licensing, tax declarations, recordkeeping, storage in warehouses, duty suspension, collection of tax and tax refunds. **Figure 5** illustrates the typical stages of the tax compliance cycle [44].<br><br>Control and enforcement planning:<br>-Establish a strategic plan to control the compliance cycle by ensuring taxpayers compliance and preventing illicit trade and tax avoidance, preferably focusing the majority of resources on preventive policy [44].<br>- Tax risk management through risk analysis to identify the points of intervention that have higher probabilities of noncompliance. A risk-based approach with targeted interventions allows for better results and more efficient use of resources to ensure effectiveness of tax collection. An enforcement and control plan must be drafted. This plan should include definitions of the activities that will be enforced, the taxpayers upon whom they will be enforced and the circumstances under which they will be enforced, as well as allocating resources for staffing, auditing, infrastructure, and IT [44]. Targets must also be defined, including the number of interventions and the amount of additional collected revenue or reduction of tax evasion. Modern risk assessment makes use of electronic data on taxpayers, tax payments,

		declarations from other taxes, such as VAT, and third-party information [44].<br><br>The government could require taxpayers to present this information among the obligatory tax declarations.<br><br>Controls over the supply chain:<br>The main places for reporting and monitoring along the supply chain: import, ex-factory and removals from warehouses. Manufacturers could be required to report imported inputs at the border, as importers of finished products do. If components are subject to licensing, information can be required as part of the licensing process [44].<br><br>Licencing and due diligence:<br>Licensing provides timely and accurate data that can serve as the basis for audits because it identifies and controls legitimate operators. For new operators, the process to obtain a licence could include visits and verification of production factories, storage facilities and distribution premises. Licensing helps to identify and control legitimate operators. The data obtained from licensing can serve as a basis for audits. Licences should be controlled on a regular basis and updated periodically to ensure their validity [44].<br><br>Fiscal markings (Tax stamps):<br>The use of fiscal markings is generally considered to be an appropriate tool for increasing compliance with tax laws. Fiscal markings can also be helpful for distinguishing between genuine and illicit tobacco products [44].<br><br>Tracking and tracing:

		Tracking and tracing system assists authorities in determining the origin of tobacco products and the point of diversion, as well as monitoring and controlling the movement of tobacco products and their legal status [44].<br><br>Anti-forestalling:<br>Forestalling reduces and delays the effectiveness of tax measures. Implementing anti-forestalling measures can limit the delay of a tax increase and its intended effect on revenues and consumer behaviour [44].<br><br>Additional national audits and controls:<br>Several different types of periodic audits and controls that can be carried out to increase compliance, including cost audits, transfer pricing audits, price and market monitoring, consumer controls and cross-check controls [44].<br><br>Import and export controls:<br>To ensure control of import and export, it is recommended that only<br>duly licensed persons and entities be allowed to import and export tobacco products and manufacturing equipment [44].<br><br>Free zones and transhipment points:<br>Customs administrations should exercise their authority in free zones to prevent different economic operations from taking place outside the control of authorities. Relevant measures include licensing, due diligence and recordkeeping for all operators within free zones, as well as implementing a tracking and tracing regime [44].<br><br>Procedures after detecting illicit trade of tobacco:<br>As soon as smuggling or illicit trade in tobacco products is detected, actions

	Implementation<br>Officials may be hesitant to apply major tax changes at once.<br><br>The unit of waterpipe tobacco taxation is unclear and not linked to the real-life consumption pattern of smokers, unlike cigarettes.	such as collecting taxes and seizing and destroying smuggled and/or illicit tobacco must be taken [44].<br><br>Penalties:<br>Penalties and sanctions imposed should be sufficient to deter illegal tobacco trade activities. Penalties should be levied in amounts proportionate to lost taxes and duties resulting from illicit trade [44].<br><br>Consider a phased approach in implementing the suggested waterpipe tobacco tax restructuring policy. This could be done through adopting the successive increments in specific excise tax suggested in ECON-WTS economic model.<br><br>The unit of waterpipe tobacco taxation could be the 20g of tobacco that is unit basis of the hagar used in a WTS session by smokers, and as reported by the WHO report on the global tobacco epidemic [57].<br><br>Also, it is advised to continue taxing the tobacco product itself because a large proportion (51.6%) of waterpipe tobacco smokers smoke it at home [6]. This became more evident during the COVID-19 pandemic where cafes serving waterpipes were shut down by the government [32,33], so smokers shifted to home WTS, considering it a cheaper alternative than café WTS as no value added taxes are applied for the service in cafés.
System level:	***Tobacco industry interference*** The tobacco industry uses SCARE tactics to dissuade governments from implementing	

	tobacco tax increases [44]:<br><br>- (S) **Smuggling and illicit trade** The local industry could raise arguments that governmental revenues will decrease due to the high elasticity, if prices and tax rates of tobacco products increase, and that illicit trade will increase. Officials may be concerned about the problem of smuggling and tax evasion. Illicit trade in tobacco products continues to be a major concern for tax administrators because of the difficulties associated with accurate and independent measurement of it, as well as with its elimination. Industry figures provide a distorted understanding of the extent of the problem, along with a monocausal explanation of the link between illicit trade and tobacco taxation [44].	-Assess independently and with the best statistical practices the size of the illicit trade to assess the scope of the problem [44].<br>-Address directly the country-specific institutional and/or governance challenges, including multilateral coordination, and improve tax and customs administrations practices [44].<br>-Implement best practices to fight illicit trade, contained in the WHO FCTC Protocol to Eliminate Illicit Trade in Tobacco Products [44], which Egypt has signed in January 2021 (Presidential Decree No. 170/2020) [45] and this will strengthen the position of market monitoring sectors. Taxation policies need to be accompanied by a strong law enforcement mechanism to reduce smuggling or tax evasion [44].
	**(C) Court and legal challenges**<br>Health-protective and non-discriminatory tobacco excise taxes are legally defensible, and industry threats will usually be baseless. The Government's legal position can be strengthened, however, by exercising care with a tax measure's procedure, design, and consultation [44].	-Determine the standard of consultation required under domestic law and any applicable international obligations [44].<br>-Distance the tobacco industry from the policy-making process to the extent that this is permissible [44].<br>-Avoid unnecessary and unjustified discrimination towards foreign tobacco products or investors in<br>the design, implementation, or enforcement of a tax measure [44].<br>-Do not offer investment incentives in the form of inducements or contractual undertakings, as these may be binding in and of themselves or grounds for a challenge under an international investment agreement [44].<br><br>The Ministry of Finance has previously submitted an official complaint against

		the local industry, as they ignored inserting the tax stamp required by the Ministry on some tobacco packs [47]. As a result of this complaint, the police confiscated these products and the industry was held liable for compensation due to tax evasion [47]. The Customs Authority filed an official civil complaint and was seen by the court [47]. Such counterstrategies could be used in case the industry interferes with the recommended waterpipe tobacco tax system.
	**(A) Anti-poor rhetoric (regressivity)**<br>In its effort to lobby against tax increases, the tobacco industry often claims that tobacco taxation will hurt the poor. This argument is based on the concept of regressivity in relation to taxation. Conceptually, a tax is regressive if it means lower-income people must pay a relatively greater proportion of their household income to meet the tax liability than wealthy people [44].	Tobacco taxation and tax increases are actually a progressive or pro-poor policy once these wider considerations are properly accounted for [44].<br><br>-The concept of regressivity based solely on tax burden does not consider the wider health and economic harms caused by tobacco use that are disproportionately experienced by lower socioeconomic groups [44].<br>-Higher tobacco taxes and prices can induce behavioral change in the population, as reflected in the price elasticity of demand, which means that lower-income smokers will curtail their smoking the most and thus will benefit disproportionately in terms of health gains from reduced tobacco consumption and use [44].<br>Tax increases, even in countries with already high taxes, bring in additional revenue [44].
	**(R) Revenue reduction**<br>Arguments by tobacco control opponents that tax increases will not result in increases in revenue [44].	If tax increases are carefully designed and tax administration is functional, it is extremely unlikely that tax increases will lead to revenue decreases [44]. For instance, the relatively price inelastic nature of cigarette demand, combined with the low tax share and no overshifting of the tax, means that for

	(E) Employment<br>The tobacco industry often seeks to frame tobacco taxes as an economic issue rather than a public health issue. Particular emphasis is placed on the alleged threat tax increases pose to employment in tobacco manufacturing, as well as related industries. The tobacco industry exaggerates the importance of tobacco employment relative to total national employment and overstates the impact that domestic demand reduction from local taxes will have on tobacco producers serving a global market [44].	most, if not all, countries, increases in revenues will accompany increases in taxes [44].<br><br>The argument used by the industry ignores the fact that expenditures on tobacco do not disappear but rather are redistributed to other consumption that can produce a similar or higher number of jobs [44]. Case studies demonstrate the possibility and methods for governments to support producers in transitioning to other industries that provide similar and often better returns with greater sustainability [44].

Figure 5. Tax compliance cycle [44]

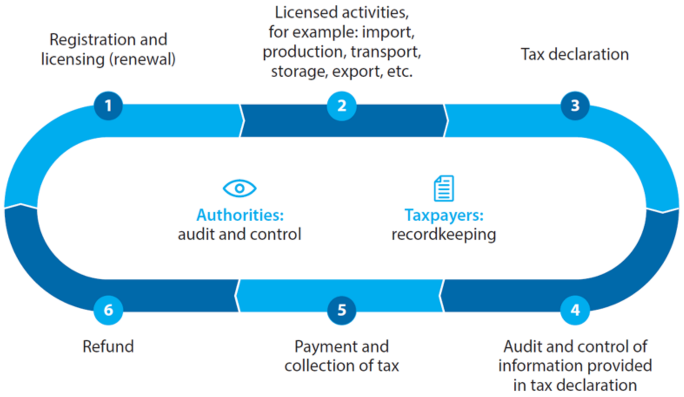

Next Steps

The aim of this Policy Brief is to foster dialogue informed by the best available evidence.
The intention is not to advocate specific policy elements or close off discussion. Further actions will flow from the deliberations that the policy brief is intended to inform. These may include:

→ Deliberation amongst policymakers and stakeholders regarding the policy elements described in this policy brief.

→ Refining elements, for example by incorporating, removing or modifying some components.

References

1. World Health Organization Framework Convention on Tobacco Control. Control and prevention of waterpipe tobacco products 2016. Report by the Convention secretariat FCTC/COP/ 7/10. http://www.who.int/fctc/cop/cop7/FCTC_COP_7_10_EN.pdf?ua=1. Accessed 14 September 2021.

2. World Health Organization 2021. WHO global report on trends in prevalence of tobacco use 2000–2025, 4th ed. https://apps.who.int/iris/handle/10665/348537. Accessed 29 March 2022.

3. Alwatan News. 02 March 2022. Addiction Treatment and Abuse Fund: The smoking rate among secondary school students is 12.8%. https://www.elwatannews.com/news/details/5975539. Accessed 29 March 2022.

4. Shisha and Smokeless Tobacco Use among University Students in Egypt: Prevalence, Determinants, and Economic Aspect. A Joint Report by the Egyptian Ministry of Health and Population and the World Health Organization 2014. http://applications.emro.who.int/dsaf/EMROPUB_2014_EN_1752.pdf?ua=1. Accessed 18 September 2021.

5.World Health Organization. Egypt Global Youth Tobacco Survey 2014. https://extranet.who.int/ncdsmicrodata/index.php/catalog/289. Accessed 15 September 2021.

6.Egypt National STEPwise Survey for Noncommunicable Diseases Risk Factors Report 2017. A Joint Report by the Egyptian Ministry of Health and Population and the World Health Organization. 2017. https://www.who.int/ncds/surveillance/steps/Egypt_National_STEPwise_Survey_For_Noncommunicable_Diseases_Risk_Factors_2017_Report.pdf?ua=1. Accessed 18 September 2021.

7.Hanafy K, Saleh ASE, Elmallah MEBE, et al. The Economics of Tobacco and Tobacco Taxation in Egypt; International Union against Tuberculosis and Lung Disease: Paris, France, 2010. https://www.tobaccofreekids.org/assets/global/pdfs/en/Egypt_tob_taxes_en_F.pdf. Accessed 15 September 2021.

8.Waziry Rm Jawad M, Ballout RA, et al. The effects of waterpipe tobacco smoking on health outcomes: An updated systematic review and meta-analysis. Int. J. Epidemiol. 2017, 46, 32–43,

doi:10.1093/ije/dyw021.

9.Mostafa, A. Self-reported addiction to and perceived behavioural control of waterpipe tobacco smoking and its patterns in Egypt: Policy implications. East Mediterr. Health J. 2020, 26, 18–28, doi:10.26719/2020.26.1.18.

10. Eissenberg, T. Now is the Time for Effective Regulation Regarding Tobacco Smoking Using a Waterpipe (Hookah). J. Adolesc. Health 2019, 64, 685–686, doi:10.1016/j.jadohealth.2019.03.011.

11. Al Oweini D, Jawad M, Akl EA. The association of waterpipe tobacco smoking with later initiation of cigarette smoking: A systematic review and meta-analysis exploring the gateway theory. Tob. Control 2019, doi:10.1136/tobaccocontrol-2018-054870.

12.U.S. Department of Health and Human Services. Smoking Cessation. A Report of the Surgeon General. Atlanta, GA: U.S. Department of Health and Human Services, Centers for Disease Control and Prevention, National Center for Chronic Disease Prevention and Health Promotion, Office on Smoking and Health, 2020. https://www.hhs.gov/sites/default/files/2020-cessation-sgr-full-report.pdf

13. Sustainable development knowledge platform. Sustainable development goal 3. Progress of Goal 3 in 2017. United Nations: https://sustainabledevelopment.un.org/sdg3. Accessed 19 September 2021.

14. Vision of Egypt 2030. https://mped.gov.eg/EgyptVision. Accessed 19 September 2021.

15. Al-Aharam gate. February 2021. Ministry of Finance announces that taxes constitute 75% of governmental revenues. https://gate.ahram.org.eg/News/2612367.aspx . Accessed 18 September 2021.

16. Alwatan News. 08 August 2021. In numbers. taxes and fees for domestic, foreign and electronic cigarettes. Yasser Omar: Tobacco tax increases under the guidance of the World Health Organization. https://www.elwatannews.com/news/details/5626996. Accessed 29 March 2022.

17. Needs assessment for implementation of the WHO Framework Convention on Tobacco Control in Egypt. October 2017. Unpublished report.

18. World Health Organization. Regional Office for the Eastern Mediterranean. Tobacco Free Initiative. Economics. Tobacco tax: Egypt. http://www.emro.who.int/tfi/publications/economics.html. https://applications.emro.who.int/docs/WHOEMTFI209E-eng.pdf?ua=1. Accessed 14 September 2021.

19. Jawad M, Charide R, Waziry R, et al. The prevalence and trends of waterpipe tobacco smoking: A systematic review. PLoS One 2018;13(2):e0192191.

20. An Overview of Global Regulatory Practices in Controlling Waterpipe Tobacco Use. WHO FCTC Secretariat's Knowledge Hub on Waterpipe Tobacco Smoking. July 2018. https://fctc.who.int/who-fctc/governance/conference-of-the-parties/eighth-session-of-the-conference-of-the-parties/supplementary-information/an-overview-of-global-regulatory-practices-in-controlling-waterpipe-tobacco-use . Accessed 14 September 2021.

21. El Awa F, Fouad H, El Naga RA, et al. Prevalence of tobacco use among adult and adolescent females in Egypt. East Mediterr Health J. 2013 Aug;19(8):749-54. PMID: 24975361.

22. Mostafa, A.; El Houssinie, M.; Aboul Foutouh, A. Multiple Tobacco Use among Young Adult Waterpipe Smokers in Egypt. East Mediterr. Health J. 2018, 24, 7–17, doi:10.26719/2018.24.1.17.

23. World Health Organization. NCD Microdata Repository. Global Adult Tobacco Survey, Egypt 2009. Central Agency for Public Mobilization and Statistics, Ministry of Health, Egypt WHO Office. https://extranet.who.int/ncdsmicrodata/index.php/catalog/256/download/2153. Accessed 29 March 2022.

24. The 2017/2018 Household Income, Expenditure and Consumption Survey. Central Agency for Public Mobilization and Statistics: https://www.capmas.gov.eg/Admin/Pages%20Files/2019123101612income1.pdf. Accessed 20 September 2021.

25. World Health Organization. Regional Office for the Eastern Mediterranean. 2006. Tobacco use in shisha: studies on waterpipe smoking in Egypt. https://apps.who.int/iris/handle/10665/119837. Accessed 29 March 2022.

26. Akl EA, Jawad M, Lam WY, et al. Motives, beliefs and attitudes towards waterpipe tobacco smoking: a systematic review. Harm Reduct J. 2013 Jul 2;10:12. doi: 10.1186/1477-7517-10-12.

27. Akl EA, Ward KD, Bteddini D, et al. The allure of the waterpipe: a narrative review of factors affecting the epidemic rise in waterpipe smoking among young persons globally. Tobacco Control 2015;24:i13-i21.

28. Khalil J, Afifi R, Fouad FM, et al. Women and waterpipe tobacco smoking in the eastern mediterranean region: allure or offensiveness. Women Health. 2013;53(1):100-16. doi: 10.1080/03630242.2012.753978.

29. Hawari FI, Obeidat NA, Abu Alhalawa M, et al. Respiratory health and quality of life in young exclusive, habitual smokers - a comparison of waterpipe smokers, cigarette smokers and

non-smokers. Int J Chron Obstruct Pulmon Dis. 2019 Aug 13;14:1813-1824. doi: 10.2147/COPD.S205050.

30. Mortaz, E., Alipoor, S.D., Movassaghi, M. et al. Water-pipe smoke condensate increases the internalization of Mycobacterium Bovis of type II alveolar epithelial cells (A549). BMC Pulm Med 17, 68 (2017). https://doi.org/10.1186/s12890-017-0413-7.

31. World Health Organization. Regional Office for the Eastern Mediterranean. Countries. Tobacco Free initiative. Know the truth. Tobacco and waterpipe use increases the risk of COVID-19. http://www.emro.who.int/tfi/know-the-truth/tobacco-and-waterpipe-users-are-at-increased-risk-of-covid-19-infection.html#:~:text=Since%20waterpipe%20smoking%20is%20typically,COVID%2D19%20in%20social%20gatherings. Accessed 28 March 2022.

32. Alarabya. 16 March 2020. Egypt faces Corona. Shisha ban and the closure of popular markets. https://www.alarabiya.net/arab-and-world/egypt/2020/03/16/ مصر-تواجه-كورونا-وقف-الرحلات-الجوية-واغلاق-الأسواق. Accessed 29 March 2022.

33. Ahram gate. 10 December 2021. Banning hookah, banning entry to facilities, procedures for those coming to Egypt, and punishment for violators. Government decisions to confront Corona. https://gate.ahram.org.eg/News/3183561.aspx. Accessed 29 March 2022.

34. The Egyptian Ministry of Health and Population. 18 August 2020. Smoking hookah increases your chance of contracting COVID-19...stop smoking or sharing it with others.https://www.facebook.com/113432613540693/photos/a.122315979319023/212332210317399/?type=3. Accessed 29 March 2022.

35. Safizadeh H, Moradi M, Rad MR, Nakhaee N. Bacterial contamination of different components of the waterpipe. Int J Tuberc Lung Dis. 2014 Aug;18(8):988-91. doi: 10.5588/ijtld.14.0026.

36. Primack BA, Carroll MV, Weiss PM, et al. Systematic Review and Meta-Analysis of Inhaled Toxicants from Waterpipe and Cigarette Smoking. Public Health Rep. 2016 Jan-Feb;131(1):76-85. doi: 10.1177/003335491613100114.

37. Jawad M, Eissenberg T, Salman R, et al. Toxicant inhalation among singleton waterpipe tobacco users in natural settings. Tob Control. 2019 Mar;28(2):181-188. doi: 10.1136/tobaccocontrol-2017-054230.

38. Shihadeh A, Schubert J, Klaiany J, et al. Toxicant content, physical properties and biological activity of waterpipe tobacco smoke and its tobacco-free alternatives. Tob Control. 2015 Mar; 24 Suppl 1(Suppl 1):i22-i30. doi: 10.1136/tobaccocontrol-2014-051907.

39. World Health Organization. Regional Office for the Eastern Mediterranean. Countries. Egypt. Programme areas. Tobacco. Tobacco Health Cost in Egypt 2014: https://applications.emro.who.int/dsaf/EMROPUB_2014_EN_1750.pdf?ua=1. Accessed 20 September 2021.

40. Alsultah. 02 December 2019. Egypt's Observatory for Tobacco Control: Egyptians consume 80 billion cigarettes and 50 tonnes of waterpipe tobacco annually: https://www.alsolta.net/article/84830/article/146857/. Accessed 20 September 2021.

41. World Health Organization. Publications. Overview. Tobacco Egypt 2021 country profile. https://www.who.int/publications/m/item/tobacco-egy-2021-country-profile. Accessed 29 March 2022.

42. Law No. 154 of 2007 regarding the amendment of some provisions of Law No. 52 of 1981 concerning the prevention of the harms of smoking. http://www.emro.who.int/images/stories/tfi/documents/law_egy_2007.154.pdf?ua=1&ua=1. Accessed 29 March 2022.

43. World Health Organization. Guidelines for implementation of Article 6 of the WHO FCTC. https://www.who.int/fctc/guidelines/adopted/Guidelines_article_6.pdf. Accessed 20 September 2021.

44. World Health Organization. 2021. WHO technical manual on tobacco tax policy and administration. World Health Organization. https://apps.who.int/iris/handle/10665/340659. Accessed 11 November 2021.

45. State Information Services. Your gateway to Egypt. Home. Presidency. Presidential Decrees. https://sis.gov.eg/Story/154025/Presidential-decree-to-eliminate-illicit-trade-in-tobacco-products?lang=en-us. Accessed 20 September 2021.

46. World Health Organization. Regional Office for the Eastern Mediterranean. Countries, Egypt. Seminar on applying the banderole system in Egypt: http://www.emro.who.int/tobacco/seminar-banderole-egypt.html. Accessed 20 September 2021.

47. Personal communication. Former focal point of tobacco control in the Ministry of Finance. October 2021.

48. Jawad M, McEwen A, McNeill A, et al. To what extent should waterpipe tobacco smoking become a public health priority? Addiction. 2013 Nov;108(11):1873-84. doi: 10.1111/add.12265.

49. World Health Organization. Framework Convention on Tobacco Control. July 2020. The Guide for WHO FCTC Parties on including SDG Target 3.a in Voluntary National Reviews. https://www.who.int/fctc/VNR_Guide_7July2020.pdf. Accessed 29 March 2022.

50. Ministry of Finance. Egypt. September 2021. Citizen version of the approved budget 2021/2022. http://www.budget.gov.eg/pdf/2021/CB%20Final%20Version.pdf. Accessed 29 March 2022.

51. Youm 7. 16 November 2021. The final account of the 2020/2021 budget. Total revenues of 1.108 trillion pounds. Minister of Finance to the House of Representatives: 834 billion tax revenues. 270.8 billion tax arrears and the application of the electronic invoice next April. www.youm7.com/story/2021/11/16/الحساب-الختامى-لموازنة-2020-2021-إجمالى-الإيرادات-1-108-تريليون/5539304. Accessed 29 March 2022.

52. Independent Arabia. 18 January 2021: Egypt increases prices of cigarettes and waterpipe tobacco next July.: https://www.independentarabia.com/node/186051/اقتصاد/الاقتصاد-العربي/مصر-ترفع-أسعار-السجائر-والدخان-في-يوليو-المقبل. Accessed 13 September 2021.

53. Law No. 13 of 2020 for amending some provisions of the tax law on the added value promulgated by Law No. 67 of 2016. https://www.laweg.net//Default.aspx?action=ViewActivePages&ItemID=115298&Type=6. Accessed 29 March 2022.

54. World Health Organization. Publications. Overview. WHO report on the global tobacco epidemic, 2015: raising taxes on tobacco. https://www.who.int/publications/i/item/9789241509121. Accessed 29 March 2022.

55. World Health Organization. Publications. Overview. WHO report on the global tobacco epidemic, 2017: monitoring tobacco use and prevention policies. https://www.who.int/publications/i/item/9789241512824. Accessed 29 March 2022.

56. World Health Organization. Publications. Overview. WHO report on the global tobacco epidemic 2019: offer help to quit tobacco use. https://www.who.int/publications/i/item/9789241516204. Accessed 29 March 2022.

57. World Health Organization. Health Promotion. WHO report on the global tobacco epidemic 2021: addressing new and emerging products. https://www.who.int/teams/health-promotion/tobacco-control/global-tobacco-report-2021. Accessed 20 September 2021.

58. World Health Organization. The Global Health Observatory, Tobacco tax structure - excise tax as a proportion of price 2021: https://www.who.int/data/gho/indicator-metadata-registry/imr-details/4608. Accessed 20 September 2021.

59. World Health Organization. Tobacco Free Initiative. Tobacco tax levels and structure. Chapter II: Tobacco tax levels and structure: A theoretical and empirical overviewhttps://www.who.int/tobacco/publications/en_tfi_tob_tax_chapter2.pdf. Accessed 13 September 2021.

60. Shang C, Lee HM, Chaloupka FJ, et al. Association between tax structure and cigarette consumption: findings from the International Tobacco Control Policy Evaluation (ITC) Project. Tobacco Control 2019;28:s31-s36. http://dx.doi.org/10.1136/tobaccocontrol-2017-054160.

61. Tesche J, Van Walbeek C. Measuring the effects of the new ECOWAS and WAEMU tobacco excise tax directives. Tob Control. 2020 Sep 28:tobaccocontrol-2020-055843. doi: 10.1136/tobaccocontrol-2020-055843.

62. Chalak A, Nakkash R, Abu-Rmeileh NME, et al. Own-price and cross-price elasticities of demand for cigarettes and waterpipe tobacco in three Eastern Mediterranean countries: a volumetric choice experiment. Tob Control. 2021 Jun 30:tobaccocontrol-2021-056616. doi: 10.1136/tobaccocontrol-2021-056616.

63. World Health Organization Framework Convention on Tobacco Control. Home. Publications. Overview. WHO Framework Convention on Tobacco Control. https://apps.who.int/iris/bitstream/handle/10665/42811/9789246591015_ara.pdf. Accessed 29 March 2022.

64. World Health Organization. WHO FCTC Knowledge Hub on Tobacco Taxation & Illicit Trade. https://untobaccocontrol.org/kh/taxation/. Accessed 21 September 2021.

65. Schafferer C, Yeh CY, Chen SH, et al. A simulation impact evaluation of a cigarette excise tax increase on licit and illicit cigarette consumption and tax revenue in 36 European countries. Public Health. 2018;162:48–57. doi: 10.1016/j.puhe.2018.05.017.

66. Jawad M, Jawad S, Waziry RK, Ballout RA, Akl EA. Interventions for waterpipe tobacco smoking prevention and cessation: a systematic review. Sci Rep. 2016 May 11;6:25872. doi: 10.1038/srep25872.

67. Brown T, Platt S, Amos A. Equity impact of population-level interventions and policies to reduce smoking in adults: a systematic review. Drug Alcohol Depend. 2014 May 1;138:7-16. doi: 10.1016/j.drugalcdep.2014.03.001. Epub 2014 Mar 13. PMID: 24674707.

68. Brown T, Platt S, Amos A. Equity impact of interventions and policies to reduce smoking in youth: systematic review. Tob Control. 2014 Nov;23(e2):e98-105. doi: 10.1136/tobaccocontrol-2013-051451. Epub 2014 May 19. PMID: 24842855.

69. World Health Organization. ‘BEST BUYS’ AND OTHER RECOMMENDED INTERVENTIONS FOR THE PREVENTION AND CONTROL OF NONCOMMUNICABLE DISEASES UPDATED (2017) APPENDIX 3 OF THE GLOBAL ACTION PLAN FOR THE PREVENTION AND CONTROL OF NONCOMMUNICABLE DISEASES 2013-2020 https://www.who.int/ncds/management/WHO_Appendix_BestBuys_LS.pdf?ua=1 Accessed 21 September 2021.

70. Wright, A., Smith, K.E. & Hellowell, M. Policy lessons from health taxes: a systematic review of empirical studies. BMC Public Health 17, 583 (2017). https://doi.org/10.1186/s12889-017-4497-z.

71. Loffredo CA, Tang Y, Momen M, Makambi K, Radwan GN, Aboul-Foutoh A. PM2.5 as a marker of exposure to tobacco smoke and other sources of particulate matter in Cairo, Egypt. Int J Tuberc Lung Dis. 2016 Mar;20(3):417-22. doi: 10.5588/ijtld.15.0316.

Glossary[5]

Ad valorem excise tax	means a tax levied on selected products based on value, such as retail selling price, the manufacturer's (or ex-factory) price, or the cost insurance freight price (CIF)
Affordability	means price relative to per capita income
"Excise tax" or "Excise duty"	means a tax or duty imposed on the sale or production of selected products, such as tobacco products
"General sales tax (GST)"	means a tax imposed on a wide variety of products, typically based on retail price
"Import tax" or "Import duty"	means a tax imposed on selected imported products, such as tobacco products
"Mixed tax" or "Hybrid tax"	means a tax that includes both a specific tax component and an ad valorem tax component
"Prevalence"	means the percentage of the population that uses a tobacco product
"Price elasticity of demand"	means the percentage change in consumption resulting from a one per cent increase in real price
"Product substitution"	means switching from the use of one tobacco product to another, for example from cigarettes to loose tobacco, in response to changes in relative prices or other factors
"Share of excise tax in retail price"	means the percentage of the retail price of a tobacco product, inclusive of all relevant taxes, accounted for by excise taxes on that product
"Share of taxes in retail price"	means the percentage of the retail price of a tobacco product, inclusive of all relevant taxes, accounted for by all taxes on that product
"Specific excise tax"	means a tax levied on selected products based on quantity, such as number of cigarettes or weight of tobacco
"Tiered tax"	means a tax applied at different rates to different variants of a given product, based on various factors such as price, product characteristics, or production characteristics

[5] World Health Organization. Guidelines for implementation of Article 6 of the WHO FCTC. https://www.who.int/fctc/guidelines/adopted/Guidelines_article_6.pdf.

“Uniform tax”	means a tax applied at the same rate to all variants of a given product, such as all cigarette brands and brand variants
“Value added tax (VAT)”	means a tax imposed on a wide variety of products (domestic and imported), based on the value added at each stage of production or distribution

© 2023 Nakkash R. et al.
